# ABHD11 maintains 2-oxoglutarate metabolism by preserving functional lipoylation of the 2-oxoglutarate dehydrogenase complex

**DOI:** 10.1038/s41467-020-17862-6

**Published:** 2020-08-13

**Authors:** Peter S. J. Bailey, Brian M. Ortmann, Anthony W. Martinelli, Jack W. Houghton, Ana S. H. Costa, Stephen P. Burr, Robin Antrobus, Christian Frezza, James A. Nathan

**Affiliations:** 1 Cambridge Institute for Medical Research, Department of Medicine, University of Cambridge, Cambridge, CB2 0XY UK; 2 Cambridge Institute of Therapeutic Immunology & Infectious Disease (CITIID), Jeffrey Cheah Biomedical Centre, Cambridge Biomedical Campus, University of Cambridge, Cambridge, CB2 0AW UK; 3 MRC Cancer Unit, University of Cambridge, Cambridge, CB2 0XZ UK; 4 Present Address: Cambridge Institute of Therapeutic Immunology & Infectious Disease (CITIID), Jeffrey Cheah Biomedical Centre, Cambridge Biomedical Campus, University of Cambridge, Cambridge, CB2 0AW UK; 5 Present Address: Cold Spring Harbor Laboratory, Cold Spring Harbor, NY 11724 USA; 6 Present Address: MRC Mitochondrial Biology Unit, University of Cambridge, Cambridge, CB2 0XY UK

**Keywords:** Hydrolases, Nutrient signalling, Energy metabolism, Post-translational modifications

## Abstract

2-oxoglutarate (2-OG or α-ketoglutarate) relates mitochondrial metabolism to cell function by modulating the activity of 2-OG dependent dioxygenases involved in the hypoxia response and DNA/histone modifications. However, metabolic pathways that regulate these oxygen and 2-OG sensitive enzymes remain poorly understood. Here, using CRISPR Cas9 genome-wide mutagenesis to screen for genetic determinants of 2-OG levels, we uncover a redox sensitive mitochondrial lipoylation pathway, dependent on the mitochondrial hydrolase ABHD11, that signals changes in mitochondrial 2-OG metabolism to 2-OG dependent dioxygenase function. ABHD11 loss or inhibition drives a rapid increase in 2-OG levels by impairing lipoylation of the 2-OG dehydrogenase complex (OGDHc)—the rate limiting step for mitochondrial 2-OG metabolism. Rather than facilitating lipoate conjugation, ABHD11 associates with the OGDHc and maintains catalytic activity of lipoyl domain by preventing the formation of lipoyl adducts, highlighting ABHD11 as a regulator of functional lipoylation and 2-OG metabolism.

## Abstract (web summary)

2-oxoglutarate links mitochondrial metabolism to oxygen sensing, gene transcription and epigenetic modifications. Here, the authors describe a mutagenesis screen to find genes that control 2-oxoglutarate levels, and identify ABHD11 as a mitochondrial regulator of 2-oxoglutarate abundance.

## Introduction

The ability to sense and respond to nutrient abundance is a fundamental requirement for cell survival, and to achieve this, cells have evolved several strategies that link metabolic function to transcriptional adaptation. One such strategy is the coupling of 2-oxoglutarate (2-OG) metabolism to gene transcription, whereby 2-OG, a key component of TCA cycle, can facilitate cell function by modulating the activity of 2-OG dependent dioxygenases involved in the hypoxia inducible factor (HIF) response, DNA methylation, and histone modifications^1^.

The relevance of 2-OG in modulating the activity of these dioxygenases is exemplified by changes in the relative abundance of cellular 2-OG. An increased 2-OG/succinate ratio promotes embryonic stem cell pluripotency^2^, and antagonises the growth of solid organ tumours^3^ through increased hydroxymethylation of DNA (5hmC) and histone demethylation. Conversely, elevated cellular 2-OG can drive its own reduction to L-2-hydroxyglutarate (L-2-HG), which counterintuitively inhibits 2-OG dependent dioxygenases, leading to decreased DNA hydroxymethylation and histone demethylation, activation of the HIF response, altered T cell fate, and haematopoietic cell differentiation^4–9^. Consequently, understanding how 2-OG metabolism is regulated has broad biological implications.

Central to maintaining cellular 2-OG homeostasis is the 2-oxoglutarate dehydrogenase complex (OGDHc, also known as the α-ketoglutarate dehydrogenase complex), the rate-limiting enzyme within the TCA cycle that oxidatively decarboxylates 2-oxoglutarate to succinyl-CoA. This evolutionarily conserved enzyme also requires lipoic acid, a redox sensitive cofactor that is synthesised within the mitochondria and conjugated to a single lysine within the OGDHc E2 subunit, dihydrolipoamide S-succinyltransferase (DLST)^10–12^. The cyclical reduction and oxidation of the two thiols of conjugated lipoic acid (lipoamide to dihydrolipoamide) serves as a redox intermediate, coupling the formation of succinyl CoA to generation of NADH. The importance of DLST and its lipoylation is highlighted by the recent identification of genetic mutations leading to human disease. Patients with germline mutations in lipoic acid synthesis genes develop a severe variant of the neurological condition, Leigh syndrome^13^, and loss of heterozygosity mutations in the OGDHc lead to angiogenic tumours (pheochromocytomas and paragangliomas), similar to other hereditary cancer syndromes activating the HIF pathway^14^. However, how OGDHc function and 2-OG abundance is regulated is unclear.

Here, we use the sensitivity of the HIF pathway to 2-OG abundance to gain insights into how 2-OG metabolism is controlled. Using genome-wide CRISPR/Cas9 mutagenesis screens, we identify an uncharacterised protein, αβ-hydrolase domain-containing 11 (ABHD11), as a mitochondrial enzyme that impairs OGDHc activity when depleted or inhibited. ABHD11 loss leads to the accumulation of 2-OG and formation of L-2-HG, which inhibits 2-OG dependent dioxygenases involved in the HIF response and DNA hydroxymethylation, similarly to genetic disruption of the OGDHc. ABHD11 also associates with the OGDHc and is required for catalytic activity and TCA cycle function. However, ABHD11 does not alter the constituent levels of the OGDHc. Instead, ABHD11 maintains functional lipoylation of the OGDHc, preserving the catalytic activity of DLST. Together, these studies identify a key role for ABHD11 in 2-OG metabolism, and demonstrate that lipoylation provides a previously unappreciated mechanism for mediating an adaptive transcriptional response to changes in OGDHc function.

## Results

### ABHD11 mediates activity of 2-OG dependent dioxygenases

To find genes involved in 2-OG metabolism we utilised the sensitivity of the HIF response to 2-OG availability, and carried out CRISPR/Cas9 mutagenesis screens in human cells using a fluorescent HIF reporter we developed^4,15^. This reporter encodes the consensus HIF responsive element (HRE) in triplicate that drives the expression of GFP fused to the oxygen and 2-OG sensitive region of HIF-1α (Supplementary Fig. 1a)^4,15^. Therefore, reporter stability is dependent on 2-OG dependent dioxygenase activity of the prolyl hydroxylases (PHDs or EGLNs)^16,17^, which was confirmed with treatment with the PHD inhibitor dimethyloxalylglycine (DMOG), cell permeable 2-OG (dimethyl 2-OG) or incubation in 1% oxygen (Supplementary Fig. 1b–d)^4,7,15^.

**Fig. 1:**
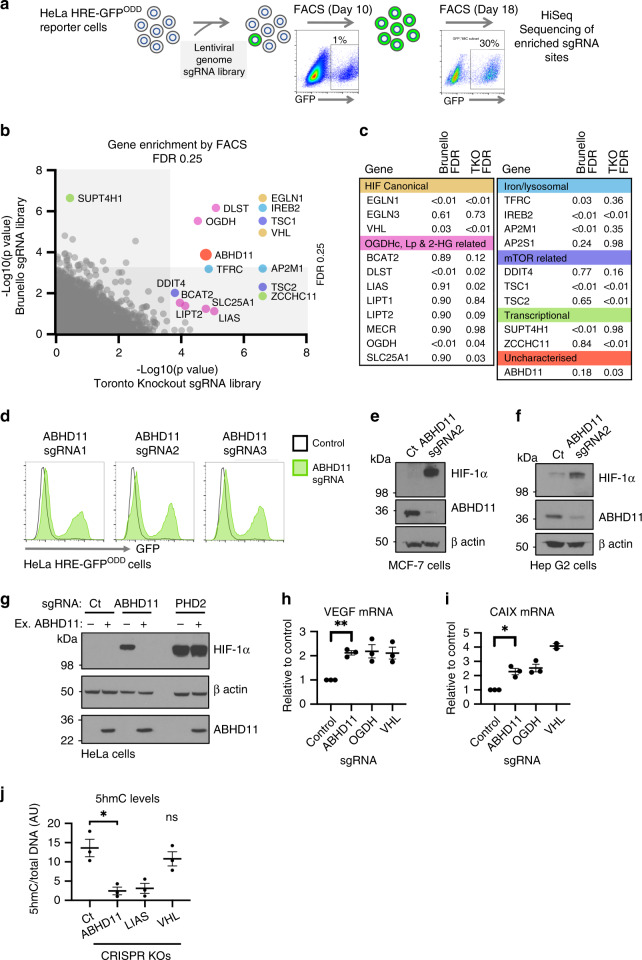
Identification of ABHD11 as a mediator of 2-OG dependent dioxygenase activity. **a** HeLa HRE-GFP^ODD^ cells were transduced and mutagenised with genome-wide sgRNA libraries (Brunello and Toronto KO). GFP^HIGH^ cells were selected by iterative FACS and sgRNA identified by Illumina HiSeq. **b**, **c** Comparative bubble plot (**b**) and table (**c**) of sgRNA enriched in the GFP^HIGH^ cells between the two genome-wide sgRNA libraries compared to a mutagenised population of HRE-GFP^ODD^ cells that had not been phenotypically selected. Genes enriched for sgRNA clustered into six main groups: (1) the canonical HIF pathway, (2) OGDHc, Lipoylation (Lp) and 2-HG related pathways, (3) intracellular iron metabolism (iron, lysosomal), (4) mTOR, (5) Transcription and (6) Uncharacterised. Unadjusted *p* value calculated using MaGECK robust rank aggregation (RRA); FDR = Benjamini-Hochberg false discovery rate (multiple hypothesis adjustment of RRA *p* value). *EGLN1* = *PHD2, EGLN3* = *PHD3*. **d**–**f** HeLa HRE-GFP^ODD^ (**d**), MCF-7 (**e**) and Hep G2 (**f**) cells stably expressing Cas9 were transduced with up to three different sgRNA targeting ABHD11. Reporter GFP or endogenous HIF-1α levels were measured by flow cytometry (**d**) or immunoblot (**e**, **f**) respectively after 10-13 days. Endogenous ABHD11 levels were measured by immunoblot and β-actin served as a loading control. **g** Reconstitution of mixed KO population of ABHD11 with exogenous ABHD11. HeLa cells expressing Cas9 were transduced with sgRNA targeting ABHD11 as described. Targeted cells were also transduced with exogenous ABHD11 with the PAM site mutated. Cells depleted of PHD2 served as a control for ABHD11 reconstitution. **h**–**i** Quantitative PCR (qPCR) of the HIF-1α target genes (VEGF and CAIX) in HeLa cells following ABHD11 depletion by sgRNA (*n* = 3, SEM, *p < 0.028, **p < 0.0065, two-tailed one-sample t test of ratio). sgRNA targeting OGDH and VHL were used as control for HIF-1α activation. **j** Genomic DNA was extracted from Hela control or mixed KO populations of ABHD11, LIAS or VHL, and 5hmC levels measured by immunoblot relative to total DNA content (Supplementary Fig. 1j). 5hmC levels were quantified using ImageJ. *n* = 3, Mean ± SEM **p = 0.010; ns:*p* = 0.39, VHL compared to control; two-tailed t test (not adjusted for multiple comparison). *Ct* = control.

Two genome-wide CRISPR sgRNA libraries were used to identify genes that when mutated activated the HIF reporter: the Brunello human genome-wide library (containing 76,441 sgRNA)^18^, and the Toronto genome-wide knockout library (containing 176,500 sgRNA)^19^. HeLa cells stably expressing the HRE-GFP^ODD^ reporter and Cas9 were transduced with each genome-wide library and iteratively sorted for GFP^HIGH^ cells by fluorescence-activated cell sorting (FACS) at day 10 and day 18 (Fig. 1a). SgRNAs enriched by FACS were identified by Illumina HiSeq and compared to a population of mutagenized cells that had not undergone phenotypic selection (Fig. 1a, b) (Supplementary Dataset 1). All screens were conducted in aerobic conditions (21% oxygen), thereby preventing oxygen availability limiting PHD function.

Both screens identified genes involved in the canonical pathway for HIF stability (*VHL, EGLN1* (*PHD2*)) and 2-OG metabolism (OGDHc components, lipoic acid synthesis pathway), validating the approach (Fig. 1b, c). Other biological processes that were significantly enriched for sgRNA included intracellular iron metabolism, the mTOR pathway, and transcriptional regulation (Fig. 1b, c). The reliance of the HIF pathway on these processes is well substantiated and in line with our prior studies using gene-trap mutagenesis in haploid cells^4,15^. In addition to these known pathways, we identified an uncharacterised α/β hydrolase, *ABHD11*, that was highly enriched for sgRNA in both screens (Fig. 1b, c).

CRISPR/Cas9 mixed knockout populations of ABHD11 in HeLa HRE-GFP^ODD^ cells, using three different sgRNA, validated the findings from the screens (Fig. 1d, Supplementary Fig. 1e). Stabilisation of HIF-1α was observed in multiple cell types (Fig. 1e, f, Supplementary Fig. 1f) and complementation of ABHD11 mixed knockout populations with overexpressed ABHD11 restored HIF-1α levels (Fig. 1g), confirming that ABHD11 loss resulted in HIF-1α accumulation.

We next asked if ABHD11 loss resulted in HIF-1α stabilisation through impaired 2-OG dependent dioxygenase activity. PHD function can be readily assessed by measuring HIF-1α prolyl hydroxylation using a HIF prolyl hydroxy-specific antibody. Mixed knockout populations of ABHD11 stabilised HIF-1α in a non-hydroxylated form, similar to the HIF-1α stabilisation with DMOG (Supplementary Fig. 1g). In contrast, inhibition of the VHL E3 ligase with VH298^20^, which stabilises HIF-1α by preventing ubiquitination and proteasome-mediated degradation showed high levels of hydroxylated HIF-1α (Supplementary Fig. 1g). To verify that the decreased prolyl hydroxylation was due to impaired PHD activity, we directly measured prolyl hydroxylation of a recombinant HIF-1α protein in control or ABHD11 deficient lysates^4^. Rapid prolyl hydroxylation was observed with a HeLa control lysate but this was markedly reduced in the ABHD11 depleted cells, similarly to loss of OGDHc function^4^ (Supplementary Fig. 1h, i). This PHD inhibition activated a transcriptional HIF response, promoting activation of HIF-1α target genes, VEGF and carbonic anhydrase 9, similarly to loss of VHL or OGDH (Fig. 1h, i).

We also explored whether ABHD11 loss altered the activity of other 2-OG dependent dioxygenases involved in transcription. ABHD11 KO cells showed a marked decrease in total DNA 5-hydroxymethylcytosine (5hmC) levels, similar to those observed when OGDHc function is impaired^4^ (Fig. 1j, Supplementary Fig. 1j), indicating that Ten-eleven translocation (TET) activity was impaired. However, the steady state levels of selected histone marks were not altered by ABHD11 depletion (Supplementary Fig. 1k). As levels of methylation depend on transferase activity, demethylation and nucleosome turnover, lysine demethylases (KDM) may still be affected by ABHD11 loss. Despite these differences between TET and KDM activity, these studies suggested that ABHD11 loss had broader implications for 2-OG dependent dioxygenase function, aside from PHDs.

### ABHD11 is required for OGDHc function

**Fig. 2:**
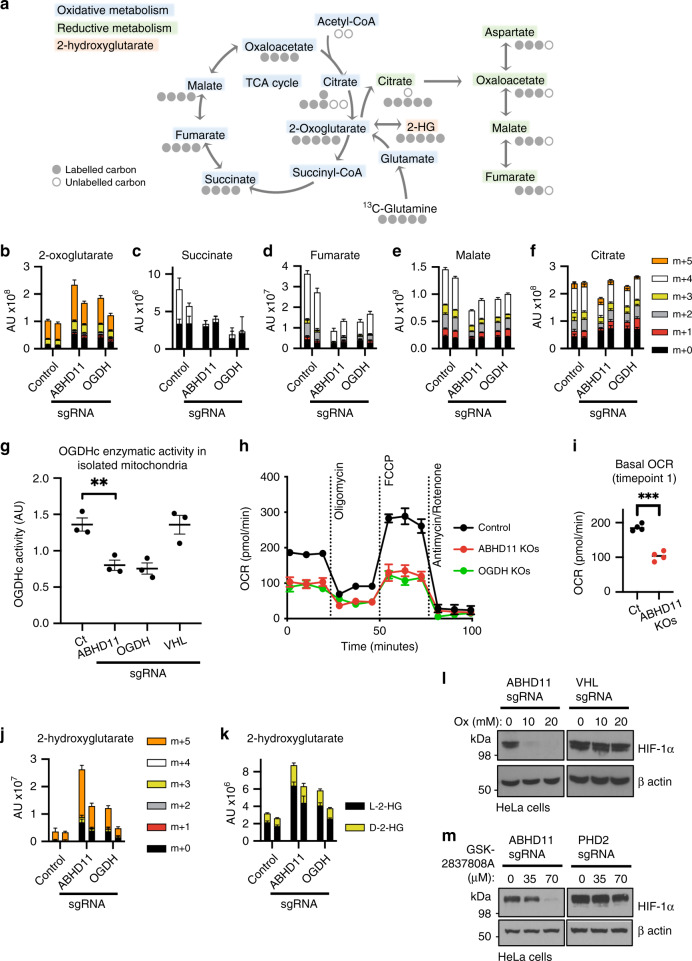
ABHD11 is required for OGDHc function. **a** Schematic of the TCA cycle (oxidative metabolism) and reductive carboxylation (reductive metabolism), illustrating the fate of ^13^C carbons upon incubation with [U-^13^C_5_]-glutamine. **b**–**g** Stable isotope tracing of control HeLa cells compared to mixed CRISPR KO populations (sgRNA) of ABHD11 or OGDH incubated with [U-^13^C_5_] glutamine. 2-oxoglutarate (**b**), succinate (**c**), fumarate (**d**), malate (**e**) and citrate (**f**), divided by metabolite isotopologues (*m* + 0 to *m* + 5) are indicated. Two biologically independent replicates, *n* = 5 technical replicates per sample, mean ± SD (**g**) OGDHc activity in isolated mitochondria. Mitochondria were extracted from control or mixed CRISPR KO populations of ABHD11, OGDH or VHL HeLa cells and OGDHc activity measured by a redox sensitive colorimetric probe for 2-OG oxidation. *n* = 3 biologically independent samples, mean ± SEM, **p = 0.0082, two-tailed *t* test. **h** Bioenergetic assays of oxygen consumption rates (OCR) in control, ABHD11 deficient or OGDH deficient HeLa cells (mixed KO populations). ABHD11 and OGDH were depleted as described, and analysed by using a Seahorse XF^e^24 Extracellular Flux Analyzer (*n* = 4 technical replicates per sample, mean ± SD). Three basal measurements were made at 9 min intervals followed by three measurements per treatment (1 μM oligomycin, 1 μM FCCP and 1 μM antimycin/rotenone). OCR was normalised to total cell number. **i** Comparison technical repeats at first basal measurement from (**h**); ***p = 6.3 × 10^−5^, two-tailed *t* test. **j** Measurement of 2-hydroxyglutarate (2-HG) levels following [U-^13^C_5_] glutamine stable isotope tracing in control HeLa cells compared to mixed CRISPR KO populations (sgRNA) of ABHD11 or OGDH. Metabolite isotopologues (*m* + 0 to *m* + 5) are indicated. Two biologically independent samples are shown; *n* = 5 technical replicates per sample, mean ± SD. **k** Relative quantification of 2-HG enantiomers upon derivatisation with diacetyl-L-tartaric anhydride and LC-MS analysis. **l**, **m** Inhibition of lactate dehydrogenase A (LDHA) in ABHD11 deficient HeLa cells. Mixed CRISPR KO ABHD11, VHL or PHD2 cells were treated with sodium oxamate (Ox) (**l**) or GSK-2837808A (**m**) as indicated for 24 h. HIF-1α levels were measured by immunoblot.

Impaired 2-OG dependent dioxygenase activity under aerobic conditions suggested that ABHD11 may be involved in 2-OG metabolism. Therefore, we first examined the consequences of ABHD11 loss on 2-OG levels and other TCA cycle intermediates. HeLa cells were depleted of ABHD11 and small molecule metabolites traced by incubating cells with uniformly ^13^C labelled ([U-^13^C_5_]) glutamine, followed by liquid chromatography mass spectrometry (LC-MS) (Fig. 2a). Cells deficient in OGDH were used as a control to measure perturbations of 2-OG metabolism. ABHD11 depletion resulted in 2-OG accumulation, similarly to OGDH loss (Fig. 2b). This increase in 2-OG was not due to activation of the HIF response, as we previously demonstrated that PHD2 deficiency does not perturb 2-OG levels^4^. ^13^C tracing confirmed that ABHD11 depletion impaired OGDHc function, as TCA cycle metabolites downstream of the OGDHc were decreased (succinate, fumarate and malate) (Fig. 2c–e), and cells adapted by showing a shift from oxidative metabolism to reductive carboxylation^4,21^, with a relative decrease in *m* + 4 and *m* + 2 citrate, and an increase in *m* + 5 and *m* + 3 citrate isotopologues (Fig. 2f).

To substantiate that ABHD11 levels altered OGDHc function, we measured OGDHc enzymatic activity in isolated mitochondria, using a colorimetric assay which detects oxidation of exogenous 2-OG with a redox sensitive probe (Fig. 2g). OGDHc activity was decreased in ABHD11 deficient mitochondria, similarly to levels observed with depletion of the OGDH subunit (Fig. 2g). Loss of OGDHc function was not due to HIF stabilisation, as VHL depletion had no effect on OGDHc activity (Fig. 2g). Bioenergetic profiling also showed that ABHD11 depletion impaired oxygen consumption rates (Fig. 2h, i), consistent with a major defect in the TCA cycle and oxidative phosphorylation.

2-OG accumulation can impair 2-OG dependent dioxygenase activity through the formation of L-2-HG (Fig. 2a)^4,6,7,9^. Consistent with this, we observed an accumulation in 2-HG levels following ABHD11 depletion, similarly to OGDH loss (Fig. 2j). 2-HG predominantly accumulated in its L enantiomeric form, although a small increase in D-2-HG was also observed (Fig. 2k). Stable isotope tracing with [U-^13^C_5_]glutamine confirmed L-2-HG was derived directly from 2-OG in both the ABHD11 and OGDH deficient cells (*m* + 5 isotopologues) (Fig. 2k).

Three enzymes are implicated in the formation of L-2-HG from 2-OG: lactate dehydrogenase A (LDHA), malate dehydrogenase 1 and malate dehydrogenase 2^6,8,9^. Reductive carboxylation and an acidic environment potentiate the reduction of 2-OG to L-2-HG and inhibition of LDHA alone is sufficient to prevent L-2-HG formation^4,7,8^. Therefore, to confirm that L-2-HG was responsible for decreased 2-OG dependent dioxygenase activity, we treated cells with sodium oxamate, which inhibits LDHA as well as decreasing 2-OG formation from glutamine^4,7^, or the selective LDHA inhibitor GSK-2837808A, and measured HIF-1α levels by immunoblot (Fig. 2l, m). Both treatments restored HIF-1α turnover in ABHD11 deficient HeLa cells. Together, these experiments confirmed that impaired OGDHc function and L-2-HG accumulation was responsible for the decreased PHD activity and activation of the HIF response.

### ABHD11 is a mitochondrial hydrolase

**Fig. 3:**
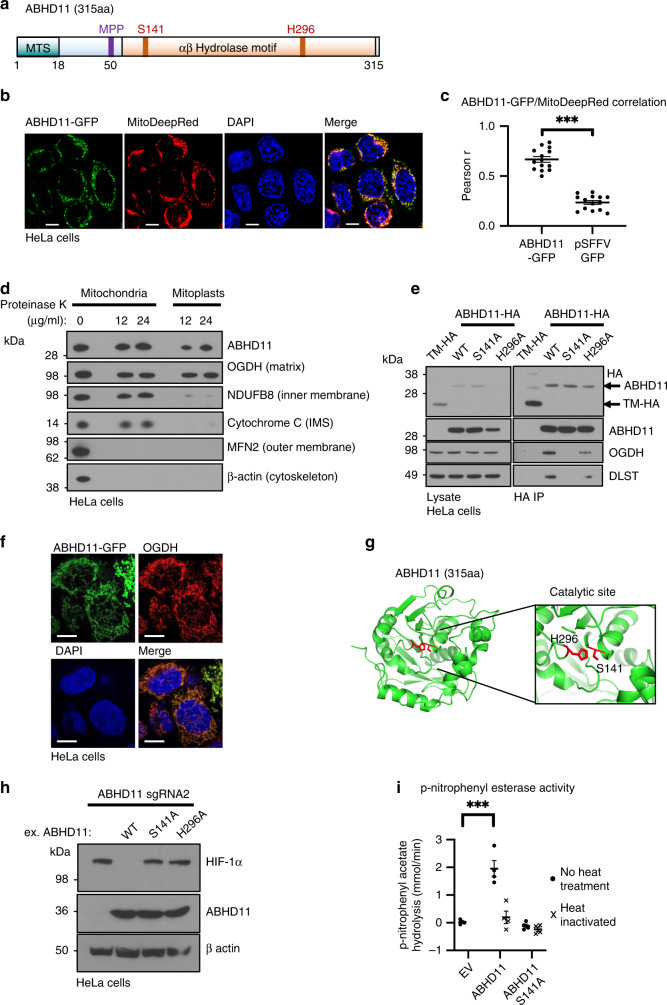
ABHD11 is a serine hydrolase that associates with the OGDHc. **a** Schematic of ABHD11 with the putative mitochondrial targeting sequence (MTS), mitochondrial processing peptidase (MPP) cleavage site and catalytic residues indicated (Serine 141, and Histidine 296). Modelled from UniProtKB—Q8NFV4, and MitoFates prediction tool^55^. **b** Confocal micrograph of HeLa cells lentivirally transduced with ABHD11-GFP. Mitochondria were visualised with MitoTracker Deep Red FM (MitoDeepRed); Scale = 10 μm, representative example from two biologically independent experiments and 14 images (**c**) Pearson correlation coefficient (Pearson *r*) comparing colocalisation of GFP and MitoTracker in HeLa cells expressing ABHD11-GFP (*n* = 14). HeLa cells expressing GFP under an SFFV promoter without a localisation signal served as a cytosolic control (pSFFV-GFP; *n* = 14) ***p = 7.0 × 10^−6^, two-tailed Mann–Whitney *U* test. **d** Mitochondrial protease protection assay. Mitochondria were extracted using the Qproteome Mitochondria Isolation Kit (Qiagen). Proteinase K was added to the final concentrations indicated, and incubation at 37 °C for 30 min. **e** Immunoprecipitation of ABHD11-HA with endogenous OGDHc components. ABHD11-HA or the inactive mutants (S141A and H296A) were transduced into HeLa cells, lysed and immunoprecipitated using the HA tag. TMEM199, a membrane bound protein tagged with HA (TM-HA) was used as a control. **f** Colocalisation of ABHD11 with the mitochondrial matrix protein, OGDH. HeLa cells expressing ABHD11-GFP were fixed in paraformaldehyde. ABHD11 and OGDH subcellular localisation was visualised by immunofluorescence confocal microscopy. Scale = 10 μm, representative image of five technical repeats. **g** In silico modelling of ABHD11 with putative catalytic site and key residues S141 and H296 (Phyre2 structural prediction against a template of murine epoxide hydrolase, PDB: 1cr6, and visualised using PyMOL 2.3). **h** Reconstitution of mixed KO population of ABHD11 with exogenous ABHD11, or enzymatic inactive mutants. **i** p-nitrophenyl esterase activity of purified ABHD11-FLAG. Purified wildtype or S141A ABHD11-FLAG were incubated with p-nitrophenyl acetate and hydrolysis measured by rate of increase in absorbance at 405 nm (37 °C for 40 min). An empty FLAG vector (EV), that had undergone affinity purification, was used as a control. ABHD11 enzymatic activity was also measured following heat inactivation of the protein (90 °C for 5 min). *n* = 4, Mean ± SEM, ***p = 0.0006, two-tailed *t* test.

**Fig. 4:**
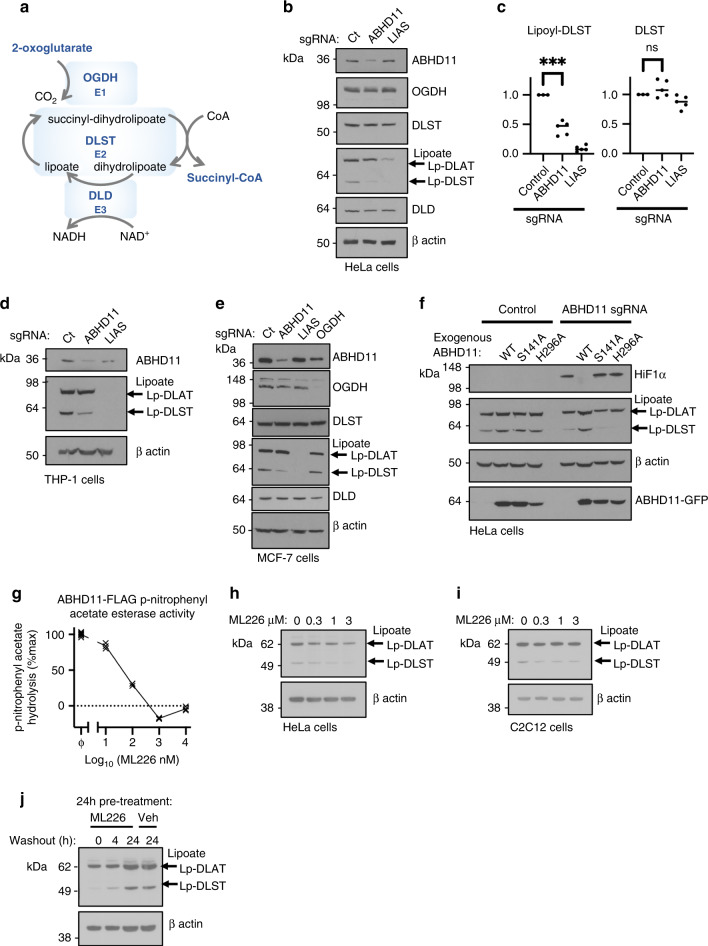
ABHD11 loss impairs lipoylation of the OGDHc. **a** OGDHc compromises 3 subunits: OGDH (E1) catalyses the oxidation of 2-OG to form the succinyl moiety, releasing carbon dioxide (CO_2_) and reducing the lipoate to form a succinyl-dihydrolipoate intermediate. DLST (E2) catalyses transfer of the succinyl moiety to Coenzyme A (CoA), forming succinyl CoA and releasing dihydrolipoylated DLST. Dihydrolipoate is oxidised by DLD (E3) to reform lipoate, with the free electrons reducing NAD^+^ to NADH. Thus 2-OG oxidation is coupled to cyclical lipoate reduction/oxidation and NADH formation. **b**, **c** Immunoblot and quantification of OGDHc subunits and lipoylation in ABHD11 or LIAS deficient cells. HeLa cells were transduced with sgRNA targeting ABHD11 or LIAS to generate mixed KO populations, and probed for OGDHc components (**b**). Lipoate (Lp) antibody detects lipoylated proteins. The predominant lipoylated proteins, DLAT and DLST, are indicated. ImageJ quantification of lipoyl-DLST (left) and DLST (right), *n* = 5 biologically independent samples (**c**). Mean ± SEM, ***p = 0.0004, ns:*p* = 0.19, two-tailed one sample *t* test. *Ct* = control (**d**, **e**) Immunoblot OGDHc subunits and lipoylation in ABHD11 or LIAS deficient THP-1 or MCF-7 cells. THP-1 (**d**) or MCF-7 (**e**) cells were transduced with sgRNA targeting ABHD11 or LIAS to generate mixed KO populations, and probed for OGDHc components or lipoate. β-actin served as a loading control. **f** Reconstitution of mixed KO population of ABHD11 with exogenous ABHD11-GFP, or enzymatic inactive mutants. **g** ML226 treatment in vitro. Purified wildtype ABHD11-FLAG was incubated with p-nitrophenyl acetate and hydrolysis measured by rate of increase in absorbance at 405 nm (37 °C for 30 min), with addition of ML226 at the indicated concentration. Hydrolysis activity was subtracted from a background control without ABHD11-FLAG, and is normalised to the activity of ABHD11-FLAG with vehicle control. *ϕ* = vehicle control; *n* = 3. **h**, **i** ML266 treatment in HeLa cells (**h**) and C2C12 myoblasts (**i**). Cells were treated for 24 h with ML226 at the indicated concentrations and lipoylation measured by immunoblot. **j** HeLa cells were treated with 1μM ML226, or 0.075% DMSO as a vehicle (veh) control. ML226 was washed out after 24 h and lipoylation recovery measured by immunoblot.

ABHD11 is a member of the alpha-beta hydrolase family, which contains 19 known genes, and encodes an α/β hydrolase fold (Supplementary Fig. 2a), typical of many proteases and lipases^22^. Unlike most alpha-beta hydrolase family members, ABHD11 is predicted to localise to the mitochondria through a classical mitochondrial targeting sequence (Fig. 3a, Supplementary Fig. 2a). Therefore, we used immunofluorescence microscopy to determine whether ABHD11 resided within mitochondria. Endogenous ABHD11 could not be readily detected by immunofluorescence, but exogenously expressed ABHD11 fused to GFP (ABHD11-GFP), which still retained function (see Fig. 4f), colocalised with MitoTracker DeepRed (Fig. 3b, c).

We biochemically confirmed ABHD11’s endogenous localisation using isolated mitochondria and a Proteinase K protection assay. Cytoskeletal and outer membrane proteins were rapidly lost with the addition of Proteinase K (30 min at 37 °C), but ABHD11 levels were unaffected, suggesting localisation inside of the outer membrane (Fig. 3d). Furthermore, ABHD11 was still retained in mitoplasts, irrespective of proteinase K treatment, consistent with its localisation to the mitochondrial matrix (Fig. 3d).

As the stable isotope tracing demonstrated that ABHD11 loss altered OGDHc activity, we determined if ABHD11 associated with components of the complex. Both OGDH and DLST immunoprecipitated with HA conjugated ABHD11 (Fig. 3e), and ABHD11-GFP colocalised with the OGDH (Fig. 3f). We also subjected immunoprecipitated ABHD11-HA to mass spectrometry, which confirmed the association with OGDH and DLST (Supplementary Table 1). Furthermore, our findings were consistent with a prior unbiased mass spectrometry analysis of interactions between mitochondrial proteins, which identified that ABHD11 associated with OGDH with high confidence^23^.

We next examined if ABHD11 enzymatic activity was required for its effect on HIF-1α stability. Structural modelling of ABHD11 predicted a typical α/β hydrolase fold with two catalytic motifs (Fig. 3a, g). Hydrolase activity is predicted to arise from the serine nucleophile motif (GXSXG), but ABHD11 also encodes a putative acyltransferase motif (HXXXXD) found in several other α/β hydrolases^22^ (Fig. 3a, g, Supplementary Fig. 2b). Reconstituting ABHD11 deficient cells with the putative ABHD11 nucleophile mutant (S141A) or acyltransferase mutant (H296A) did not restore HIF-1α to basal levels, suggesting that ABHD11’s hydrolase activity was required (Fig. 3h). Importantly, this was not due to mis-localisation of the ABHD11 mutants, as both ABHD11 S141A and ABHD11 H296A were visualised within mitochondria (Supplementary Fig. 3a, b).

To confirm that these mutations were altering ABHD11 enzymatic activity, we purified wildtype and S141A ABHD11 and measured hydrolysis of p-nitrophenyl ester, a substrate validated for generic α/β hydrolase activity^22,24^. Wildtype ABHD11 protein and the S141A mutant were isolated by expression in HEK293T cells and FLAG tag affinity purification (Supplementary Fig. 4). ABHD11 predominantly migrated as a single species but a slower migrating form was apparent in the cell extract and purified protein, consistent with an immature form prior to mitochondrial insertion (Supplementary Fig. 4a). Mass spectrometry analysis confirmed ABHD11’s identity and demonstrated that the mitochondrial targeting sequence was lost in the predominantly expressed form (Supplementary Fig. 4b) (the slower migrating species was of too low abundance). Size exclusion chromatography identified two peaks but full length ABHD11 was only detected in the second peak, at an elution volume consistent with a monomeric species (Supplementary Fig. 4c, d). Hydrolysis of the p-nitrophenyl ester confirmed ABHD11 enzymatic activity, but this was lost with the S141A mutant and following heat treatment (Fig. 3i). Thus, ABHD11 is a mitochondrial hydrolase that associates with the OGDHc, and loss of its enzymatic activity leads to HIF-1α accumulation.

### ABHD11 loss impairs lipoylation of the OGDHc

Conversion of 2-OG to succinyl-CoA by the OGDHc requires decarboxylation and the formation of succinyl intermediate (succinyl-dihydrolipoate), dependent on the cyclical reduction and oxidation of the lipoylated DLST subunit (Fig. 4a). Therefore, to understand how ABHD11 is required for OGDHc function, we first examined whether protein levels of core OGDHc components or its lipoylation were altered. ABHD11 depletion did not alter total levels of the OGDHc subunits (OGDH, DLST or DLD) in HeLa cells (Fig. 4a, b). However, using a specific anti-lipoate antibody that detects conjugated lipoamide, we observed a reproducible loss of the faster migrating lipoylated protein species, attributed to the lipoylated DLST subunit of the OGDHc (Fig. 4b, c). Immunoprecipitation of endogenous DLST confirmed loss of lipoylation following ABHD11 depletion, without altering total DLST levels (Supplementary Fig. 5a), and this decreased DLST lipoylation was observed in several cell types (Fig. 4d, e). Furthermore, in contrast to complete disruption of lipoic acid synthesis by LIAS depletion, ABHD11 loss preferentially decreased DLST lipoylation, without altering the other abundantly lipoylated protein within the mitochondria, the DLAT (dihydrolipoamide acetyltransferase) subunit of the pyruvate dehydrogenase complex (PDHc) (Fig. 4b–e, Supplementary Fig. 5b). Indeed, PDHc function, as measured by [U-^13^C_6_] glucose stable isotope tracing, was not impaired in the ABHD11 deficient HeLa cells (Supplementary Fig. 6a–g), and lactate production was not increased compared to control HeLa cells (Supplementary Fig. 6h).

Complementation studies were used to determine whether the enzymatic activity of ABHD11 was required for lipoylation of the OGDHc. Exogenous wildtype or mutant ABHD11 were expressed in mixed ABHD11 KO populations and lipoylation levels measured by immunoblot. DLST lipoylation was restored with the wildtype ABHD11 but not with the S141A or H296A mutants (Fig. 4f). HIF-1α levels were only reduced to basal levels by reconstituting with wildtype ABHD11 but not the nucleophile mutants (Fig. 4f), as previously shown.

Lastly, to confirm the enzymatic requirement of ABHD11 in OGDHc lipoylation, we used a highly selective covalent inhibitor of ABHD11, ML226, that was initially developed as a tool for screening serine hydrolases^25^. ML226 treatment inhibited ABHD11 p-nitrophenyl ester hydrolysis in vitro (Fig. 4g) and decreased OGDHc lipoylation in HeLa cells (Fig. 4h) and cultured myoblasts (C2C12) (Fig. 4i). In addition, DLST lipoylation recovered after ML226 washout in cells, indicating OGDHc lipoylation can be restored (Fig. 4j). Thus, ABHD11 hydrolase activity is required for OGDHc lipoylation.

### ABHD11 maintains functional lipoylation of DLST

The finding that ABHD11 loss showed a selective loss of DLST lipoylation was unexpected, as prior genetic studies of lipoate conjugation had not shown a requirement for an additional enzyme^26^. Furthermore, we confirmed that ABHD11 loss differed to depletion of other components of the lipoic acid synthesis pathway by generating CRISPR/Cas9 mixed KO populations of the key enzymes involved (Supplementary Fig. 7a, b). Lipoyl(octanoyl) transferase 2 (LIPT2), LIAS, and lipoyltransferase 1 (LIPT1) all reduced DLAT and DLST lipoylation in HeLa cells to a similar level, but only ABHD11 showed a selective loss of DLST lipoylation (Supplementary Fig. 7b). This preferential decrease in DLST lipoylation following ABHD11 loss argued against a general role for ABHD11 in lipoyl synthesis, and while it remained possible that ABHD11 was required for the final catalysis of DLST lipoylation, prior genetic studies suggested that LIPT1 was sufficient for this step^26–28^.

Rather than acting as a conjugating enzyme, we hypothesized that ABHD11 may directly or indirectly be involved in maintaining a functional lipoate moiety on the OGDHc complex. We observed that ABHD11 loss in HeLa cells led to decreased cell viability after prolonged passage for three weeks. However, it was unlikely that general growth inhibition was responsible for the lipoylation phenotype as ML226 treatment, which efficiently inhibited ABHD11, did not alter cell growth (Supplementary Fig. 8a). We also examined whether ABHD11 activity altered the mitochondrial redox environment, which could influence the reduction and oxidation of lipoylated DLST. Stable isotope tracing showed no overall change in cellular glutathione (GSH) levels (Supplementary Fig. 8b). Small changes in mitochondrial ROS were observed with ABHD11 loss, using MitoSOX Red, similarly to OGDH or LIAS depletion (Supplementary Fig. 8c, d). However, ML226 treatment, showed no change MitoSOX Red levels, and importantly, Antimycin A, which increased mitochondrial ROS to higher levels than ABHD11 inhibition, was not sufficient to activate the HIF reporter (Supplementary Fig. 8e). Thus, alterations in mitochondrial ROS were unlikely to account for the HIF stabilisation or altered lipoylation following ABHD11 loss or inactivation of the OGDHc.

**Fig. 5:**
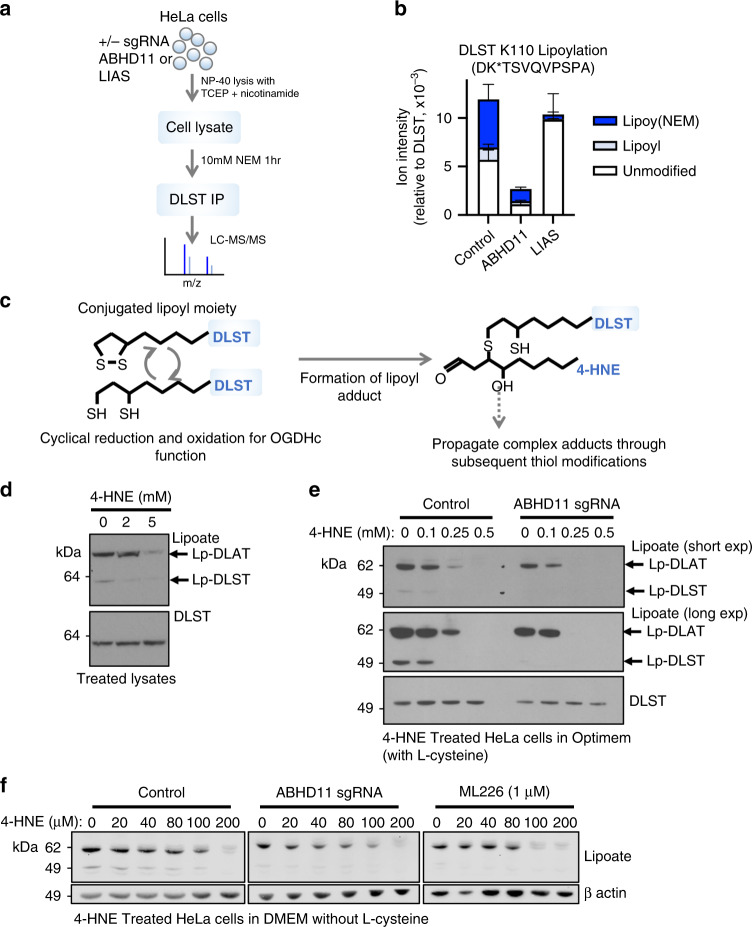
ABHD11 prevents the formation of lipoyl adducts by lipid peroxidation products. **a**, **b** Mass spectrometry analysis of DLST lipoylation. DLST was immunoprecipitated from HeLa control, ABHD11 deficient or LIAS deficient cells, and treated with NEM to modify the free thiols and maintain the lipoyl moiety in a reduced state. After SDS-PAGE, protein samples were digested with Asp-N protease and analysed by LC-MS/MS. **b** Normalised level of lipoylated DLST peptide compared to DLST reference peptide. Relative levels of the unmodified, NEM-dihydrolipoamide, and lipoamide DLST peptide are shown. *n* = 3, Mean ± SEM. **c** Schematic of normal cyclical reduction and oxidation of the lipoyl moiety on DLST (left). The reduced dihydrolipoamide can react with lipid peroxidation products (e.g., 4-HNE) to form lipoyl adducts through the free thiols, which may also propagate (right). **d**–**f** Effect of 4-HNE treatment on lipoylation. HeLa cells lysates were treated with 4-HNE at the indicated concentrations for 60 min (50 °C) and immunoblotted for lipoylated proteins or total DLST (**d**). Control or ABHD11 deficient HeLa cells were treated with 4-HNE in serum-free Optimem (**e**) or serum and L-cysteine free DMEM (**f**) for 90 min (37 °C), lysed and immunoblotted for lipoylation and total DLST. .

To explore further how ABHD11 activity altered DLST lipoylation we used mass spectrometry analysis of the lipoate moiety. Immunoprecipitated DLST was treated with a reducing agent and then incubated with N-ethylmaleimide (NEM), forming an NEM-lipoyl conjugate, which had previously been shown to aid detection of the lipoate moiety^29^ (Fig. 5a, Supplementary Fig. 9a). Interestingly, NEM treatment prevented detection of immunoprecipitated lipoylated DLST by immunoblot (Supplementary Fig. 9a), demonstrating that the anti-lipoate antibody only detected the functional lipoate and not the NEM-modified form, suggesting that the apparent loss of DLST lipoylation in ABHD11 deficient cells may be due to modification of the lipoate moiety. We next measured levels of DLST lipoylation (NEM-lipoyl) by label-free quantification on immunoprecipitated DLST from wildtype HeLa cells or those deficient in LIAS or ABHD11 (Fig. 5a, b, Supplementary Fig. 9b). To account for potential differences in DLST protein abundance around the lipoylated region (DK*TSVQVPSPA), we normalised these peptides to the sum of all DLST peptide label-free quantification values. Approximately 50% of the DK*TSVQVPSPA DLST peptide in wildtype cells was modified with lipoate compared to the unmodified form and as expected, nearly all the lipoate detected was modified with NEM (Fig. 5b). DLST lipoylation was nearly completely lost in the LIAS deficient cells, with the majority of the DLST lipoylated peptide region found to be unmodified (Fig. 5b), confirming that this approach could readily identify a defect in lipoyl synthesis and conjugation. However, ABHD11 deficiency did not result in an accumulation of the unmodified DK*TSVQVPSPA peptide, which could have been expected with a defect in conjugation. Instead, both the unconjugated or NEM-lipoyl DK*TSVQVPSPA peptide were barely detectable, with a 10-fold decrease in abundance compared to the control or LIAS null cells (Fig. 5b). This decrease was not due to less total DLST, as DK*TSVQVPSPA levels were normalised to other DLST peptides upstream or downstream of the lipoylated region. Therefore, a modification of the DK*TSVQVPSPA peptide of undefined mass accounted for the apparent decrease in peptide abundance. Common post-translational modifications (e.g., ubiquitination, phosphorylation or acetylation), combinations of modifications, or known DLST intermediates (e.g., succinyl-dihydrolipoamide, acyl-dihydrolipoamide or S-glutathionylation) (Supplementary Dataset 2) did not account for the peptide loss of the lipoylated DLST region, suggesting the formation of lipoyl adducts that were not detectable by mass spectrometry.

The thiols within the lipoamide moiety are sensitive to attack by lipid peroxidation products, which disrupt OGDHc catalysis by preventing the cyclical oxidation and reduction of the lipoyl conjugate^30,31^ (Fig. 5c). We measured whether common lipid peroxidation products formed in cells (4-hydroxy-2-nonenal (4-HNE) or 4-oxononenal (4-ONE))^30–32^ modified the lipoate moiety on immunoprecipitated DLST, but these modifications did not account for the unassigned mass of the DLST peptide (Supplementary Dataset 2). However, the complex nature of lipid based adducts of undefined and variable lengths may preclude their detection.

While the exact nature of the lipoyl adduct formed in ABHD11 deficient cells was unclear, we examined whether exogenous treatment with 4-HNE could alter DLST lipoylation, similarly to ABHD11 depletion. 4-HNE treatment of cell lysates preferentially decreased detection of DLST lipoylation by immunoblot (Fig. 5d), consistent with the formation of lipoyl adducts preventing binding to the antibody. DLAT lipoylation was only affected at high concentrations (5 mM) of 4-HNE (Fig. 5d), suggesting that DLAT may be more resistant to lipoyl adduct formation than DLST, and consistent with our findings that ABHD11 loss preferentially effects the OGDHc.

**Fig. 6:**
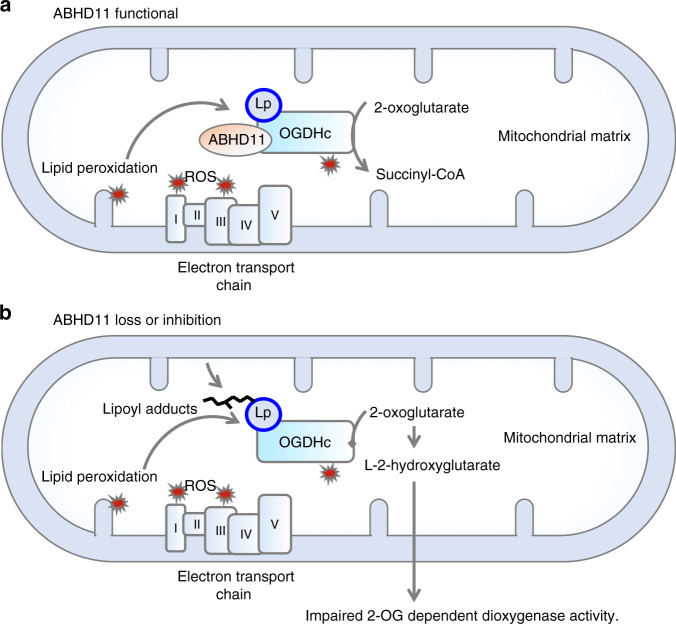
ABHD11 regulation of OGDHc activity. Working model for functional lipoylation of the OGDHc. **a** Cyclical reduction and oxidation of lipoate is required for OGDHc catalytic activity, and this process requires ABHD11 which associates with the OGDHc, maintaining functional lipoylation and allowing the conversion of 2-OG to succinyl CoA. **b** When ABHD11 is depleted or inhibited, lipoyl adducts form, preventing functional lipoylation. These adducts may arise from lipid peroxidation products (e.g. 4-HNE) formed by ROS generated by the OGDHc or by the electron transport chain, or by other as yet undefined mechanisms. Loss of functional lipoylation leads to 2-OG accumulation and the formation of L-2-HG, which can inhibit 2-OG dependent dioxygenases involved in the HIF response and DNA hydroxymethylation.

Finally, to explore whether ABHD11 protected against to the formation lipoyl adducts, such as those formed by 4-HNE, we measured if ABHD11 loss or inhibition made the OGDHc more susceptible to lipid peroxidation damage. Control or ABHD11 depleted HeLa cells or lysates were treated with 4-HNE, and lipoylation detected by immunoblot. 4-HNE decreased the detection of DLST preferentially to DLAT within cell lysates (Fig. 5d), consistent with lipoyl adducts preventing detection of the lipoyl moiety by immunoblot. 4-HNE treatment of cells also decreased DLST functional lipoylation preferentially to DLAT, and ABHD11 deficient cells were more susceptible to 4-HNE treatment compared to the control cells (Fig. 5e). Overexpression of ABHD11 inactive mutants competed with endogenous ABHD11 to also show an increase lipoyl-adduct formation following 4-HNE treatment (Supplementary Fig. 9c). ABHD11 overexpression did not increase DLST lipoylation compared to control cells (Supplementary Fig. 9c) but this finding is consistent with exogenous ABHD11 not increasing total lipoylation levels and reflect that OGDHc lipoylation is tightly regulated, with only 50% of DLST modified by lipoylation. While these studies demonstrated that 4-HNE could disrupt functional lipoylation, we were concerned that the concentrations required were higher than prior reports^33,34^, and considered that this may be due to the presence of L-cysteine within media. Therefore, we repeated these assays using lower concentrations of 4-HNE in media without L-cysteine (Fig. 5f). We now observed impaired DLST lipoylation with low concentrations of 4-HNE (40 µM) in control HeLa cells, with complete loss of DLST in ABHD11 deficient cells at 20 µM 4-HNE (Fig. 5f). Furthermore, while 4-HNE preferentially altered DLST, DLAT lipoylation was also decreased in the ABHD11 null cells compared to the controls (Fig. 5f). Similar findings were observed with ABHD11 inhibition, consistent with a requirement for ABHD11 to maintain functional lipoylation in the context of lipid peroxidation products. In conclusion, while the nature of adducts formed on lipoylated DLST remain to be fully determined, these studies demonstrate that ABHD11 is required for functional lipoylation of the OGDHc, and may protect against the formation of lipoyl adducts, such as those formed by 4-HNE (Fig. 6).

## Discussion

This study identifies ABHD11 as a mitochondrial enzyme required for OGDHc function, and to our knowledge, is the first example of a mitochondrial pathway that maintains TCA cycle integrity by preserving functional OGDHc lipoylation (Fig. 6). Moreover, we demonstrate that ABHD11 inhibition allows 2-OG metabolism to be modulated in multiple cells and in a reversible manner, with potential broad implications for altering cell fate-decisions and manipulating 2-OG abundance in tumours.

The selective loss of lipoylated DLST following ABHD11 depletion initially suggested that it may be necessary for OGDHc lipoate conjugation. However, a requirement for ABHD11 in lipoate synthesis had not been previously observed^13,26,35^, and LIPT1 deletion or human loss of function mutations prevent PDHc and OGDHc lipoylation^26–28^. It was possible that ABHD11 transfers lipoate moieties between 2-oxoacid dehydrogenases, but our mass spectrometry findings argued against this. If ABHD11 was required for lipoate transfer, the unmodified DLST peptide should accumulate in ABHD11 depleted cells. Instead, we found an absence of both the modified and unmodified lipoylated region of DLST by mass spectrometry (Fig. 5b, c). Similar coverage of DLST peptides upstream and downstream of the lipoylated region confirmed that there was no change in total DLST levels following ABHD11 loss. Therefore, a peptide of undefined mass must account for the apparent loss of this region, indicating a post-translational modification other than lipoylation or the formation of a lipoyl adduct.

Common post-translational modifications (e.g. ubiquitination, phosphorylation or acetylation), combinations of modifications, or known DLST intermediates (e.g. succinyl-dihydrolipoamide, acyl-dihydrolipoamide or S-glutathionylation) (Supplementary Dataset 2) did not account for the peptide loss of the lipoylated DLST region, suggesting that this DLST peptide was not uniformly modified. Lipid peroxidation products (hydroxyalkenals, such as 4-HNE), arise from free radical propagation through phospholipids^31^, and can easily react with thiol groups, inactivating 2-oxoacid dehydrogenases by forming lipoyl adducts^30^. We did not observe 4-HNE lipoyl adducts on immunoprecipitated DLST, but the hydrophobicity and complex nature of these adducts^32^ may preclude their detection by mass spectrometry, accounting for the apparent loss of the DLST DK*TSVQVPSPA peptide that we observed (Fig. 5b). We also required high concentrations of 4-HNE to decrease detectable lipoylation in immunoprecipitated DLST, and 0.1 mM 4-HNE in cells incubated in serum-free media. High levels or prolonged treatment of 4HNE may lead to depletion of cellular antioxidant levels and apoptosis^36,37^, but we did not observe changes in DLAT lipoylation at 0.1 mM consistent with a selective effect on DLST, as previously observed^38^. Furthermore, treatment of HeLa cells with 4-HNE in media without L-cysteine resulted in selective loss of DLST lipoylation at lower, biologically relevant 4-HNE concentrations^33,34^.

The apparent specificity of ABHD11 for DLST may relate to the preponderance of lipoyl adducts formed by the OGDHc compared to other lipoylated proteins, or selectively binding to the OGDHc, rather than all 2-oxoacid dehydrogenases. It is possible that ABHD11 may regulate DLST indirectly, but this is unlikely to be due to altered mitochondrial ROS (Supplementary Fig. 8). The association with DLST and OGDH (Fig. 3e), and marked accumulation of 2-OG (Fig. 2) are also consistent with a direct role on OGDHc function. These findings are also supported by prior mass spectrometry interactome studies, showing an association of ABHD11 with OGDH^23^. Whether ABHD11 interacts with other 2-oxoacid dehydrogenases will be of interest to explore further. We observed that ABHD11 can interact with DLAT by mass spectrometry (Supplementary Table 1), and a small decrease in DLAT lipoylation following ABHD11 loss was observed. ABHD11 deficient cells were also more susceptible to impaired lipoylation following 4-HNE treatment. However, ABHD11 loss did not increase pyruvate levels in HeLa cells (Supplementary Fig. 5), which would be expected if PDHc activity was significantly impaired.

ABHD11 is one of a family of alpha-beta hydrolase domain-containing enzymes, which as a group are poorly characterised. Of these, only ABHD10 and ABHD11 are known to be mitochondrial^39^. ABHD10 is has recently been shown to be an acyl protein thioesterase, with S-depalmitoylase activity against the anti-oxidant protein peroxiredoxin-5^40^. These findings would be consistent with our observations for ABHD11 regulating the thiol-containing lipoate moiety on DLST. However, ABHD11 has less sequence identity with ABHD10 than other ABHD family members (Supplementary Fig. 2), and is not inhibited by ML226^25^.

This work provides insights into the functional role of ABHD11, for which no physiological substrate or role has been identified previously. ABHD11 is one of ~26 genes included in the 7q11.23 hemizygous deletion of Williams-Beuren syndrome, a rare multisystem disorder often characterised by developmental and cardiac abnormalities^41^. While the phenotype of cardiac and soft tissue disease is felt largely due to loss of Tropoelastin 1 (ELN1)^41^, a functional understanding of ABHD11 may offer some insights into aspects of this syndrome. It will also be of interest to explore further the biological consequences of ABHD11 loss compared to other lipoate enzymes. ABHD11 loss disrupts the TCA cycle, impairing oxidative phosphorylation and promoting reductive metabolism, but ABHD11 has a distinct metabolic phenotype compared to loss of other lipoic acid pathway enzymes. Germline mutations in LIAS, LIPT2 and LIPT1 result in impaired PDHc and OGDHc activity^13,27,42,43^, but ABHD11 loss did not significantly alter DLAT lipoylation in several cancer lines. PDHc activity is required for lipogenesis, by providing acetyl-CoA, and LIPT1 mutations result in defective lipid synthesis^44^. As ABHD11 acts predominantly on the OGDHc, it is possible that the preserved activity of PDHc fuels lipogenesis, and cell survival, and it will be important to determine whether ABHD11 activity, and the resulting HIF activation, feeds back on lipid synthesis and the mitochondrial fatty acid pathway.

This study and prior reports demonstrate that human mutations in lipoylation enzymes increase 2-OG levels and promote L-2-HG formation^4,42^. However, how L-2-HG inhibits 2-OG sensitive enzymes when 2-OG is abundant remains to be fully resolved. It is possible that L-2-HG may allosterically inhibit the enzymes, or that additional factors aside from L-2-HG result in decreased dioxygenase activity. The reasons for the propensity to form L-2-HG under certain conditions of 2-OG accumulation also remains to be determined, as in other cellular responses, such as embryonic stem cell pluripotency^2^, 2-OG treatment does not result in inhibition of 2-OG dependent dioxygenases. These discrepancies may relate to the reductive environment that occurs when OGDHc activity is impaired (which is likely to occur with ABHD11 loss), or the metabolic phenotype of the cell (e.g. reliance on oxidative phosphorylation versus glycolysis).

Lipoic acid has been traditionally described as an essential cofactor for 2-oxoacid dehydrogenases, but only 50% of DLST in HeLa cells was observed to be lipoylated in resting cells (Fig. 5b), and OGDHc lipoylation was rapidly stored after washout of ML226, suggesting a reserve capacity to alter lipoate levels and increase OGDHc activity (Fig. 4j). These findings show that lipoylation is a dynamic modification that must be maintained, which is further supported by recent observations that SIRT4 act as a lipoamidase, altering PDHc function^29^, and that increased lipoylation can enhance brown adipose tissue function, decreasing age-associated obesity^45^. Therefore, modulating lipoylation through ABHD11 activity provides an attractive approach to manipulating 2-OG metabolism. Moreover, these studies extend the role of lipoylation beyond an enzymatic cofactor, to a dynamic modification that couples the mitochondria to a transcriptional adaptive response mediated by 2-OG and oxygen sensitive enzymes.

## Methods

### Cell lines and reagents

HeLa, MCF-7, C2C12 and HEK293T cells were maintained in DMEM (Sigma D6429), and THP-1 cells maintained in RPMI-1640 (Sigma R8758), both supplemented with 10% foetal calf serum (Sigma P4333) and 100 units/ml penicillin with 100 µg/ml streptomycin, in a 5% CO_2_ incubator at 37 °C. HeLa HRE-GFP^ODD^ cells were produced by lentiviral transduction of the HRE-GFP^ODD^ reporter^4^. All cell lines were authenticated (Eurofins). Hypoxia treatments were performed at 1% oxygen, 5% CO_2_ in a H35 Hypoxystation (Don Whitley Scientific).

Full details of reagents and antibodies used are shown in Supplementary Table 2.

### Constructs

CRISPR sgRNAs were cloned into a lentiviral sgRNA expression vector pKLV-U6gRNA(BbsI)-PGKpuro2ABFP^46^. All sgRNA sequences are detailed in Supplementary Table 3.

ABHD11 constructs were generated from the I.M.A.G.E. cDNA clone (IRATp970F0688D, Source Bioscience), cloned into the pHRSIN pSFFV backbone with pGK-blasticidin resistance (a gift from Paul Lehner), using NEBuilder HiFi (NEB). Prior to assembly, silent mutations were introduced inside the sequence targeted by ABHD11 sgRNA 2, using PCR primers detailed in Supplementary Table 3. Mutations of catalytic residues serine 141 to alanine (S141A) and histidine 296 to alanine (H296A) of ABHD11 was created using NEBuilder HiFi, with primers detailed in Supplementary Table 3. Lentiviral expression vectors (pHRSIN) for ABHD11, S141A ABHD11 and H296A ABHD11 with C-terminal eGFP tags or HA tags were created using NEBuilder HiFi (NEB). ABHD11 was also cloned into a transfection vector, pCEFL 3xFLAG mCherry vector, encoding a C-terminal 3X FLAG tag and mCherry under a separate promoter (a gift from David Ron)^47^, using Gibson Assembly (NEB) and NEBuilder HiFi.

### CRISPR/Cas9 sgRNA pooled libraries

The Human Brunello library was a gift from David Root and John Doench^18^ (Addgene #73178). The Toronto human knockout pooled library (TKO) Version 1 (Addgene ♯1000000069) was a gift from Jason Moffat^19^.

### Preparation of lentivirus and transductions

HEK293T cells were transfected using TransIT-293 (Mirus) according to the manufacturer’s protocol. For small scale experiments, lentivirus was produced in 6-well plates containing 2 ml media, using 2 µg DNA (DNA was mixed in a 3:2:4 ratio of the relevant expression plasmid, pCMV-dR8.91 (gag/pol) and pMD.G (VSVG)^48^. Viral supernatant was harvested after 48 h, passed through a 0.45 µm filter, and frozen at −80 °C.

Cells were transduced with lentivirus by adding an appropriate volume of thawed viral supernatant. In the case of single-gene knockdown in HeLa cells, 250 µl of virus with 5 × 10^4^ cells in a 24-well plate made up to 1 ml media. For the screens, a titration of increasing volumes of virus was used, with 10^6^ HeLa cells in a 6-well plate. Cell plates were centrifuged for 1 h at 37 °C at 750 × *g* immediately after addition of virus.

### Whole-genome CRISPR/Cas9 forward genetic screens

HeLa HRE-GFP^ODD^ cells were transduced with Streptococcus pyogenes Cas9 (pHRSIN-FLAG-NLS-CAS9-NLS-pGK-Hygro)^49^ and selected for Cas9 expression using hygromycin. 5 × 10^7^ (Brunello) or 10^8^ (TKO) HeLa HRE-GFP^ODD^ cells were transduced with the appropriate volume of pooled sgRNA virus (multiplicity of infection (MOI) of ~0.3), maintaining at least 150-fold sgRNA coverage. After 30 h, cells were treated with puromycin 1 µg/ml for 5 days. Representation was maintained throughout the screen such that no selection event occurred where the library was cultured at fewer than 200 times the number of sgRNA sequences in the library. The library was pooled immediately before any selection event.

FACS was performed by harvesting 10^8^ cells, washing the cells in PBS, and then resuspending them in PBS containing 2% foetal calf serum and 10 mM HEPES (Sigma H0887). Cells were sorted using an Influx cell sorter (BD); GFP-high cells were chosen in a gate set at one log_10_ unit above the mode of the untreated population.

Genomic DNA was extracted using a Gentra Puregene Core kit (Qiagen). Lentiviral sgRNA inserts were amplified in a two-step PCR (with Illumina adapters added on the second PCR), as previously described^49^. For the TKO screen, the forward inner PCR and sequencing primers were modified (Supplemental Table 3).

Sequencing analysis was performed by first extracting the raw sequencing reads, trimming the first 20 bp (FASTX-toolkit), and aligning against the appropriate sgRNA library using Bowtie^50^. Read counts for each sgRNA were compared between conditions, and Benjamini-Hochberg false discovery rates for each gene calculated, using MAGeCK^51^ (Supplementary Dataset 1). The analysis presented compares DNA extracted following the second sort to an unsorted DNA library taken at the same timepoint.

### Flow cytometry

HeLa HRE-GPP^ODD^ cells were washed in PBS and fixed in PBS 1% formaldehyde prior to analysis. For MitoSOX Red staining HeLa cells were plated in 24-well plates. After 24 h cells were treated with or without 10 μM Antimycin A for 37 °C for 30 min, washed with PBS or Hank’s Balanced Salt Solution (HBSS), and then stained with 5μM MitoSOX Red in PBS or HBSS for 10 min at 37 °C. Cells were harvested and resuspended in PBS for analysis by flow cytometry (BD Fortessa; software: FACSDiva 8.0). Flow cytometry gating strategy is shown in Supplementary Fig. 10. Images were presented using FlowJo v10 (BD).

### Immunoblotting and immunoprecipitation

Cells were lysed in an SDS lysis buffer containing 2% SDS, 50 mM Tris pH 7.4, 150 mM NaCl, 1 mM dithiothreitol, 10% glycerol and 1:200 Benzonase nuclease (Sigma), for 15 min at room temperature, then heated at 90 °C for 5 min. Proteins were separated with SDS-PAGE electrophoresis, transferred to a PVDF membrane, and probed using appropriate primary antibodies and a secondary with HRP conjugate. Densitometry measurements were made using ImageJ^52^.

To identify protein interactions with ABHD11, HeLa cells lentivirally transduced with ABHD11 with a C-terminal HA tag were lysed in a buffer containing 100 mM Tris pH 8.0, 140 mM NaCl, 1% IGEPAL CA-630 (Sigma), 1 mM PMSF (Sigma P7626) and cOmplete Protease Inhibitor Cocktail (Roche). After centrifugation at 17,000 × *g*, the supernatant was pre-cleared using Sepharose CL-4B (GE Heathcare) and then incubated with EZView HA Red Anti-HA Affinity Gel (Sigma E6779) overnight on a rotator. Resins were washed with Tris-buffered saline containing 0.1% IGEPAL CA-630, and a further two washes with Tris-buffered saline. Proteins were eluted using an SDS lysis buffer (4% SDS, 100 mM Tris pH 7.4, 300 mM NaCl, 2 mM dithiothreitol, and 20% glycerol) heated at 90 °C for 5 min, and separated using SDS-PAGE.

### Confocal microscopy

Mitochondrial labelling was performed using MitoTracker Deep Red FM (Thermo M22426). Cells were cultured overnight on a 1 cm glass coverslip, incubated with 250 nM Mitotracker Deep Red FM for 40 min, and after washing with PBS fixed with 4% paraformaldehyde for 20 min. Cells were mounted to slides using ProLong Gold Antifade Mountant with DAPI (Thermo).

For immunofluorescence microscopy of OGDH, cells were cultured overnight on a 1 cm glass coverslip. After washing with PBS, cells were fixed with 4% paraformaldehyde, permeablised (0.3% Triton X-100, 3% bovine serum albumin) and stained for OGDH (primary antibody at 1:100 dilution for 30 min, Alexa Fluor secondary at 1:1000 (Thermo A21245)). Cells were mounted to slides using ProLong Gold Antifade Mountant with DAPI. Slides were imaged using an LSM880 confocal microscope (Zeiss). Pearson correlation coefficient was calculated using Zen Blue 2.3 (Zeiss).

### Quantitative PCR

Total RNA was extracted using the RNeasy Plus minikit (Qiagen) and reverse transcribed using Super RT reverse transcriptase (HT Biotechnology Ltd). PCR was performed on the ABI 7900HT Real-Time PCR system (Applied Biosystems; software: Quantstudio 1.3) using SYBR Green Master mix (Applied Biosystems). Reactions were performed with 125 ng of template cDNA. Transcript levels of genes were normalised to GAPDH.

### Measurements of 2-OG dependent dioxygenase activity

The in vitro HIF-1α prolyl hydroxylation activity of HeLa cell lysates against a His-tagged protein corresponding to residues 530–652 of human HIF-1α protein^4,15^ was performed by incubating 10 μM HIF-1α^530–652^ with 50 μl HeLa cell extracts in 20 mM HEPES (pH 7.5, 150 mM NaCl and 1 mM DTT) for 15 min at 37 °C. Reactions were terminated by the addition of SDS loading buffer, and proteins visualised by SDS-PAGE. Images were quantified using ImageJ, and are presented as the ratio of densitometry of hydroxylated to total HIF-1α^ODD^ peptide at the 15 min timepoint, normalised to PHD2.

For the 5hmC dot blot assay, genomic DNA was extracted from HeLa cells using a Gentra Puregene kit (Qiagen), and dot blotting for 5hmC levels performed by serial dilutions of denatured genomic DNA on Hybond NX membranes^4^. Following UV crosslinking the membranes were blocked with 1% bovine serum albumin and 5% milk powder prior to probing with a rabbit polyclonal antibody to 5hmC (Active Motif). Total DNA levels were evaluated by methylene blue staining, and relative densitometry measured using ImageJ.

For the KDM panel, cells were lysed in SDS lysis buffer and probed for selected H3 methylation marks.

### Mitochondrial protease protection assay

Mitochondria from 10^7^ HeLa cells were extracted using a Qproteome Mitochondria Isolation Kit (Qiagen). After the final wash with mitochondria storage buffer, mitochondria were divided into tubes, pelleted by centrifugation at 6000 × *g* and resuspended in either 10 mM Tris-HCl pH 8.0 with 250 mM sucrose (for whole mitochondria), or 10 mM Tris-HCl pH 8.0 (for mitoplasts), to a final protein concentration of 1 mg/ml. Proteinase K (Sigma P2308) was added to a final concentration of 12 or 24 µg/ml, based on methods previously described^53^. Following incubation at 37 °C for 30 min, the reaction was quenched with 1 mM PMSF. Mitochondria or mitoplasts were then pelleted again by centrifugation at 6000 × *g*, lysed in SDS buffer and analysed by immunoblot.

### Purification of ABHD11 from HEK293T cells

HEK293T cells were transfected with the pCEFL-ABHD11 -3XFLAG tag plasmid. In brief, cells were seeded in a 14 cm dish at 70% confluency and transfected using 270 μg polyethylenimine (Sigma) with 22.5 µg DNA in 6 ml Opti-MEM. Cells were harvested after 48 h, and lysed in TBS buffer (100 mM Tris-HCl pH 8.0, 140 mM NaCl) with 1% Triton X-100 and cOmplete Protease Inhibitor Cocktail (Roche). After centrifugation at 17,000 × *g*, the supernatant was pre-cleared using Sepharose CL-4B (GE Healthcare) and incubated overnight with FLAG M2 antibody conjugated beads (Sigma). Following five washes with TBS, ABHD11-FLAG was eluted using 100 mg/l 3xFLAG peptide (Sigma F4799), filtered using a 0.22 µm PVDF filter and separated using a Superdex 75 10/300 column on an Äkta-Pure liquid chromatography system (software: Unicorn 6.3). 500 µl fractions were collected and protein content visualised by SDS-PAGE and Coomassie staining. Protein identity was confirmed by LC-MS/MS.

### Liquid chromatography mass spectrometry

Samples were reduced, alkylated and digested in-gel using either trypsin, GluC or AspN. The resulting peptides were analysed by LC-MS/MS using an Orbitrap Fusion Lumos coupled to an Ultimate 3000 RSLC nano UHPLC equipped with a 100 µm ID × 2 cm Acclaim PepMap Precolumn (Thermo Fisher Scientific) and a 75 µm ID × 50 cm, 2 µm particle Acclaim PepMap RSLC analytical column. Loading solvent was 0.1% formic acid with analytical solvents A: 0.1% formic acid and B: 80% acetonitrile + 0.1% formic acid. Samples were loaded at 5 µl/min loading solvent for 5 min before beginning the analytical gradient. The analytical gradient was 3-40% B over 42 min rising to 95% B by 45 min followed by a 4 min wash at 95% B and equilibration at 3% solvent B for 10 min. Columns were held at 40 °C. Data were acquired in a DDA fashion with the following settings: MS1: 375-1500 Th, 120,000 resolution, 4 × 10^5^ AGC target, 50 ms maximum injection time. MS2: Quadrupole isolation at an isolation width of *m/z* 1.6, HCD fragmentation (NCE 30) with fragment ions scanning in the Orbitrap from *m/z* 110, 5 × 10^4^ AGC target, 100 ms maximum injection time. Dynamic exclusion was set to +/−10 ppm for 60 s. MS2 fragmentation was trigged on precursors 5 × 10^4^ counts and above.

Raw files were processed using PEAKS Studio (version 8.0, Bioinformatics Solutions Inc.). Searches were performed with either trypsin, GluC or AspN against a Homo sapiens database (UniProt reference proteome downloaded 26/01/18 containing 25,813 sequences) and an additional contaminant database (containing 246 common contaminants). Variable modifications at PEAKS DB stage included oxidation (M) and carbamidomethylatation with 479 built in modifications included at PEAKS PTM stage.

### p-Nitrophenyl ester hydrolysis assay

Hydrolase activity of ABHD11-FLAG (or a heat-inactivated control made by incubation at 90 °C for 5 min) was assayed by incubation in an assay buffer containing 50 mM Tris-HCl pH 7.4, 150 mM NaCl, 0.01% bovine serum albumin, 1.4% methanol, and 500 µM p-nitrophenyl acetate (Sigma N8130). 1.5 µg enzyme was added to 200 µl assay buffer, and the formation of p-nitrophenol assayed using a Clariostar plate reader (BMG Labtech), recording absorbance at 405 nm while incubating at 37 °C for 40 min. The rate of formation of p-nitrophenol was calculated from the slope of the absorbance curve, subtracting the slope of a blank containing only assay buffer and substrate, and calibrated against a standard curve of p-nitrophenol (software: Microsoft Excel for Mac 16).

### OGDHc activity assay

OGDHc activity was measured in whole cell lysates using a Biovision ketoglutarate dehydrogenase activity assay (Biovision K678), according to the manufacturer’s protocol. HeLa cells were lysed by three freeze-thaw cycles followed by passing 10 times through a 26-gauge needle. Activity was subtracted from a background control containing cell lysate but no substrate.

### Mitochondrial bioenergetics assay

Dynamic measurements of oxygen consumption rate and extracellular acidification were recorded using a Seahorse XFe24 (Agilent; software: Wave 2.3). HeLa cells were seeded 24 h beforehand at 1.5 × 10^4^ cells/well, and assayed using the manufacturer’s Mito Stress Test protocol.

### Stable isotope tracing by LC-MS

HeLa cells were seeded in five replicates in 6-well plates 27 h prior to metabolite extraction, with a sixth well per condition used for cell count. Twenty-four hours prior to extraction, media was changed to either DMEM without L-glutamine (Sigma D6546) supplemented with 10% FCS and 4 mM [U-^13^C_5_]L-glutamine (Cambridge Isotopes CLM-1822), or DMEM without glucose (Gibco 11966-025) supplemented with 10% FCS, 1 mM sodium pyruvate and 25 mM [U-^13^C_6_]D-glucose (Cambridge Isotopes CLM-1396). Two biological replicates were undertaken, with independent lentiviral transductions of the same cell line on different days. Metabolites were extracted on dry ice, after washing with ice cold PBS, with 1 ml per 10^6^ cells of extraction buffer, containing 50% methanol, 30% acetonitrile, 20% water and 100 ng/ml HEPES. To quantify the two enantiomers of hydroxyglutarate, a subset was derivatised using 50 mg/ml diacetyl-L-tartaric anhydride in 20% acetic acid/80% acetonitrile^4^.

### Immunoprecipitation and mass spectrometry detection of lipoylation

HeLa cells were lysed on ice for 30 min in a HEPES buffer (150 mM NaCl, 50 mM HEPES pH 7.0, 1% IGEPAL CA-630, 1 mM nicotinamide (Sigma), 5 mM tris(2-carboxyethyl)phosphine hydrochloride (Sigma), 1X cOmplete Protease Inhibitor Cocktail (Roche), and 1 mM PMSF). Lysates were centrifuged at 16,900 × *g* for 10 min. For LC-MS experiments, free thiols were blocked by addition of 10 mM N-ethyl maleimide (Sigma) and incubated on a rotator at 4 °C for 1 h.

Lysates were immunoprecipitated by incubation with protein G beads (GE 17-0168-01), firstly for 1 h to pre-clear, and then overnight with the DLST or DLAT antibodies (Supplementary Table 2). Resins were washed four times with Tris-buffered saline containing 0.1% IGEPAL CA-630, followed by three washes with Tris-buffered saline. Protein was eluted using 2% SDS, 50 mM Tris pH 7.4, 150 mM NaCl, 1 mM dithiothreitol and 10% glycerol, and incubated at 90 °C for 5 min. The proteins were then separated using SDS PAGE (Thermo NP0335), and visualised using SimplyBlue SafeStain (Invitrogen).

For mass spectrometry analysis, in-gel AspN digest and sample analysis were performed as previously described. To identify possible modifications of DLST K110, raw files were processed using PEAKS Studio (version 8.0, Bioinformatics Solutions Inc.) with the following parameters: AspN digestion; Human database (UniProt reference proteome downloaded 18 Dec 2018 containing 21,066 proteins) with additional contaminant database (containing 246 common contaminants); oxidation and carbamidomethylation as variable modifications at the PEAKS DB stage. The data were processed twice, with different variable modifications searched at the PEAKS PTM stage, either with 485 PEAKS built-in modifications listed in Supplementary Dataset 2 (sheet A) and 34 custom modifications listed in Supplementary Dataset 2 (sheet B).

XIC were obtained from Thermo Xcablibur Qual Browser (4.0.27.13). Mass ranges were limited to 564.79–564.80 *m/z*, 658.81–658.82 *m/z* and 784.87–784.88 *m/z* for the unmodified, lipoylated and 2x NEM lipoylated peptides respectively.

Label-free quantitation values were obtained by processing raw files with MaxQuant (version 1.6.6.0) with the following parameters: specific AspN digestion; Human database (UniProt reference proteome downloaded 18 Dec 2018 containing 21066 proteins); oxidation, lipoylation, 2x NEM lipoylation, N terminal acetylation as variable modifications; carbamidomethylation as a fixed modification; label-free quantification enabled. Label-free quantification values were normalised to the sum of DLST peptides label-free quantification values.

### ABHD11 structural prediction

A structural model of ABHD11 was obtained using the NCBI reference sequence for ABHD11 transcript variant 1 (NP_683710.1), modelled with Phyre2 against a template of murine epoxide hydrolase (PDB: 1cr6)^54^ and visualised using PyMOL 2.3 (Schrödinger, LLC). The mitochondrial targeting sequence was mapped with the MitoFates prediction tool^55^. Phylogenetic analysis and multiple sequence alignment of ABHD family members was performed with protein sequences obtained from Uniprot (canonical transcript variant) and aligned using Clustal Omega (EBI)^56^.

### Statistics and reproducibility

Statistical analysis of the screens was performed using MAGeCK version 0.5.5^51^, testing the sgRNA read counts obtained following the second sort against sgRNA read counts obtained from unsorted cells lysed at the same timepoint. Quantification and data analysis of other experiments are expressed as mean ± SEM and P values were calculated using two-tailed Student’s t-test for pairwise comparisons, unless otherwise stated, and were calculated using Graphpad Prism version 8. Metabolomic samples were blinded and randomised prior to their evaluation. Qualitative experiments were repeated independently to confirm accuracy. Specifically, Figs. 1e, f, g; 3e, h; 4f, i, j; 5d, f; Supplementary Figs. 1e, g, k, 5a, b; 9c were repeated twice with similar findings. Figs. 2l, m; 3d; 4d, e, h; 5e; Supplementary Figs. 1f; 4a, d; 7b and 9a were performed independently at least 3 times. Representative data are shown in the figures. Uncropped original scans of all immunoblots are displayed in Supplementary Fig. 11.

### Reporting summary

Further information on research design is available in the Nature Research Reporting Summary linked to this article.

## Supplementary information

Supplementary Information

Peer Review File

Supplementary Data 1

Supplementary Data 2

Reporting Summary

## Data Availability

SgRNA read count tables from CRISPR/Cas9 genetic screens are shown in Supplementary Dataset 1. The mass spectrometry proteomics data have been deposited to the ProteomeXchange Consortium via the PRIDE^57^ partner repository with the dataset identifier PXD020128 and the results and analysis are also available in Supplementary Dataset 2. LC-MS metabolic profiling has been deposited in MetaboLights^58^MTBLS1875. Source data are provided with this paper (Source Data File). Source data are provided with this paper.

## Source data

Source Data
