# Supplementary material for: ABHD11 maintains 2-oxoglutarate metabolism by preserving functional lipoylation of the 2-oxoglutarate dehydrogenase complex
Source: Nat Commun. 2020 Aug 13;11:4046. doi: 10.1038/s41467-020-17862-6 (PMC7426941; doi:10.1038/s41467-020-17862-6)
Supplement: Supplementary file 1 — Supplementary Information [file 41467_2020_17862_MOESM1_ESM.pdf]

Supplementary Information

ABHD11 maintains 2-oxoglutarate metabolism by preserving functional lipoylation of the 2-oxoglutarate dehydrogenase complex

Bailey et al.

Supplementary Figure 1. ABHD11 loss leads to HIF-1α accumulation and inhibition of 2-OG dependent dioxygenases in aerobic conditions.

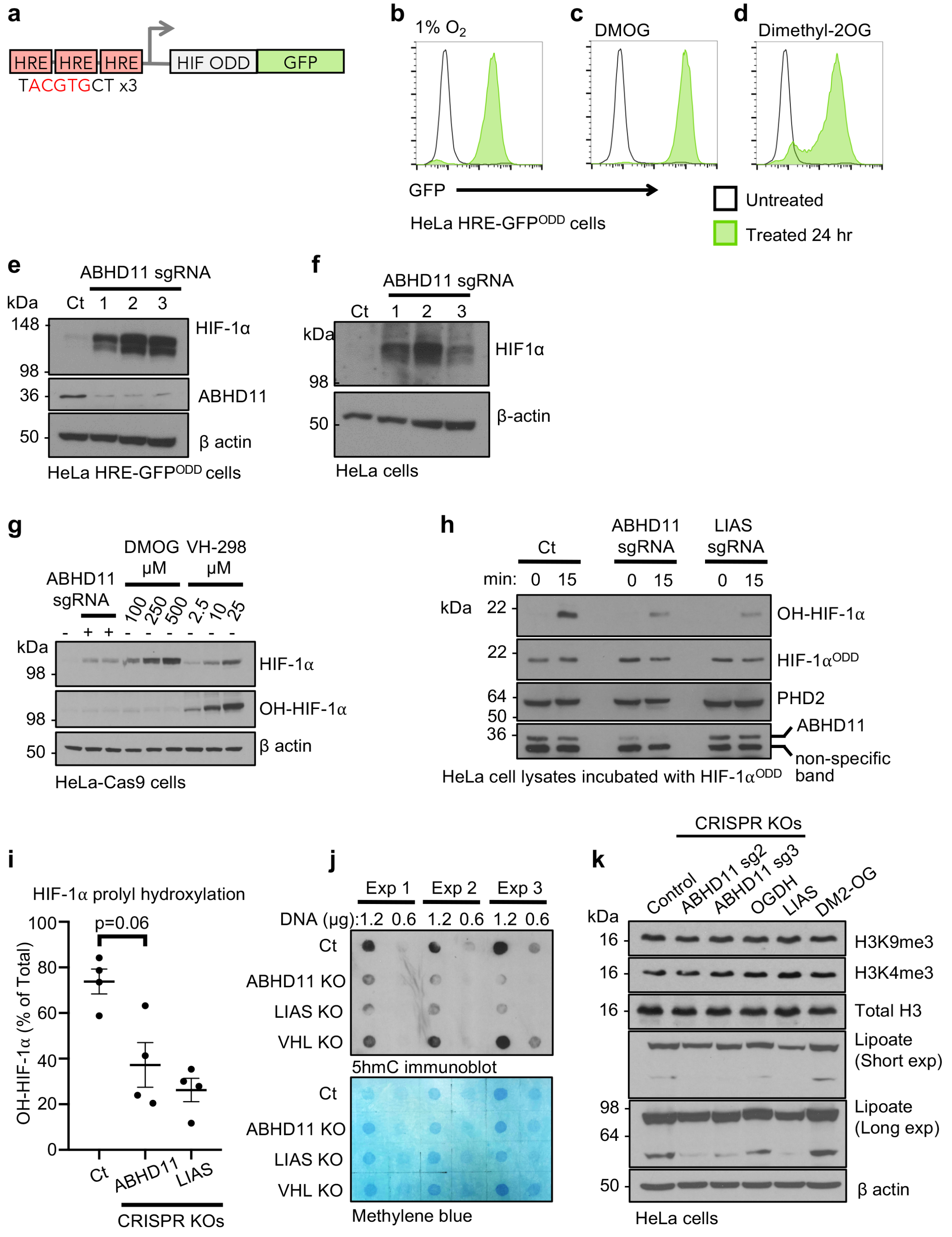

(**a**) Schematic of HRE-GFP^{ODD} reporter. (**b-d**) HeLa HRE-GFP^{ODD} cells respond to oxygen and metabolic inhibition. In 21% oxygen (O_2), GFP reporter levels are low (black line). Reporter

cells incubated for 24 hours in 1% O_2 (**b**) or following treatment with 1 mM DMOG (**c**), or 4 mM dimethyl 2-OG (DM-2OG) (**d**) accumulate GFP. n=10,000 cells per sample (**b**-**d**). (**e, f**) ABHD11 depletion leads to HIF-1α accumulation in cells incubated in 21% oxygen. HeLa HRE-GFP^{ODD} cells (**e**) or HeLa wildtype cells (**f**) expressing Cas9 were lentivirally transduced with up to 3 sgRNA targeting ABHD11 and HIF-1α levels measured by immunoblot. β-actin served as a loading control. (**g**) HIF-1α prolyl hydroxylation levels in HeLa cells following ABHD11 depletion, DMOG treatment or VHL inhibition. Control or ABHD11 deficient HeLa cells were generated as described. Hela cells were treated with indicated concentration of DMOG for 24 hr or the VHL inhibitor VH298 for 2 hours. Total HIF-1α and prolyl hydroxylated HIF-1α (OH-HIF-1α) were measured by immunoblot. (**h, i**) *In vitro* prolyl hydroxylation of HIF-1α. Recombinant HIF-1α, encoding the C-terminal ODD region (aa 530 – 652), was incubated with cell extracts from control, ABHD11 deficient or LIAS deficient cells for 15 min at 37°C. Total HIF-1α^{ODD} and prolyl hydroxylated HIF-1α^{ODD} (OH-HIF-1α) were measured by immunoblot (**h**) and quantified using ImageJ (**i**). *n=4 (representative image shown), mean ± SEM, two-tailled t test.* (**j**) 5hmC levels are reduced in ABHD11 depleted cells. Genomic DNA was extracted from Hela control or mixed KO populations of ABHD11, LIAS or VHL, and 5hmC levels measured by immunoblot, compared to total DNA content measured by methylene blue stain (**Figure 1j**). (**k**) Effect of ABHD11 loss on histone methylation marks. Control, ABHD11, OGDH or LIAS deficient HeLa cells were generated as previously described. Wildtype Hela cells were also treated with DM2-OG (6mM 24hr) as indicated. H3K4me3, H3K9me3 and total H3 levels were measured by immunoblot. Short and long lipoate immunoblot exposures are shown.

Supplementary Figure 2. ABHD11 is a member of the $\alpha\beta$ hydrolase domain containing family

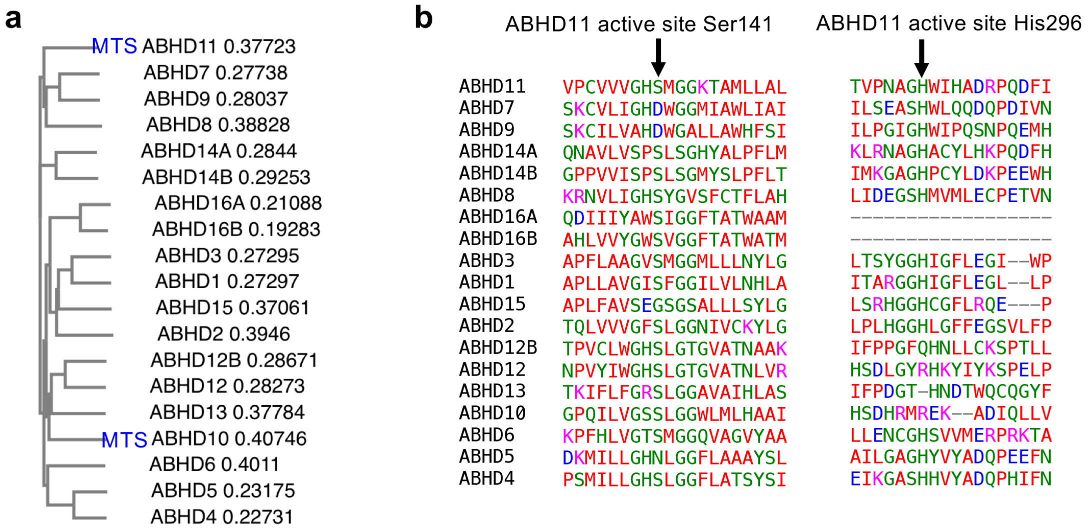

(**a**) Hierarchical clustering of canonical protein sequences of ABHD family members using Clustal Omega. ABHD11 and ABHD10 contain mitochondrial targeting sequences (MTS). (**b**) Multiple sequence alignment of the canonical transcript variant of ABHD family members using Cluistal Omega, showing the regions aligning to ABHD11 active site residues serine 141 and histidine 296. Residues are coloured by amino acid properties (red: small/hydrophobic; blue: acidic; magenta: basic; green: hydroxyl/sulfhydryl/amine, or glycine).

Supplementary Figure 3. ABHD11 is a mitochondrial matrix hydrolase.

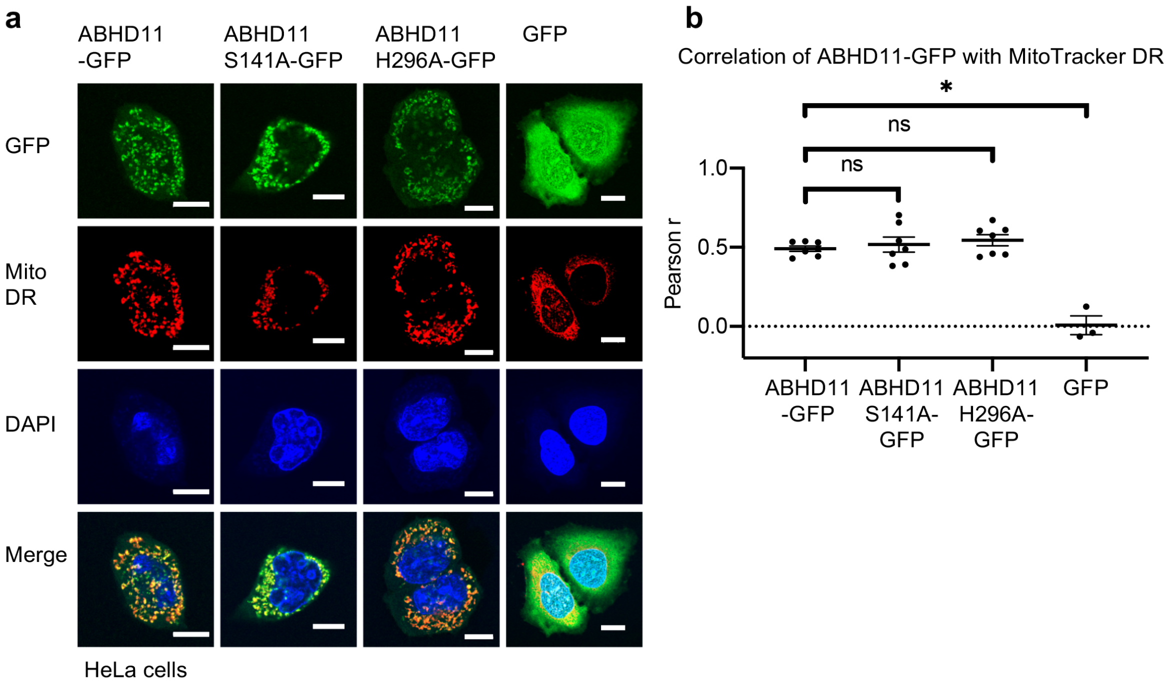

(**a, b**) Confocal immunofluorescence microscopy of Hela cells stably expressing ABHD11-GFP, ABHD11 S141-GFP or ABHD11 H296A-GFP (**a**). MitoTracker Deep Red was used to visualise mitochondria. Pearson correlation coefficient was used to quantify ABHD11 colocalisation with mitochondria (**b**). *Scale=10μm*. *p=0.017, two-tailed Mann Whitney U test. *ns=not significant (S141A vs wt: p=0.90; H296A vs wt: p=0.32).*

Supplementary Figure 4. Affinity purification of ABHD11-FLAG.

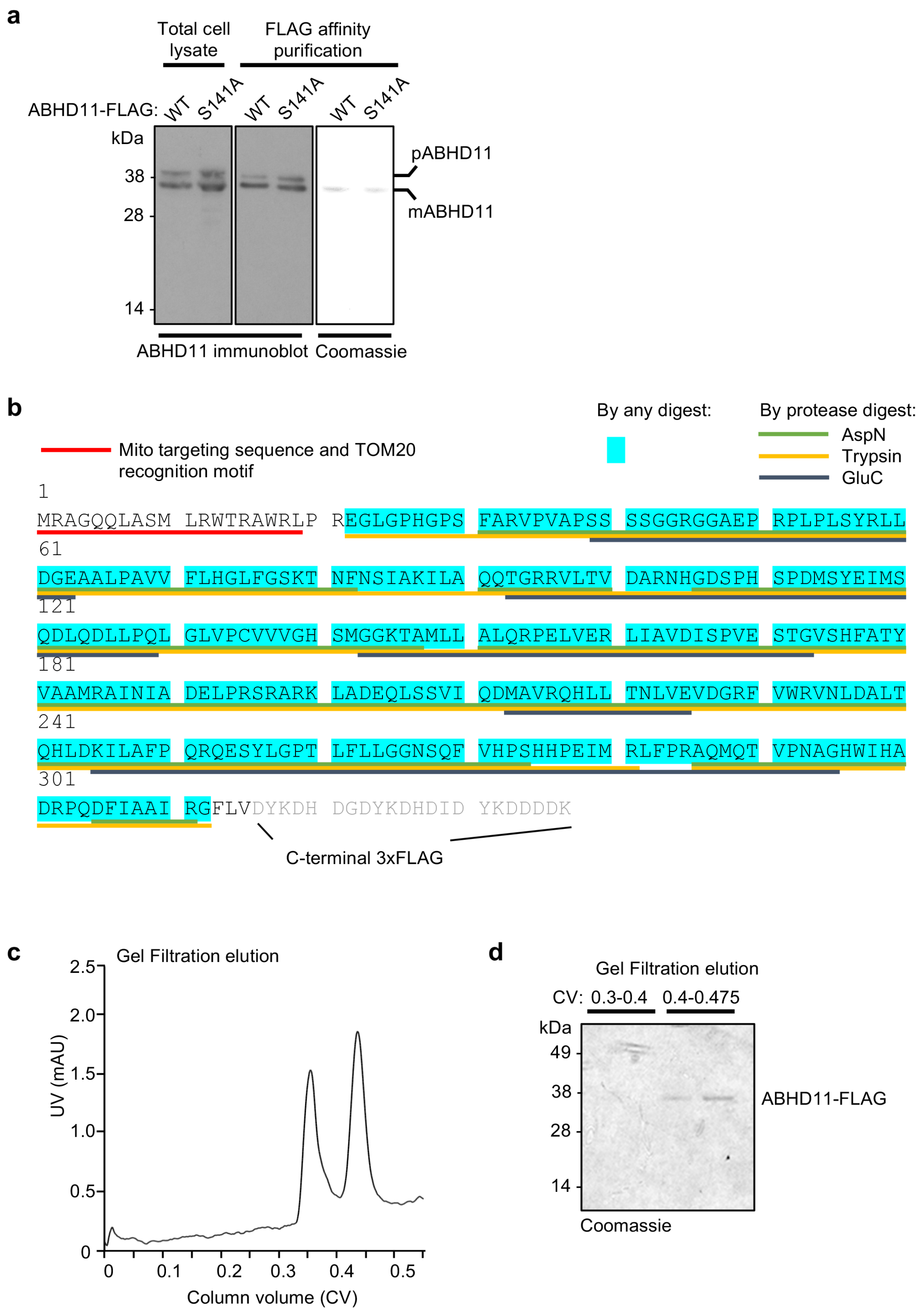

(**a**) Purification of wildtype or S141A mutant ABHD11 from human cells. HEK293T cells were transfected with wildtype of S141A ABHD11-FLAG. Cells were lysed after 48 hr (left panel)

and ABHD11 affinity purified using anti-FLAG affinity beads and FLAG peptide elution (middle (immunoblot) and right panel (Coomassie staining)). The pre-cleaved (pABHD11) and mitochondrial cleaved (mABHD11) forms are indicated. (**b**) Mass spectrometry (MS) sequence analysis of affinity purified ABHD11-FLAG expressing HEK293T cells. Affinity purified ABHD11 was subjected to SDS-PAGE and analysed by MS using three different peptide digests (Trypsin, AspN or GluC). Complete peptide coverage was observed aside from the first 21 residues encoding the mitochondrial targeting sequence and TOM20 recognition motif. (**c, d**) Size exclusion chromatography of ABHD11-FLAG. Affinity purified ABHD11-FLAG was subjected to gel filtration using a Superdex 75 10/300 GL column. Two peaks were visualised, potentially corresponding to monomeric and dimeric forms. ABHD11-FLAG was only visualised in the second peak elution (**d**).

Supplementary Figure 5. ABHD11 loss does not alter PDHc activity.

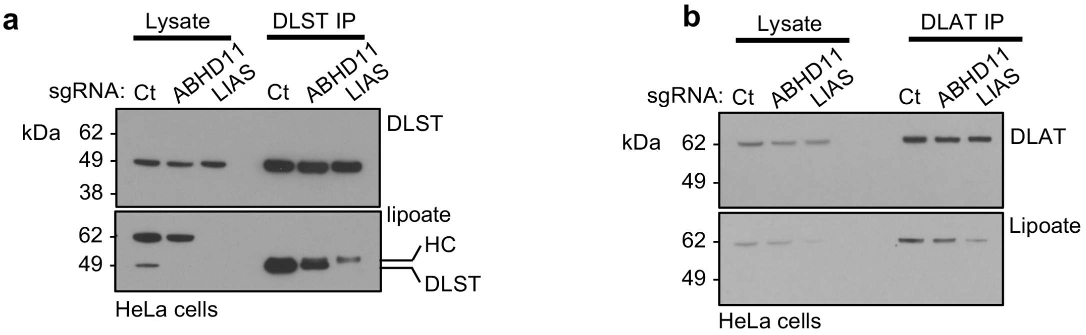

(**a**) Lipoylation of immunoprecipitated DLST. Control, ABHD11 mixed KO or LIAS mixed KO cells were lysed in 1% IGEPAL CA-630 and DLST immunoprecipitated. DLST and lipoylated DLST levels are shown. *HC = heavy chain.* (**b**) Lipoylation of immunoprecipitated DLAT. Control, ABHD11 mixed KO or LIAS mixed KO cells were lysed in 1% IGEPAL CA-630 and DLAT immunoprecipitated. DLAT and lipoylated DLAT levels are shown.

Supplementary Figure 6. ABHD11 loss does not alter PDHc activity.

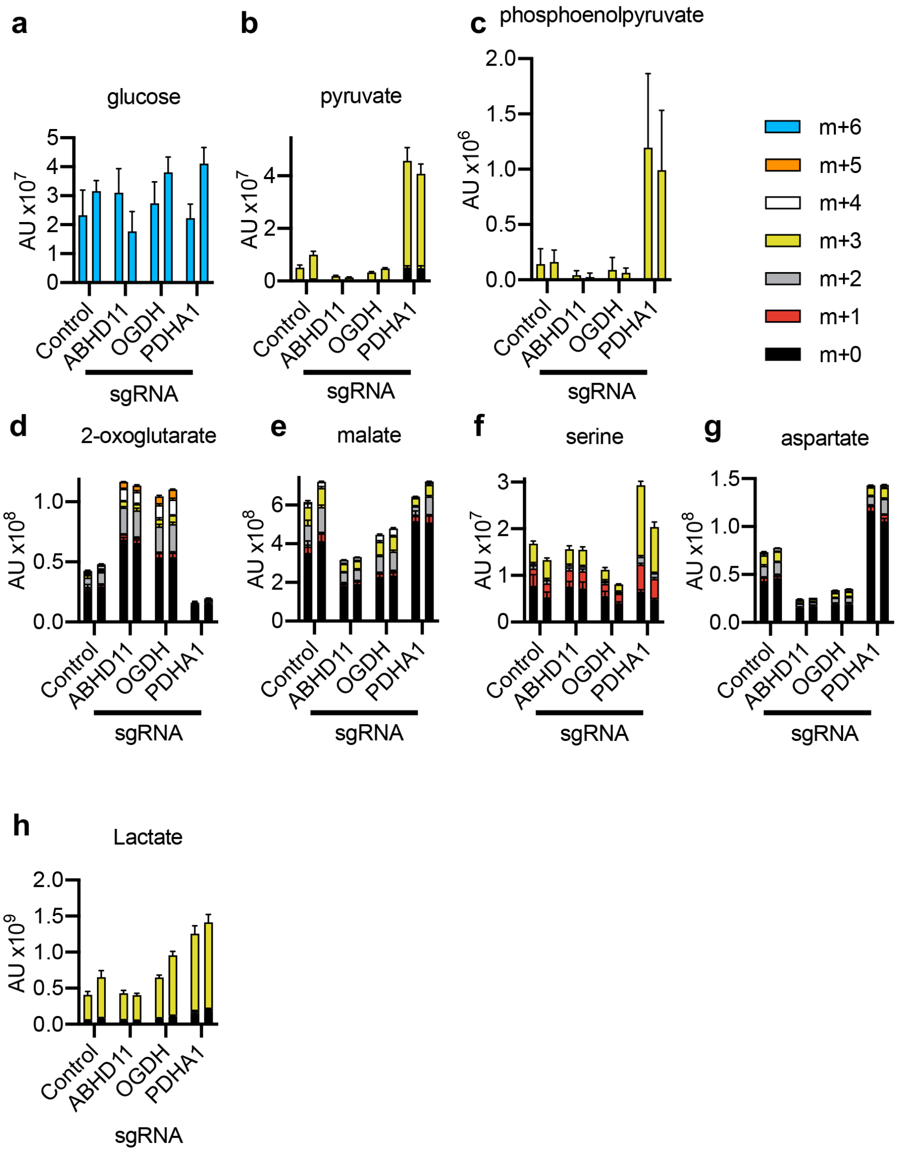

(**a-h**) Stable isotope tracing in HeLa cells compared to mixed CRISPR KO populations (sgRNA) of ABHD11, OGDH or pyruvate dehydrogenase E1 alpha subunit (PDHA1) incubated with [U-^{13}C6]-glucose. Isotopologues of pyruvate (**b**), and phosphoenolpyruvate (PEP) (**c**) confirm that PDHA1 loss impaired PDHc function, resulting in pyruvate and PEP accumulation. Isotopologues of 2-OG (**d**) and malate (**e**) confirm that ABHD11 and OGDH loss impairs the TCA cycle by decreased OGDHc activity, within increased 2-OG and decreased malate. Serine (**f**) and aspartate (**g**) increases following PDHA1 loss are consistent with decreased PDHc

function. ABHD11 and OGDH loss do not increase serine or aspartate pools (**f, g**). (**h**) Lactate levels are increased with PDHA1 loss but not with ABHD11 or OGDH depletion. Two biologically independent samples are shown; n=5 technical replicates per sample, mean ± SD. The m+0 to m+5 isotopologues are indicated.

Supplementary Figure 7. ABHD11 prevents the formation of lipoyl adducts on the OGDHc

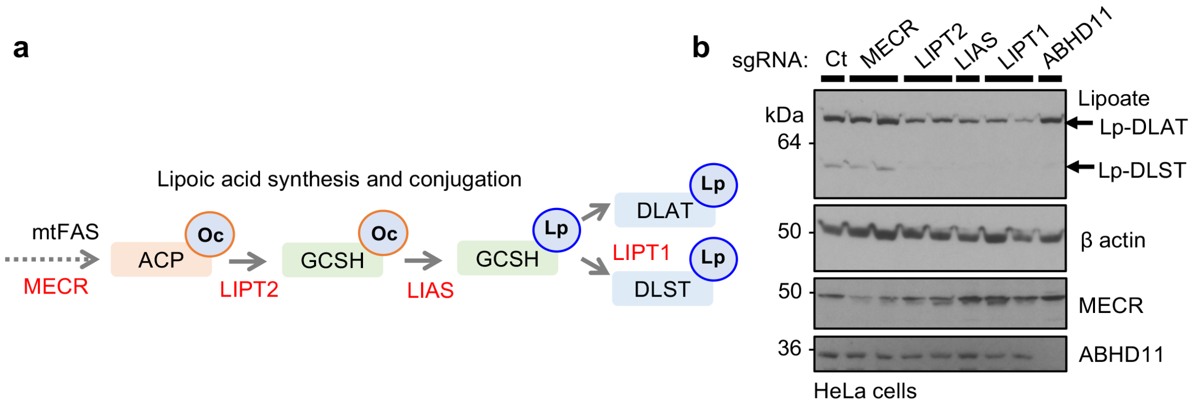

(**a**) Schematic of lipoic acid synthesis and conjugation pathway. Mitochondrial fatty acid synthesis provides the octanoylated (Oc) precursor for lipoic acid synthesis. MECR is upstream of octanoylated ACP, converting Trans-2-enoyl-ACP to acyl-ACP. LIPT2 catalyses the transfer of octanoate to GCSH. LIAS converts octanoate to lipoate. LIPT1 is thought to be the major lipoyl transferase allowing conjugation to conserved lysine residues in 2-oxoacid dehydrogenase subunits such as DLAT and DLST. (**b**) Depletion of lipoic acid synthesis and conjugation components in HeLa cells. Mixed HeLa KO populations of MECR, LIPT2, LIPT1, LIAS and ABHD11 were generated and immunoblotted for lipoylated proteins. β-actin served as a loading control.

Supplementary Figure 8. The effect of ABHD11 depletion or inhibition on cell growth and mitochondrial ROS.

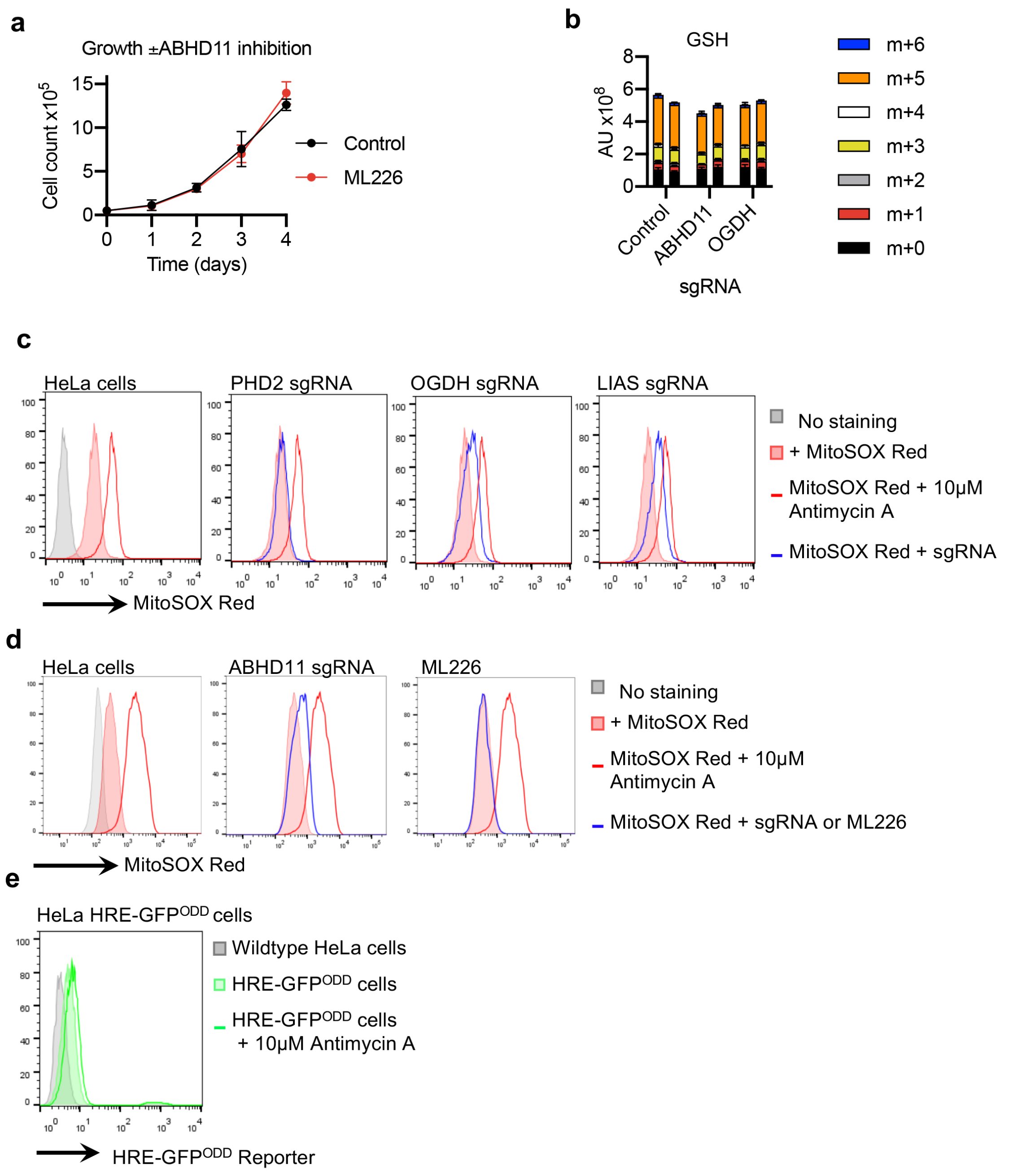

(**a**) ML226 treatment does not alter cell growth. HeLa cells were treated with 1μM ML226 or a DMSO control, and cell growth monitored over 4 days (n=3 biologically independent samples, mean ± SD). (**b**) LC-MS quantification of glutathione (GSH) isotopologues from [U-$^{13}C_5$] glutamine treated control HeLa cells compared to mixed CRISPR KO populations

(sgRNA) of ABHD11 or OGDH (two biologically independent samples; n=5 technical replicates per sample, mean ± SD). (**c, d**) Mitochondrial ROS levels following OGDH, LIAS, PHD2 or ABHD11 depletion. HeLa Cas9 cells were transduced with sgRNA targeting OGDH, LIAS, PHD2 (**c**) or ABHD11 (**d**). After 10-13 days cells were stained with MitoSOX Red, and fluorescence measured by flow cytometry. Treatment with Antimycin A (10 μM, 30 min 37 °C) was used as a positive control for mitochondrial ROS. Example of gating strategy is shown in **Supplementary Figure 10b**. (**e**) HRE-GFP^{ODD} reporter levels following treatment with Antimycin A (10μM) for 30 min at 37° C showed no change in GFP fluorescence compared to control cells treated with DMSO.

Supplementary Figure 9. ABHD11 prevents the formation of lipoyl adducts on the OGDHc

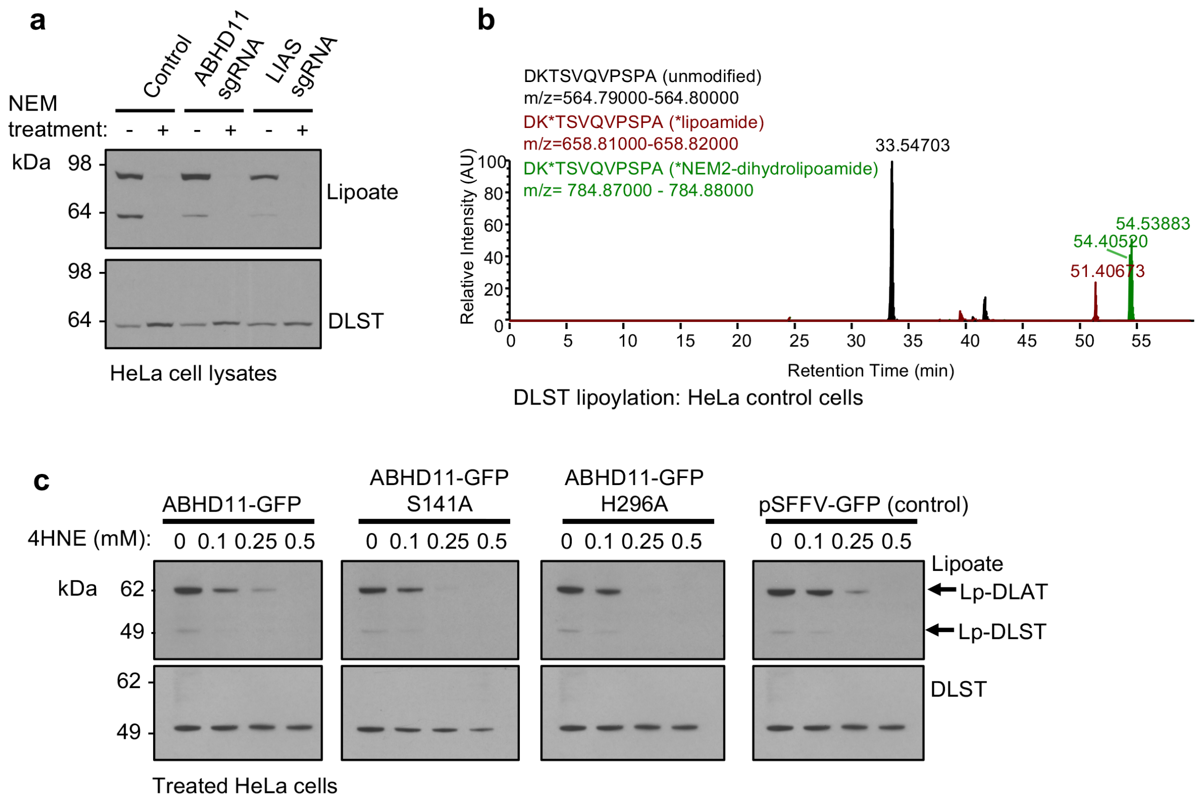

(**a**) Effect of N-ethyl maleimide (NEM) treatment on protein lipoylation detected by immunoblot. Control or ABHD11 deficient HeLa cells were lysed in a HEPES buffer, and free thiols in HeLa cells were blocked with the addition of 10mM NEM (4°C, 1 hour). Immunoblot of lipoylated proteins confirmed efficient blocking of free thiols. (**b**) Representative chromatograms of DLST peptide encoding the lipoylated region from HeLa cells, demonstrating that the m/z separation of the differently modified DLST peptide (DK*TSVQVPSPA). (**c**) Overexpression of ABHD11 catalytic inactive mutants renders cells more susceptible to lipoyl adduct formation. HeLa cells expressing ABHD11-GFP, ABHD11 S141A-GFP, ABHD11 H296A-GFP or a GFP only lentivirus (pSFFV-GFP) were treated with 4-HNE at the concentrations indicated, and lipoylation levels measured using the anti-lipoate antibody. Loss of lipoylation represents lipoyl adduct formation.

Supplementary Figure 10. Flow cytometry gating

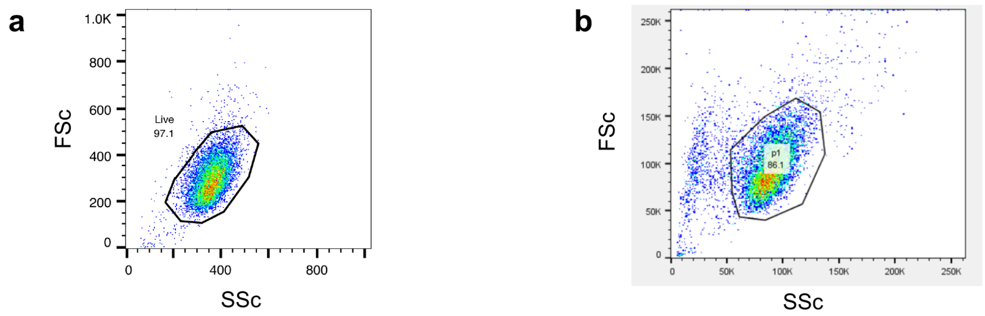

(**a**, **b**) Example of gating strategy from control samples of **Figure 1d** (**a**) and **Supplementary Figure 8d** (**b**). Cells are gated on forward scatter (FSc) and side scatter (SSc) to exclude cell fragments; no other gating or exclusion of data was performed. The percentage of live cells included in the subsequent analysis is displayed.

Supplementary Figure 11. Uncropped original scans

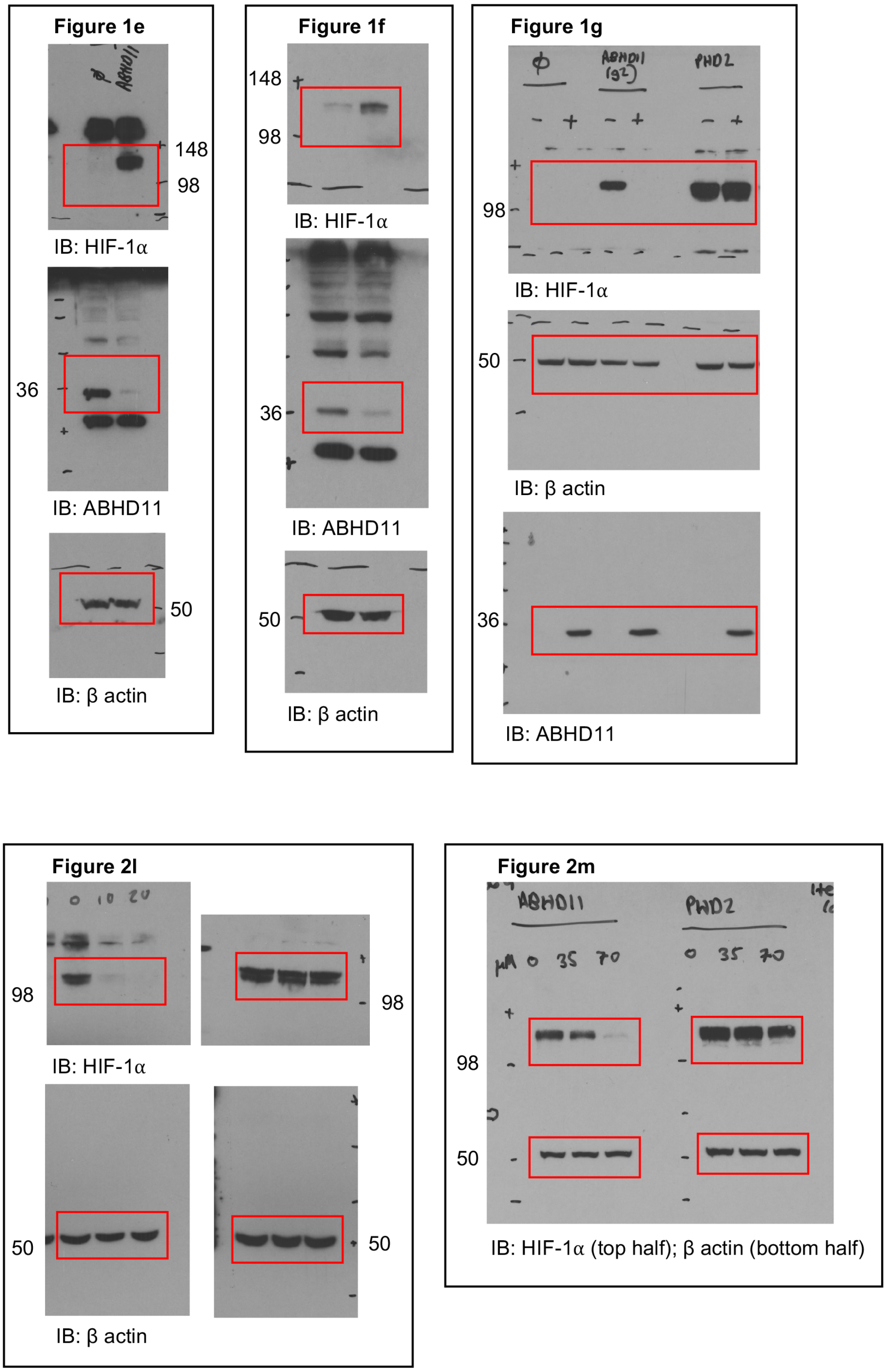

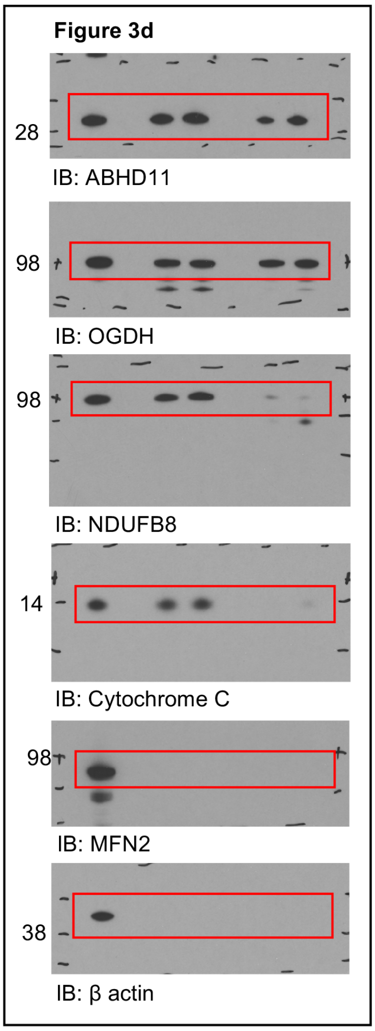
Figure 3d
28
IB: ABHD11
98
IB: OGDH
98
IB: NDUFB8
14
IB: Cytochrome C
98
IB: MFN2
38
IB: β actin

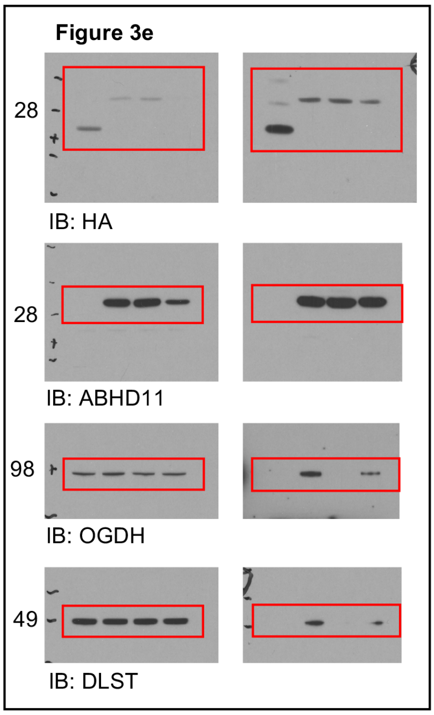
Figure 3e
28
IB: HA
28
IB: ABHD11
98
IB: OGDH
49
IB: DLST

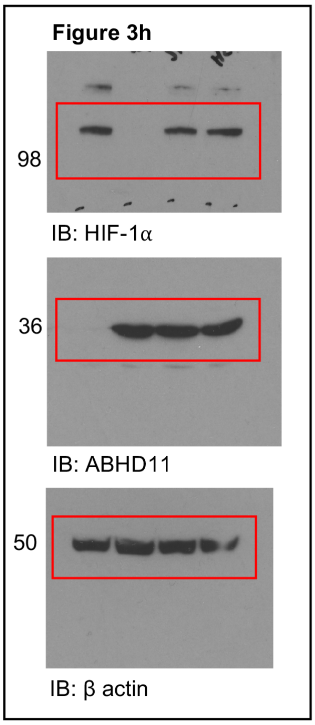
Figure 3h
98
IB: HIF-1α
36
IB: ABHD11
50
IB: β actin

Figure 4b

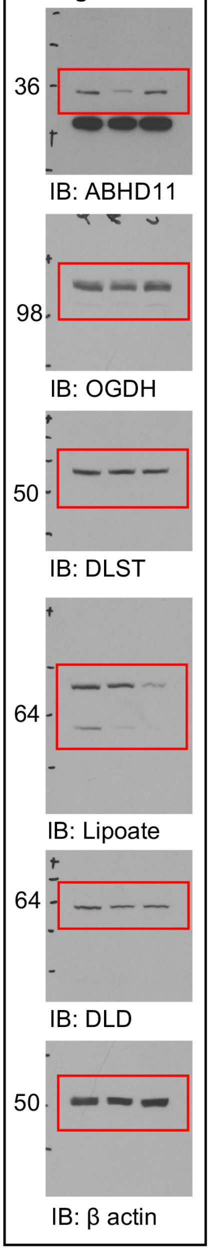

Figure 4d

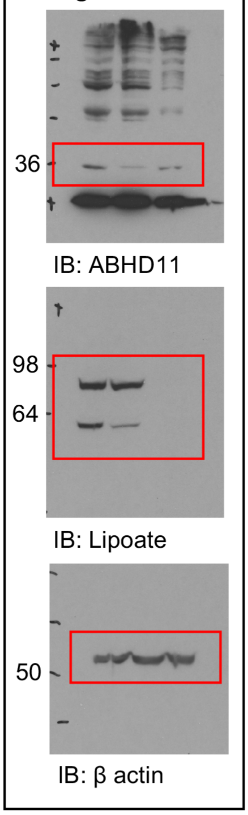

Figure 4e

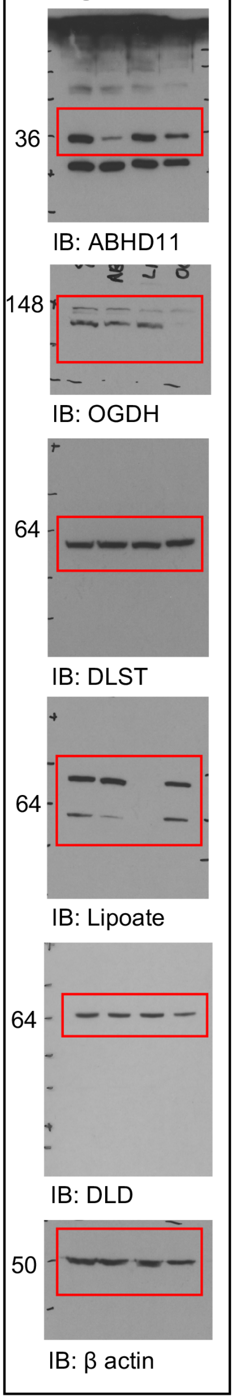

Figure 4f

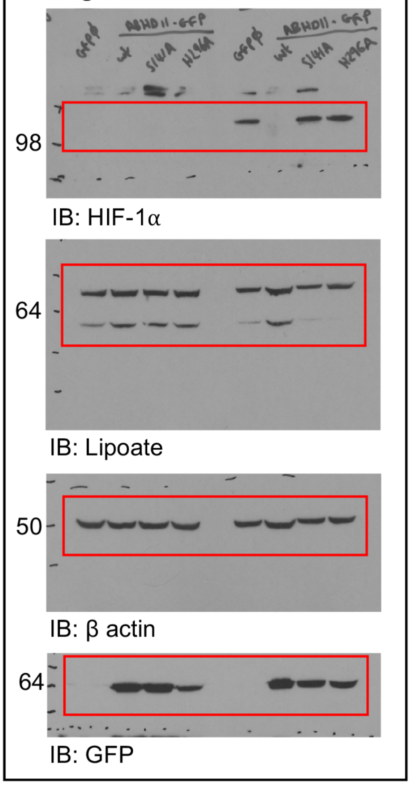

Figure 4h

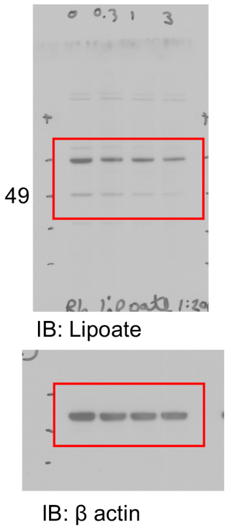

Figure 4i

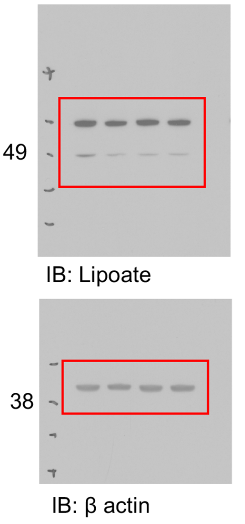

Figure 4j

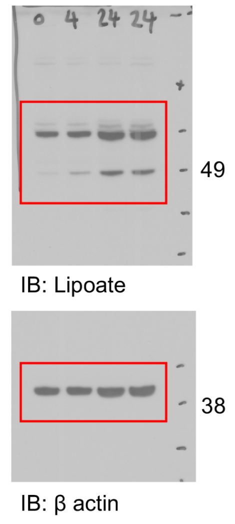

Figure 5d

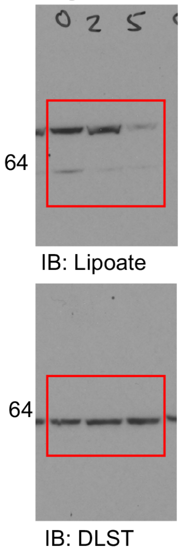

Figure 5e

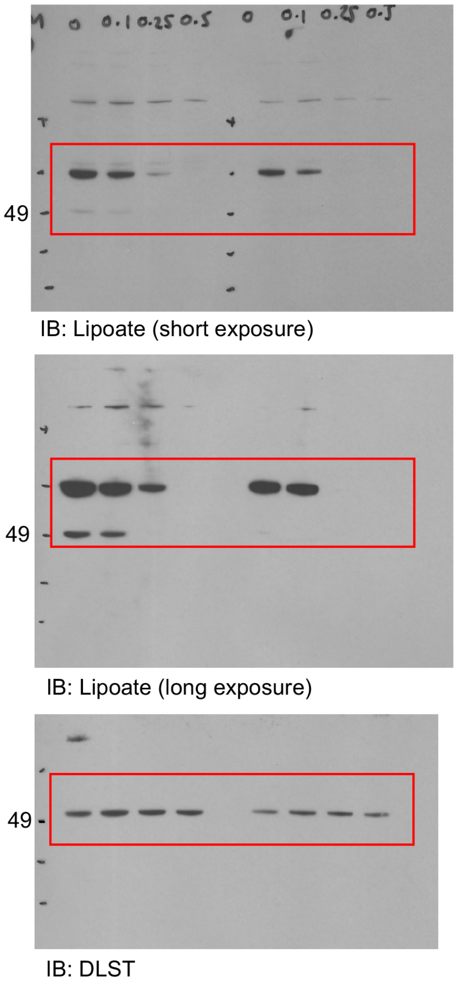

Figure 5f

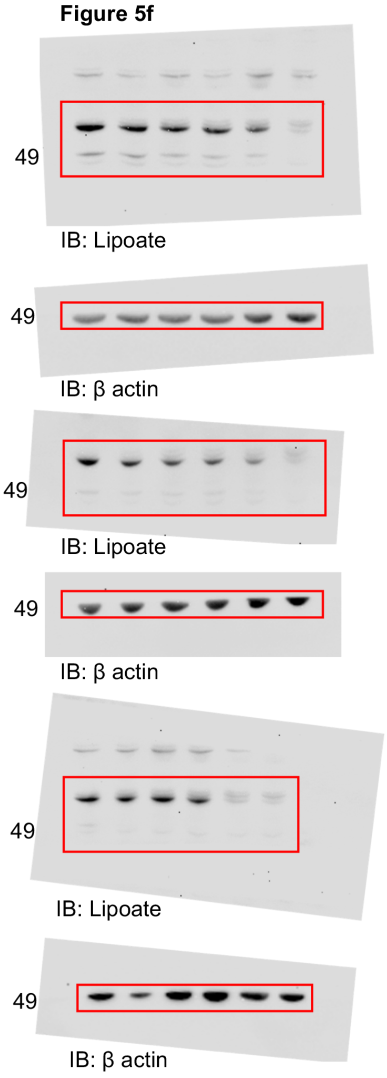

Supplementary Figure 1e

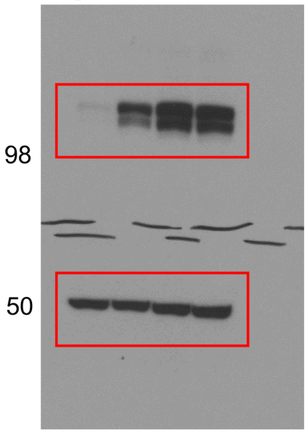

IB: HIF-1α (top)
β actin (bottom)

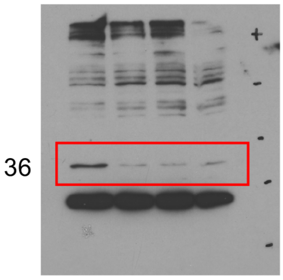

IB: ABHD11

Supplementary Figure 1f

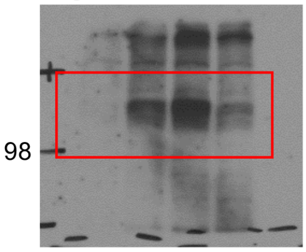

IB: HIF-1α

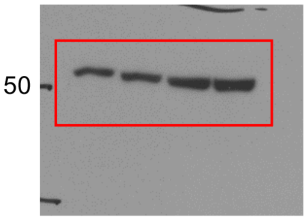

IB: β actin

Supplementary Figure 1g

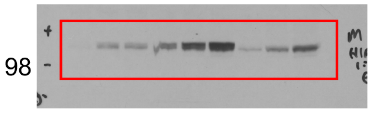

IB: HIF-1α

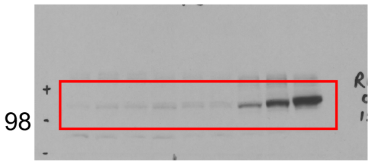

IB: OH-HIF-1α

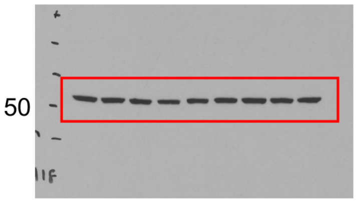

IB: β actin

Supplementary Figure 1h

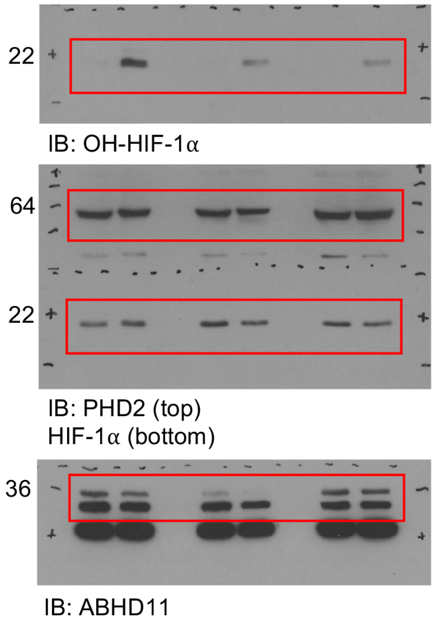

Supplementary Figure 1k

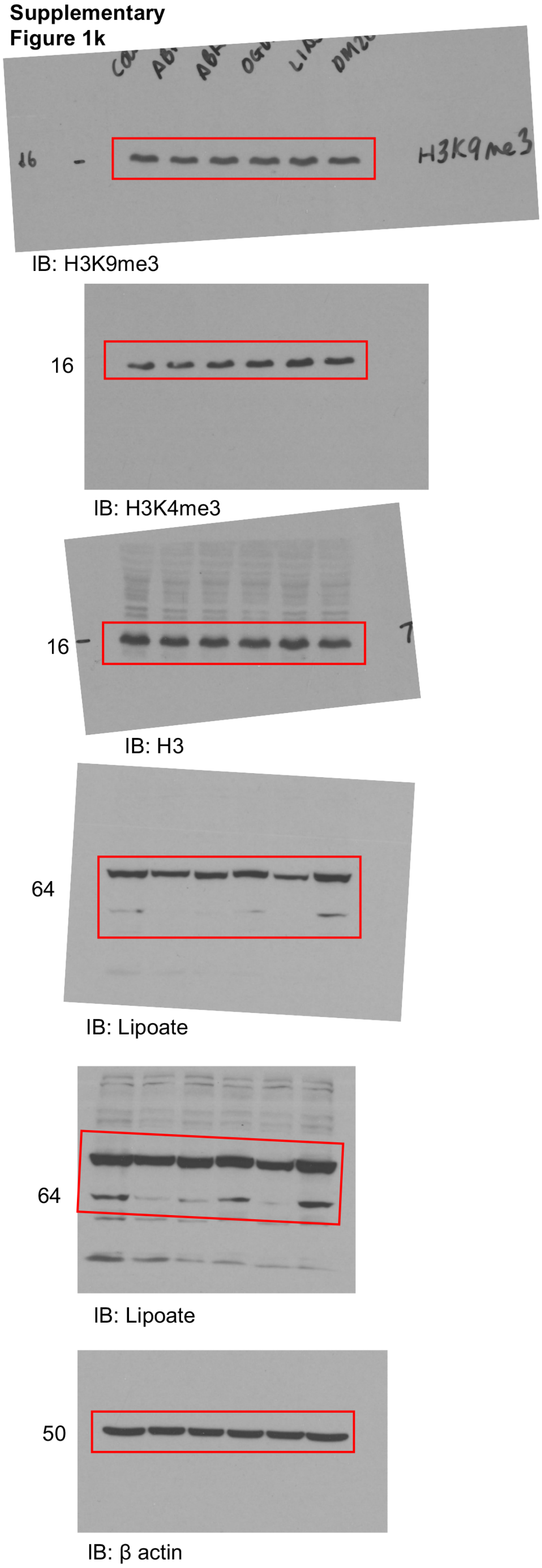

Supplementary Figure 5a

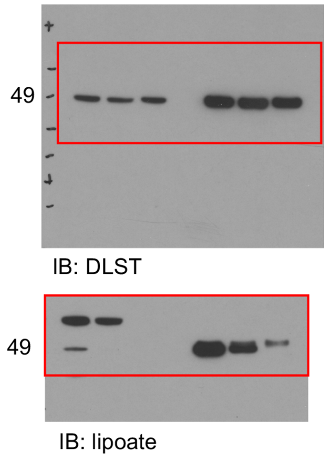

Supplementary Figure 5b

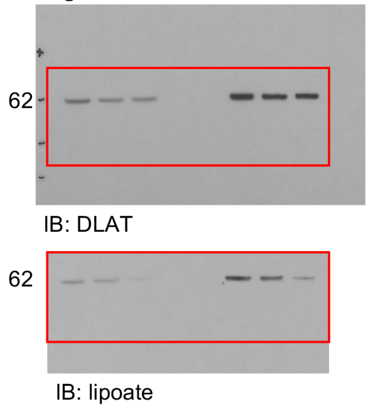

Supplementary Figure 7b

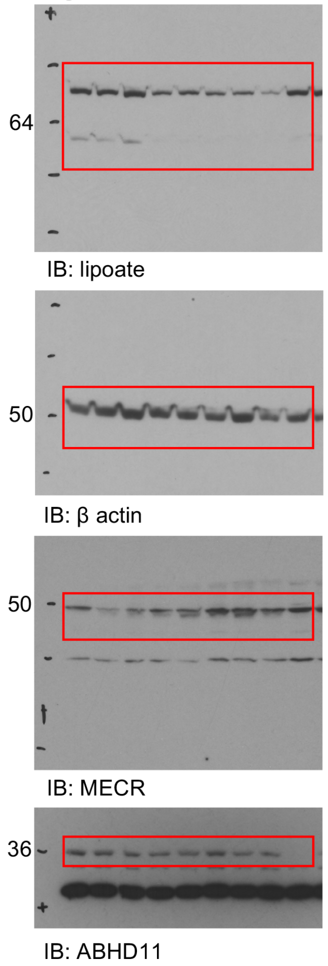

Supplementary Figure 9a

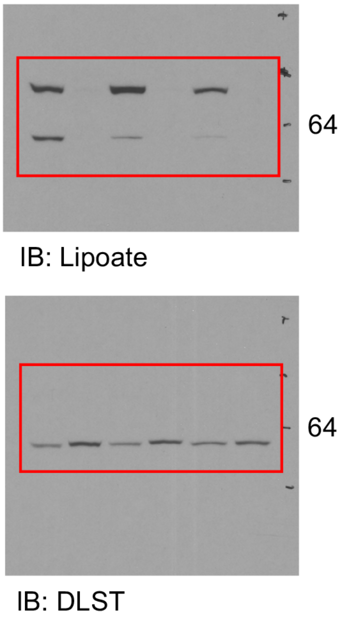

Supplementary Figure 9c

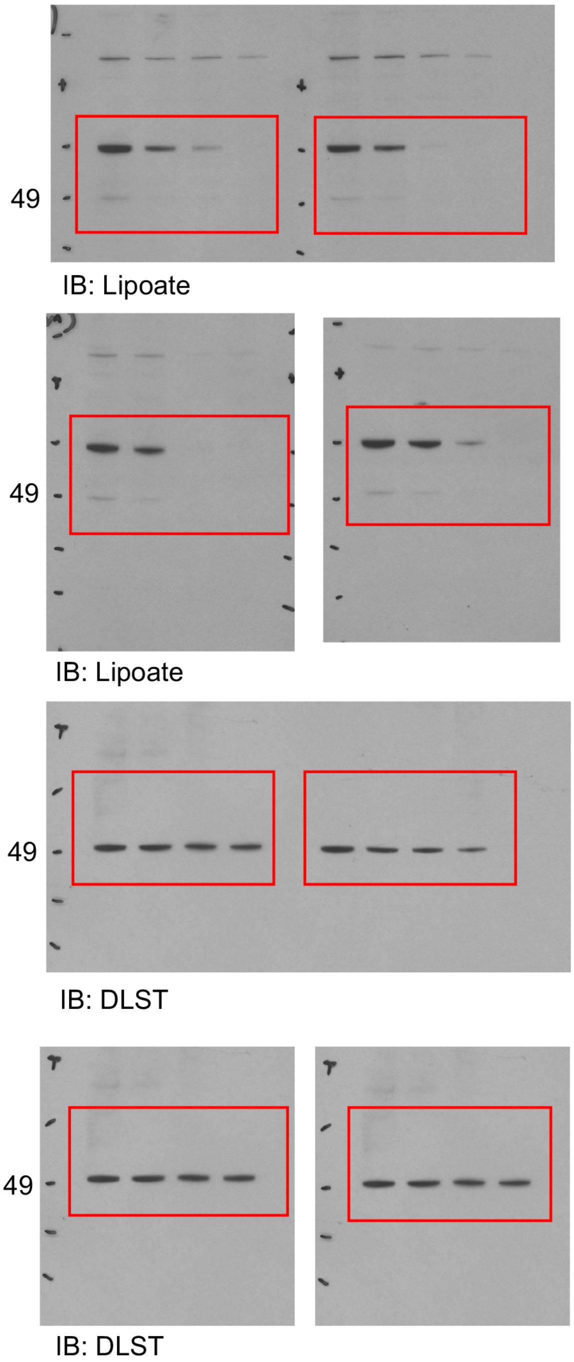

Supplementary Table 1

Gene symbol	Protein name	Peptide count			
		Control	ABHD11-HA		
			WT	S141A	H296A
ABHD11	ABHD11	1	666	500	308
HSPA9	Stress-70 protein	16	34	32	41
HSPD1	60 kDa heat shock protein	12	33	18	37
LRPPRC	Leucine-rich PPR motif-containing protein	24	17	12	8
NME4	Nucleoside diphosphate kinase	18	19	13	13
ATP5A1	ATP synthase subunit alpha	8	12	7	5
ATP5B	ATP synthase subunit beta	9	13	7	5
SSBP1	Single-stranded DNA-binding protein	7	14	6	3
MRPS27	28S ribosomal protein S27	13	6	5	4
TFAM	Transcription factor A	8	4	4	5
GCDH	Glutaryl-CoA dehydrogenase	11	6	4	6
MRPS34	28S ribosomal protein S34	7	5	8	2
CPS1	Carbamoyl-phosphate synthase	1	8	2	10
ACAD9	Acyl-CoA dehydrogenase family member 9	7	5	4	6
SLC25A3	Phosphate carrier protein	5	5	2	5
TUFM	Elongation factor Tu	3	8	3	2
PGAM5	Serine/threonine-protein phosphatase PGAM5, mitochondrial	3	4	5	5
SHMT2	Serine hydroxymethyltransferase	1	7	1	3
SLC25A1	Tricarboxylate transport protein	5	2	2	4
DLST	Dihydrolipoyllysine-residue succinyltransferase	1	4	2	2
DLAT	Dihydrolipoyllysine-residue acetyltransferase	1	3	3	1
OGDH	2-oxoglutarate dehydrogenase	0	3	2	4

Top mitochondrial proteins identified with immunoprecipitation-LC-MS using ABHD11-HA overexpressed in HeLa cells. The number of confidently (>90%) assigned unique peptides identified in each sample by Scaffold (Proteome Software, Inc) is displayed on the right, for each protein in each sample. The gene symbol and name of the top 23 proteins (out of 53), ordered by total peptide count, is displayed.

Supplementary Table 2

**Reagents and Antibodies**	Source	Identifier
**Antibodies**		
Rabbit polyclonal anti-5hmC<br>Dot blot: 1:10,000	Active Motif	Cat#39770; RRID: AB_10013602<br>Lot 2158003
Rabbit polyclonal anti-ABHD11<br>Immunoblot: 1:2000	Signalway	Cat#34366<br>Lot 4813
Mouse monoclonal anti-β actin<br>Immunoblot: 1:20,000	Sigma	Cat#A2228; RRID: AB_476697<br>Lot 112M4762V
Mouse monoclonal anti-Cytochrome C<br>Immunoblot: 1:5000	Abcam	Cat#ab110325; RRID: AB_10864775<br>Clone 37BA11
Rabbit polyclonal anti-DLD<br>Immunoblot: 1:2000	GeneTex	Cat#GTX101245; RRID: AB_1240715<br>Lot 39506
Mouse monoclonal anti-DLAT<br>Immunoblot: 1:2000	Cell Signalling	Cat#12362S; RRID: AB_2797893<br>Clone 4A4-B6-C10<br>Lot 1
Mouse monoclonal anti-DLST (used for IP)	Abcam	Cat#ab110306; RRID: AB_10862702<br>Clone 9F4BD5<br>Lot 3278422-1
Rabbit monoclonal anti-DLST (used for immunoblot)<br>Immunoblot: 1:2000	Cell Signalling	Cat#11954; RRID: AB_2732907<br>Clone D22B1
Mouse monoclonal anti-GFP<br>Immunoblot: 1:2000	Roche	Cat#11814460001; RRID: AB_390913<br>Clones 7.1 & 13.1
Rabbit monoclonal anti-H3<br>Immunoblot: 1:2000	Cell Signalling	Cat#4499; RRID: AB_10544537<br>Clone D1H2
Rabbit monoclonal anti-H3K4me3<br>Immunoblot: 1:1000	Cell Signalling	Cat#9751; RRID: AB_2616028<br>Clone C42D8
Rabbit monoclonal anti-H3K9me3<br>Immunoblot: 1:1000	Cell Signalling	Cat# 13969; RRID: AB_2798355<br>Clone D4W1U
Rat monoclonal anti-HA<br>Immunoblot: 1:2000	Roche	Cat#11867423001; RRID: AB_390918<br>Clone 3D10
Mouse monoclonal anti-HIF-1α<br>Immunoblot: 1:1000	BD Biosciences	Cat#610959; RRID: AB_398272<br>Lot 9049717
Rabbit monoclonal anti-Hydroxy-HIF-1α<br>Immunoblot: 1:1000	Cell Signalling	Cat#3434; RRID: RRID:AB_2116958<br>Clone D43B5<br>Lot 6

Rabbit polyclonal anti-Lipoic acid<br>Immunoblot: 1:2000	Calbiochem	Cat#437695; RRID: AB_212120<br>Lot 3308897
Rabbit polyclonal anti-MECR<br>Immunoblot: 1:1000	Proteintech	Cat#51027-2-AP; RRID: AB_615013<br>Lot 1537
Mouse monoclonal anti-MFN2<br>Immunoblot: 1:1000	Abcam	Cat#ab56889; RRID: AB_2142629<br>Clone 6A8
Mouse monoclonal anti-NDUFB8<br>Immunoblot: 1:2500	Abcam	Cat#ab110242; RRID: AB_10859122<br>Clone 20E9DH10C12
Rabbit polyclonal anti-OGDH<br>Immunoblot: 1:1000<br>Immunofluorescence microscopy: 1:100	Atlas Antibodies	Cat#HPA020347; RRID: AB_1854773<br>Lot A74004
Rabbit polyclonal anti-PHD2 (EGLN1)<br>Immunoblot: 1:2000	Novus	Cat#NB100-137; RRID: AB_10003054<br>Lot A3
Alexa-Fluor 647 Goat Anti-Rabbit IgG<br>Microscopy secondary: 1:1000	Thermo	Cat#A21245; RRID:AB_2535813<br>Lot 1558736
Peroxidase-AffiniPure Goat Anti-Mouse IgG<br>Immunoblot secondary: 1:20,000	Jackson	Cat#115-035-146; RRID:AB_2307392
Peroxidase-AffiniPure Goat Anti-Rabbit IgG<br>Immunoblot secondary: 1:20,000	Jackson	Cat#111-035-045; RRID: AB_2337938
Peroxidase-AffiniPure Goat Anti-Rat IgG<br>Immunoblot secondary: 1:20,000	Jackson	Cat#112-035-167; RRID: AB_2338139
**Reagents**		
Antimycin A	Alfa Aesar	Cat#J63522; CAS: 1397-94-0s
Dimethyloxalylglycine	Cayman Chemical	Cat#71210; CAS: 89464-63-1
FCCP	Cayman Chemical	Cat#15218; CAS: 370-86-5
Sodium oxamate	Sigma	Cat# O2751; CAS: 565-73-1
GSK-2837808A	Tocris	CAS: 1445879-21-9
ML226	Cayman Chemical	Cat#25681; CAS: 2055172-43-3
Oligomycin A	Cayman Chemical	Cat#11342; CAS: 579-13-5
Rotenone	Sigma	Cat#R8875; CAS: 83-79-4
VH298	A gift from Alessio Ciulli	
MitoSOX Red	ThermoFisher Scientific	Cat#M36008
3xFLAG Peptide	Sigma	Cat#F4799
His-HIF-1α^{ODD} (aa530-652)	Burr et al, 2016	

Supplementary Table 3

**CRISPR sgRNA oligonucleotide sequences**	
ABHD11 sgRNA 1	TGCTGTAGATATCAGCCCAG
ABHD11 sgRNA 2	AAGATCTTGGCCCAGCAGAC
ABHD11 sgRNA 3	GCTGTGGCCAACGACGACGC
ABHD11 sgRNA 4	GCAGAAGGTCCTGCAGGTCC
LIAS sgRNA 1	TTAGGTTAAGACTACCTCCA
LIPT1 sgRNA 1	ATGCCTACCAATTACAACAG
LIPT1 sgRNA 2	GTAGCCTGCACATCCAGCTG
LIPT2 sgRNA 1	CAGGCGCACCAACCGAACGG
LIPT2 sgRNA 2	TATACGGCCGGGCTGCGCGG
MECR sgRNA 1	GCAGGGATTGACACCCAGGG
MECR sgRNA 2	GAAGTCCATCAACATCCTGT
OGDH sgRNA 1	CTGCTCTTACCTCCAGCCGA
OGDH sgRNA 2	TTCCTGTCCCCCGATGAAAG
PDHA1 sgRNA 1	GATGCAGACTGTACGCCGAA
PDHA1 sgRNA 2	AGGATGGGCTCAAATACTAC
PHD2 sgRNA 1	ATGCCGTGCTTGTTCATGCA
VHL sgRNA 1	GTGCCATCTCTCAATGTTGA
**Custom screen primers (TKO)**	
TKO Inner PCR forward	AATGATACGGCGACCACCGAGATCTACA CTCTCTTGTGGAAAGGACGAGGTACCG
TKO custom sequencing primer	ACACTCTCTTGTGGAAAGGACGAGGTACCG
**NEBuilder HiFi PCR primers to clone ABHD11 into pHRSIN lentiviral vector**	
Forward	CAGTCCTCCGACAGACTGAGTCGCCCGGGGG GGATCCGCCACCATGcgagccggccaacagcttgcaa
Reverse	CTTGCATGCCTGCAGGTCGACTCTAGAGTCGC GGCCGCttagaccaggaagcctcggatggcagc
**NEBuilder HiFi PCR primer to clone ABHD11 with C-terminal GFP tag into pHRSIN lentiviral vector**	
Reverse (for ABHD11)	CTTGCTCACgaccaggaagcctcggatggc
Forward (GFP)	gcttcctggtcGTGAGCAAGGGCGAGGAGCTG
Reverse (GFP)	GTCGACTCTAGAGTCGCGGCCGCttaCTTGTA CAGCTCGTCCATGCCGAG
**NEBuilder HiFi PCR primer to clone ABHD11 with C-terminal HA tag**	
Reverse	GTCGACTCTAGAGTCGCGGCCGCttaCT TGTACAGCTCGTCCATGCCGAG
**NEBuilder HiFi PCR primers to create silent mutations in ABHD11 sgRNA site 2 (mutations capitalised)**	
Forward	caaAatACtCgcAcaAcaAacaggccgtagggtg
Reverse	gtTtgTtgTgcGaGTatTttggcgatggagttgaagttag

**NEBuilder HiFi PCR primers to create S141A active site mutation in ABHD11**	
Forward	cacGCGatgggaggaaagacag
Reverse	catCGCgtggccaacgacgac
**NEBuilder HiFi PCR primers to create H296A active site mutation in ABHD11**	
Forward	ccgaacgctggcGCc
Reverse	cagcgtggatccagGCg
**Gibson Assembly PCR primers to clone ABHD11 into pCEFL 3xFLAG mCherry vector**	
Forward	GGAATTGGCGAAGCTTGGTACCGAGCTCGG ATCCGCCACCATGcgagccggccaacagcttgcaa
Reverse	CCGTCATGGTCTTTGTAGTCAGCCCGCTCG AGCGGCCGCCCgaccaggaagcctcggatggcagc
**QPCR primers**	
GAPDH Forward	ATGGGGAAGGTGAAGGTCG
GAPDH Reverse	CTCCACGACGTACTCAGCG
CAIX Forward	GCCGCCTTTCTGGAGGA
CAIX Reverse	TCTTCCAAGCGAGACAGCAA
VEGF Forward	TACCTCCACCATGCCAAGTG
VEGF Reverse	ATGATTCTGCCCTCCTCCTTC

List of sgRNA sequences used, and PCR primers used for genome-wide screen amplification and sequencing, and cloning of ABHD11 expression vectors.
