# Supplementary material for: ABHD11 maintains 2-oxoglutarate metabolism by preserving functional lipoylation of the 2-oxoglutarate dehydrogenase complex
Source: Nat Commun. 2020 Aug 13;11:4046. doi: 10.1038/s41467-020-17862-6 (PMC7426941; doi:10.1038/s41467-020-17862-6)
Supplement: Supplementary file 5 — Reporting Summary [file 41467_2020_17862_MOESM5_ESM.pdf]

nature research

Corresponding author(s): James A Nathan

Last updated by author(s): Jul 3, 2020

Reporting Summary

Nature Research wishes to improve the reproducibility of the work that we publish. This form provides structure for consistency and transparency in reporting. For further information on Nature Research policies, see Authors & Referees and the Editorial Policy Checklist.

Statistics

For all statistical analyses, confirm that the following items are present in the figure legend, table legend, main text, or Methods section.

n/a	Confirmed	
☐	☒	The exact sample size (n) for each experimental group/condition, given as a discrete number and unit of measurement
☐	☒	A statement on whether measurements were taken from distinct samples or whether the same sample was measured repeatedly
☐	☒	The statistical test(s) used AND whether they are one- or two-sided<br>*Only common tests should be described solely by name; describe more complex techniques in the Methods section.*
☒	☐	A description of all covariates tested
☐	☒	A description of any assumptions or corrections, such as tests of normality and adjustment for multiple comparisons
☐	☒	A full description of the statistical parameters including central tendency (e.g. means) or other basic estimates (e.g. regression coefficient) AND variation (e.g. standard deviation) or associated estimates of uncertainty (e.g. confidence intervals)
☐	☒	For null hypothesis testing, the test statistic (e.g. F, t, r) with confidence intervals, effect sizes, degrees of freedom and P value noted<br>*Give P values as exact values whenever suitable.*
☒	☐	For Bayesian analysis, information on the choice of priors and Markov chain Monte Carlo settings
☒	☐	For hierarchical and complex designs, identification of the appropriate level for tests and full reporting of outcomes
☐	☒	Estimates of effect sizes (e.g. Cohen's d, Pearson's r), indicating how they were calculated

Our web collection on statistics for biologists contains articles on many of the points above.

Software and code

Policy information about availability of computer code

Data collection	Flow cytometry: BD FACSDiva 8.3<br>QPCR: Quantstudio 1.3<br>Plate reader: CLARIOstar 5.60 R2<br>LC-MS metabolite carbon tracing: XCalibur 3 Qual Browser and XCalibur 3 Quan Browser (Thermo).<br>Seahorse: Wave 2.3<br>Gel filtration: Unicorn 6.3
Data analysis	Flow cytometry: FlowJo v10 (BD)<br>CRISPR screen: Reads trimmed with FASTX-toolkit (0.0.14), and aligned using Bowtie (1.2.2). Analysed with MAGeCK (0.5.5)<br>Immunoblot quantification: ImageJ (1.52d)<br>Microscopy and calculation of correlation coefficients: Zen Blue 2.3 (Zeiss)<br>Proteomics: PEAKS Studio 8.0. XCalibur 4 Quan Browser 4.0.27.13<br>Label-free quantitation of DLST PTMs: MaxQuant (version 1.6.6.0)<br>Modelling of ABHD11 structure and MTS prediction: Phyre2 (http://www.sbg.bio.ic.ac.uk/~phyre2/), PyMOL (2.3), MitoFates (http://mitf.cbrc.jp/MitoFates/)<br>Phylogenetic analysis and alignment of ABHD family members: Clustal Omega 1.2.4<br>Microsoft Excel for Mac version 16<br>Graphpad Prism version 8

For manuscripts utilizing custom algorithms or software that are central to the research but not yet described in published literature, software must be made available to editors/reviewers. We strongly encourage code deposition in a community repository (e.g. GitHub). See the Nature Research guidelines for submitting code & software for further information.

Data

Policy information about availability of data

All manuscripts must include a data availability statement. This statement should provide the following information, where applicable:

- Accession codes, unique identifiers, or web links for publicly available datasets
- A list of figures that have associated raw data
- A description of any restrictions on data availability

SgRNA read count tables from CRISPR/Cas9 genetic screens (Figure 1b,c) are shown in Supplementary Dataset 1. LC-MS metabolic profiling has been deposited in MetaboLights59 [www.ebi.ac.uk/metabolights/MTBLS1875]. The mass spectrometry proteomics data have been deposited to the ProteomeXchange Consortium via the PRIDE partner repository with the dataset identifier PXD020128 and the results and analysis are also available in Supplementary Dataset 2.

Field-specific reporting

Please select the one below that is the best fit for your research. If you are not sure, read the appropriate sections before making your selection.

☒ Life sciences ☐ Behavioural & social sciences ☐ Ecological, evolutionary & environmental sciences

For a reference copy of the document with all sections, see nature.com/documents/nr-reporting-summary-flat.pdf

Life sciences study design

All studies must disclose on these points even when the disclosure is negative.

Sample size	No sample-size calculation was performed. Based on pilot experiments and their variability, the number of experiments was determined.
Data exclusions	No data were excluded from the study
Replication	The CRISPR screen was independently performed twice, as displayed. Western blotting data were confirmed with more than 2 independent samples (specific details provided in the methods under Statistics and Reproducibility). Flow cytometry, Seahorse bioenergetics, in vitro OGDH activity, in vitro pNP esterase activity experiments were performed with at least two biologically independent experiments. The HIF-ODD hydroxylation assay figure summarises three independent experiments. All attempts at replication were successful. Biological and technical replicates of the LC-MS metabolite tracing and DLST PTM analysis were performed as described.
Randomization	Samples for LC-MS metabolite tracing were randomised in order to avoid bias in sample analyses due to machine drift. Flow cytometry experiments were analysed in random order. Other experiments were allocated into experimental groups in order of presentation, as systematic bias was not felt to affect these experiments.
Blinding	Samples for LC-MS metabolite tracing and DLST PTM analysis were submitted as coded samples and results analysed before sample group unblinded. Other samples were not blinded (felt to be less relevant as the major method of the project, the CRISPR screen, was unbiased, and blots were prepared in an order that allowed effective presentation), however key experiments (immunoblot of lipoylation with ABHD11 CRISPR or inhibition) were performed independently by three authors.

Reporting for specific materials, systems and methods

We require information from authors about some types of materials, experimental systems and methods used in many studies. Here, indicate whether each material, system or method listed is relevant to your study. If you are not sure if a list item applies to your research, read the appropriate section before selecting a response.

Materials & experimental systems

n/a	Involved in the study	
☐	☒	Antibodies
☐	☒	Eukaryotic cell lines
☒	☐	Palaeontology
☒	☐	Animals and other organisms
☒	☐	Human research participants
☒	☐	Clinical data

Methods

n/a	Involved in the study	
☒	☐	ChIP-seq
☐	☒	Flow cytometry
☒	☐	MRI-based neuroimaging

Antibodies

Antibodies used	Details of primary and secondary antibodies, and the dilutions used for immunoblot and microscopy, are described in Supplementary Table 2
Validation	5hmC: Validated for dot blot by manufacturer; references available on manufacturer website: https://www.activemotif.com/

catalog/details/39769
ABHD11: Validated by the authors by CRISPR/Cas9 knockout and by overexpression (for example Figure 1e-g in this manuscript)
B actin: Validated for immunoblot by manufacturer; references available on website: https://www.sigmaaldrich.com/catalog/product/sigma/a2228
Cytochrome C: Validated for immunoblot by manufacturer: https://www.abcam.com/cytochrome-c-antibody-37ba11-ab110325.html
DLD: Validated for immunoblot by manufacturer: https://www.genetex.com/Product/Detail/DLD-antibody/GTX101245
DLAT: Validated for immunoblot and IP by manufacturer: https://www.cellsignal.com/products/primary-antibodies/dlat-4a4-b6-c10-mouse-mab/12362
DLST (mouse): Validated for immunoblot and IP by manufacturer: https://www.abcam.com/dlst-antibody-9f4bd5-ab110306.html
DLST (rabbit): Validated for immunoblot and IP by manufacturer: https://www.cellsignal.com/products/primary-antibodies/dlst-d22b1-xp-rabbit-mab/11954
GFP: Validated for immunoblot by manufacturer: https://www.sigmaaldrich.com/catalog/product/roche/11814460001
H3: Validated for immunoblot by manufacturer; references available: https://www.cellsignal.com/products/primary-antibodies/histone-h3-d1h2-xp-rabbit-mab/4499
H3K4me3: Validated for immunoblot by manufacturer; references available: https://www.cellsignal.co.uk/products/primary-antibodies/tri-methyl-histone-h3-lys4-c42d8-rabbit-mab/9751
H3K9me3: Validated for immunoblot by manufacturer; references available: https://www.cellsignal.co.uk/products/primary-antibodiValidated for dot blot by manufacturer; references available on manufacturer website: es/tri-methyl-histone-h3-lys9-d4w1u-rabbit-mab/13969
HA: Validated for immunoblot by manufacturer: https://www.sigmaaldrich.com/catalog/product/roche/roahaha
HIF-1a: Validated for dot blot by manufacturer; references available on manufacturer website: https://www.bdbiosciences.com/us/applications/research/stem-cell-research/mesoderm-markers/human/purified-mouse-anti-human-hif-1-54hif-1/p/610959
Hydroxy-HIF-1a: Validated for dot blot by manufacturer; references available on manufacturer website: https://www.cellsignal.co.uk/products/primary-antibodies/hydroxy-hif-1a-pro564-d43b5-xp-rabbit-mab/3434
Lipoic acid: Validated for dot blot by manufacturer ("Does not bind lipoic acid modified by 4-HNE and other lipid peroxidation products"): https://www.merckmillipore.com/GB/en/product/Anti-Lipoic-Acid-Rabbit-pAb,EMD_BIO-437695
MECR: Validated for dot blot by manufacturer; references available on manufacturer website: https://www.thermofisher.com/antibody/product/Mecr-Antibody-Polyclonal/51027-2-AP
MFN2: Validated for immunoblot by manufacturer: https://www.abcam.com/mitofusin-2-antibody-6a8-ab56889.html
NDUFB8: Validated for immunoblot by manufacturer: https://www.abcam.com/ndufb8-antibody-20e9dh10c12-ab110242.html
OGDH: Validated for immunoblot by knockout by the authors (reference 4 in manuscript) and by manufacturer: https://www.sigmaaldrich.com/catalog/product/sigma/hpa020347?lang=en&region=GB
PHD2: Validated for immunoblot by manufacturer: https://www.novusbio.com/products/egln1-phd2-antibody_nb100-137

Eukaryotic cell lines

Policy information about cell lines

Cell line source(s)	HEK293T, HeLa, THP-1 and MCF-7 cells were a gift from Paul Lehner (Cambridge Institute for Therapeutic Immunology & Infectious Disease)<br>C2C12 cells were a gift from Michael Murphy (MRC Mitochondrial Biology Unit, Cambridge); originally obtained from ATCC (Cat#CRL-1772; RRID: CVCL_0188).
Authentication	All cells were authenticated by Eurofins as follows: genetic characteristics were determined by PCR-single-locus-technology. 21 independent PCR-systems Amelogenin, D3S1358, D1S1656, D6S1043, D13S317, Penta E, D16S539, D18S51, D2S1338, CSF1PO, Penta D, TH01, vWA, D21S11, D7S820, D5S818, TPOX, D8S1179, D12S391, D19S433 and FGA were investigated (Promega, PowerPlex 21 PCR Kit). In parallel, positive and negative controls were carried out yielding correct results.
Mycoplasma contamination	All cell lines tested negative for Mycoplasma contamination.
Commonly misidentified lines (See ICLAC register)	None

Flow Cytometry

Plots

Confirm that:

☒ The axis labels state the marker and fluorochrome used (e.g. CD4-FITC).

☒ The axis scales are clearly visible. Include numbers along axes only for bottom left plot of group (a 'group' is an analysis of identical markers).

☒ All plots are contour plots with outliers or pseudocolor plots.

☒ A numerical value for number of cells or percentage (with statistics) is provided.

Methodology

Sample preparation	For flow cytometric sorting for the CRISPR screen, HeLa cells were suspended using Trypsin-EDTA, centrifuged at 800g, washed in cold PBS, centrifuged again and resuspended in PBS containing 2% fetal calf serum and 10 mM HEPES.
Instrument	Cells were sorted using a BD Influx cell sorter

Software	BD FACS Sortware 1.2.0.142. Analysed using FlowJo v10 (BD)
Cell population abundance	As displayed in figure 1a (x axis is GFP, y axis is SSC), at the first sort approximately 1% of cells above one log10 unit of GFP fluorescence above the mode of the main population were selected. At the first sort this represented 30% of events.
Gating strategy	Live cells were selected by drawing a gate around the modal population as seen with FSC against SSC (for the first sort of the Brunello screen, for example, this gate included 97% of events). Single cells were selected by drawing a gate around the modal population as seen with trigger pulse width against FSC (for the first sort of the Brunello screen this gate included 95% of the parent population).

☒ Tick this box to confirm that a figure exemplifying the gating strategy is provided in the Supplementary Information.
