# Supplementary material for: ABHD11 maintains 2-oxoglutarate metabolism by preserving functional lipoylation of the 2-oxoglutarate dehydrogenase complex
Source: Nat Commun. 2020 Aug 13;11:4046. doi: 10.1038/s41467-020-17862-6 (PMC7426941; doi:10.1038/s41467-020-17862-6)
Supplement: Supplementary file 2 — Peer Review File [file 41467_2020_17862_MOESM2_ESM.docx]

Peer Review Information

**Journal:** Nature Communications

**Manuscript title:** ABHD11 regulates 2-oxoglutarate abundance by protecting mitochondrial lipoylated proteins from lipid peroxidation damage

**Corresponding author name(s):** Dr James Nathan

**Editorial notes:**

**Transferred manuscripts**

This manuscript has been previously reviewed at another journal that is not operating a transparent peer review scheme. This document only contains reviewer comments and rebuttal letters for versions considered at *Nature Communications*.

**Reviewer comments & decisions:**

**Reviewer comments, first version:**

Reviewers' comments:

Reviewer #1 (Remarks to the Author):

The manuscript by Bailey and colleagues uses unbiased screening for genes that regulate HIF stability to discover novel regulators of 2OG and 2HG that can affect the hydroxylation and stabilization of HIF1a. Through these approaches, the authors identify a poorly characterized protein, ABHD11, as an enzyme with serine hydrolyase activity that controls activity of the OGDH complex in the TCA cycle. Cells lacking ABHD11 have reduced lipoylation of OGDHc, consistent with reduced activity. The authors suggest a model wherein ABHD11 antagonizes oxidation of lipolyated forms of the OGDH subunit DLST to maintain OGDH activity.

This is a very interesting manuscript with many well-done experiments and an intriguing new model for regulation of mitochondrial metabolism. A particular strength of the manuscript is the combination of genetics, metabolism, and biochemistry to pinpoint altered regulation of OGDH following manipulation of ABHD11 expression. Given the fundamental advance of this paper—a new mode of regulation of OGDH—this work is likely to be of great interest to readers of Nature Communications. A few major points should be addressed to further test this model and consider alternative explanations:

1. The authors state in the discussion that ABDH11 represents “the first example of a mitochondrial specific response that maintains TCA cycle integrity by counteracting the damage caused by lipid peroxidation products,” but these conclusions are based largely on loss of function studies and the authors don’t directly show whether ABDH11 can actively counteract products of lipid peroxidation. Figure S5 shows overexpression of ABDH11 but this doesn’t seem to reduce damage caused by 5HNE relative to control cells (although admittedly these are on separate gels). Could the authors use their recombinant enzyme or their overexpression lines to test whether ABDH11 does indeed counteract damage caused by lipid peroxidation and/or increase functional lipoylation of DLST? Some gain of function assays testing the role of ABDH11 in removal of these products is warranted to support this claim. Alternatively, the authors would need to consider the possibility that ABDH11 acts indirectly to regulate DLST lipoylation or that adaptation to loss of ABDH11 function supports an oxidative environment that decreases functional DLST lipoylation.

2. Relatedly, as ROS can both inhibit OGDHc and stabilize HIF, an alternate explanation for the observed phenotypes—beyond reversal of oxidative damage of the lipoyl modification of DLST—is that ABDH11 regulates ROS/redox stress. Does ABDH11 affect ROS production and/or do antioxidants affect any of the consequences of ABDH11 loss independently of modulating DLST lipoylation?

3. It’s not clear why the increase in 2OG following ABDH11 KO/OGDH inhibition causes an increase in L2HG. Does ABDH11 KO induce a change in cellular redox that supports reduction of 2OG?

Minor points
1. This is beyond the scope of the study current study, but do the authors have any insight into the potential enzymatic function of ABDH11?
2. Could the authors clarify in the methods whether the two biological replicates shown for each LC-MS experiment represent the same cell line analyzed on different days or two different cell lines? (Either is of course totally fine, it would just be helpful to have this point clarified.)
3. In Fig. S1g, the label lanes are shifted.
4. This is a minor quibble, but Fig. S1k nicely shows that ABHD11 does not affect levels of methylation on certain histone lysine residues. Because methylation is a reflection of methyltransferase activity, nucleosome turnover and demethylation, steady-state methylation levels don’t necessarily merely reflect KDM activity. The text on p. 6 should be modified to reflect the data, which show differing effects on marks controlled by 2-OG DD enzymes, but cannot per se claim that KDM activity is not affected.


Reviewer #2 (Remarks to the Author):

The manuscript by Bailey et al described a potential role of ABHD11 in regulating 2-oxoglutarate abundance by protecting mitochondrial lipoylated proteins from lipid peroxidation damage. ABDH11 was identified in a CRISPR Cas9 genome-wide mutagenesis screen for genetic determinants of 2-OG levels. They provided convincing evidence that ABDH11 is tightly linked to mitochondrial 2-OG metabolism and 2-OG DD function. Furthermore, loss or inhibition of ABDH11 leads to a rapid increase in 2-OG levels by impairing lipoylation of the 2-OG dehydrogenase complex (OGDHc) – the rate limiting step for mitochondrial 2-OG metabolism. They later attempted to show that ABHD11 protects the catalytic lipoyl domain from lipid peroxidation products formed by oxidative damage. While the study appears interesting, the claim that lipid peroxidation products, such as 4-HNE, play a significantly role in this mechanism is rather unsubstantiated. The major concerns for this conclusions are at least three folds. First of all, the author used superhigh concentrations of 4-HNE in their studies, from 0.1 to 5 mM, which is biologically irrelevant. Previous studies showed that the endogenous concentration of these active lipid aldehydes are the range of nM to sub-microM (PMC5318235, PMC5058421, PMC4151707 PMID: 16300372). Incubation of cells with concentration of 4-HNE over 50 uM leads to apoptosis. For the cell lines used in this studies, the levels of 4-HNE and other lipid aldehdydes are even lower. Such a high concentration of 4-HNE will lead to the depletion of cellular GSH and massive alterations of redox environment of the cell, which may have nothing to do with the proposed mechanism in this studies. Secondly, the authors never showed the evidence of 4-HNE adducts that they proposed in Figure 4, instead using some indirect evidence of lipoylation of DLST and DLAT. Thirdly, lipid peroxidation of a slow process whose kinetics does not appear to match with the HIF response (PMID: 28456642; PMID: 25598486). Last but not least, the author are recommended to measure the redox alterations in cells, such as hydrogen peroxides, super oxides, GSH etc.

Minor points:

1. In the metabolic flux analysis (Figure 2b-f, Figure S3a-g), the ion intensities were used. It remains unclear how they accounted for the ionization efficiency (MS responses) for different metabolites. Did they take account the natural abundances?


Reviewer #3 (Remarks to the Author):

Summary:
2-oxoglutarate (2-OG or a-ketoglutarate) relates mitochondrial metabolism other cell functions by modulating the activity of 2-OG dependent dioxygenases (2-OG DDs) involved in the response to hypoxia and DNA/histone modifications. To find genes involved in 2-OG metabolism, the authors built a fluorescent HIF reporter that encodes consensus HIF responsive elements in triplicate driving expression of GFP fused to the oxygen and 2-OG sensitive region of HIF1a. The reporter stability is dependent on 2-OG DD activity of the prolyl hydroxylases (PHDs or EGLNs), and is responsive to treatment of the PHD inhibitor dimethyloxalylglycine (DMOG), cell permeable 2-OG (dimethyl 2-OG, DM, 2-OG) or incubation in 1% oxygen. Two genome-wide CRISPR knockout screens were performed using different libraries to identify genes important for GFP fluorescence. Both screens identified genes involved in the canonical pathway for HIF stability (VHL, EGLN1, PHD2) and 2-OG metabolism (OGDHc components, lipoic acid synthesis pathway), as well as genes involved in the mTOR pathway, intracellular iron metabolism, and transcriptional regulation. In addition, the uncharacterized a/b hydrolase ABHD11 was strongly enriched.

The authors focus the rest of the manuscript on ABHD11 and show that it is a mitochondrial hydrolase that signals changes in mitochondrial 2-OG metabolism to 2-OG DD. ABHD11 loss or inhibition drives a rapid increase in 2-OG levels by impairing lipoylation of the 2-OG dehydrogenase complex (OGDHc), which is the rate limiting step for mitochondrial 2-OG metabolism. Rather than facilitating lipoate conjugation, ABHD11 protects the catalytic lipoyl domain from lipid peroxidation products formed by oxidative damage. These results demonstrate a requirement for a lipoyl repair pathway in human cells and highlights how redox sensitivity of lipoylation modulates 2-OG metabolism.


Thoughts:
The authors present a fascinating study on a new gene involved in 2-OG metabolism, an area of research that would benefit from some genetics and a better understanding of the role of 2-OG DDs in disease. The findings in the paper are presented clearly and in a logical fashion. Although the model presented may need to be further validated in additional models to be generalizable, the clear finding that ABHD11 is involved in regulation of OGDHc provides a deeper understanding of the role of lipoylation in metabolism.

Specific comments:
1. Fig 1b – several genes have the same -log10(p-value) using the TKO library including EGLN1, IREB2, TSC1, VHL, AP2M1, TSC2 and ZCCHC11. Please explain this result further.
2. Loss of ABHD11 leads to an increase in HIF1a in multiple cell lines (e.g. Hela, MCF7, HepG2). How generalizable are these findings to other outcomes including VEGF and CA9 induction?
3. There are >18 different ABHD genes encoded in the human genome, and many of these are expressed in commonly used cell lines. ABHD11 has an important paralog called EPHX3/ABHD9. What is special about ABHD11 compared with the other ABHD encoding genes? Have the authors aligned all of the different ABHD protein sequences to see how ABHD11 is unique or similar from other ABHD proteins. And does this provide insight into why ABHD11 is unique?
4. ABHD11 is also known as WBSCR21 and is associated with Williams Beuren Syndrome, a rare disease associated with developmental delays in cognition. Although it is outside the scope of this study to examine whether patient samples with Williams Syndrome exhibit the same molecular deficits in lipoylation as described in this study an ABHD11 perturbation, it would be interesting to have a few sentences in the discussion related to how the findings in the paper relate to this particular syndrome.


Reviewer #4 (Remarks to the Author):

In this study, Bailey et al. screened for genes involved in 2-OG metabolism using a HIF reporter system which is activated by 2-OG – dependent dioxygenases. Among others, they identified the uncharacterized esterase ABHD11 as potential regulator of HIF-1a expression and 2-OG metabolism.

Further experiments revealed that ABHD11 loss was associated with HIF-1a accumulation and complementation of ABHD11 reduced HIF-1a levels. This was due to stabilization of HIF-1a in its non-hydroxylated form.

Metabolic labeling experiments revealed that loss of ABHD11 reduces OGDH activity and increases 2-OG levels, and impairs oxygen consumption. 2-OG accumulation was associated with L-2-HG accumulation, possibly responsible for the decreased 2-OG DD activity.

Loss of ABHD11 reduced lipoylation DLST subunit of the OGDHc implicating that ABHD11 plays a functional role in lipoylation. This is dependent on an active enzyme, since mutants lacking the active serine (S141A) or the acyltransferase motif (H296A) did not reconstitute lipoylation and a specific inhibitor mimicked the effect of ABHD11 deletion.
Finally, authors investigated whether the loss of DLST lipoylation in ABHD11 deficient cells may due to the formation of lipoyl adducts. Experiments indicate that ABHD11 prevents lipoyl adduct formation with lipid peroxidation products.

In general the paper is interesting and novel and links 2-OG metabolism with the orphan hydrolase ABHD11. However, some additional experiments would substantially improve the manuscript
Major:
1. ABHD11 may directly or indirectly regulate OGDHc lipoylation. Microscopic studies clearly revealed that ABHD11-GFP localized to mitochondria. To support the hypothesis that ABHD11 directly influences OGDH lipoylation, it is essential to show that ABHD11 is a matrix protein. However, the protease protection assay shown is not convincing, since there is not dose-effect of proteinase K. Furthermore, the IMS marker cytochrome C was not degraded.

Since these data are essential for the conclusion that ABHD11 directly influences OGDHc lipoylation, these data should be transferred to the main manuscript and experiments should be repeated. The localization of ABHD11 should be confirmed using a second method (e.g. digitonin extraction, super resolution microscopy…). How were mitoblasts isolated?

2. Authors propose that ABHD11 prevents lipoyl adduct formation with lipid peroxidation products. However, this is just shown by reduced detection in immunoblots. Authors screened for several DLST intermediates which did not account for the peptide loss. Did they also screen for the 4-HNE adduct? Is this adduct detectable in ABHD11 knockout cells and wild-type with and without HNE treatment.
3. ABHD11 cells seem to have a severe defect in mitochondrial function. Do these cells show normal growth. Is ABHD11-deficiency associated with increased lactate production?
4. The discussion section is very short. I would be interesting to discuss why ABHD11 activity is required for this process. Furthermore, ABHD11 is also among the genes deleted in Williams-Beuren Syndrome. Are data about mitochondrial function in this disease available?
5. Statistical analysis is not shown for all experiments (e.g. Fig.2h)

Minor:
1. The text is confusing due to the high number of abbreviations. Try to reduce abbreviation in the text and provide full name for all abbreviations and an abbreviation list
2. labels of Fig.S1g are displaced
3. use same abbrevations in text and figures (e.g. figure 1i)

**Author rebuttal, first version:**

Reviewer #1 (Remarks to the Author):

The manuscript by Bailey and colleagues uses unbiased screening for genes that regulate HIF stability to discover novel regulators of 2OG and 2HG that can affect the hydroxylation and stabilization of HIF1a. Through these approaches, the authors identify a poorly characterized protein, ABHD11, as an enzyme with serine hydrolyase activity that controls activity of the OGDH complex in the TCA cycle. Cells lacking ABHD11 have reduced lipoylation of OGDHc, consistent with reduced activity. The authors suggest a model wherein ABHD11 antagonizes oxidation of lipolyated forms of the OGDH subunit DLST to maintain OGDH activity.

This is a very interesting manuscript with many well-done experiments and an intriguing new model for regulation of mitochondrial metabolism. A particular strength of the manuscript is the combination of genetics, metabolism, and biochemistry to pinpoint altered regulation of OGDH following manipulation of ABHD11 expression. Given the fundamental advance of this paper—a new mode of regulation of OGDH—this work is likely to be of great interest to readers of Nature Communications. A few major points should be addressed to further test this model and consider alternative explanations:

We are pleased that this reviewer found the manuscript interesting, and notes the fundamental advance and interest to readers of Nature Communications.

1. The authors state in the discussion that ABDH11 represents “the first example of a mitochondrial specific response that maintains TCA cycle integrity by counteracting the damage caused by lipid peroxidation products,” but these conclusions are based largely on loss of function studies and the authors don’t directly show whether ABDH11 can actively counteract products of lipid peroxidation. Figure S5 shows overexpression of ABDH11 but this doesn’t seem to reduce damage caused by 5HNE relative to control cells (although admittedly these are on separate gels). Could the authors use their recombinant enzyme or their overexpression lines to test whether ABDH11 does indeed counteract damage caused by lipid peroxidation and/or increase functional lipoylation of DLST? Some gain of function assays testing the role of ABDH11 in removal of these products is warranted to support this claim. Alternatively, the authors would need to consider the possibility that ABDH11 acts indirectly to regulate DLST lipoylation or that adaptation to loss of ABDH11 function supports an oxidative environment that decreases functional DLST lipoylation.

Overexpression of ABHD11 does not increase detectable lipoylation, and the level seems to be tightly regulated. We only observe a difference in functional lipoylation when the overexpressed ABHD11 mutants compete with endogenous ABHD11. We have clarified this in the revised text (page 15).

We tried using recombinant ABHD11 on immunoprecipitated DLST but this approach was technically challenging, as observing the loss of lipoyl adducts by mass spectrometry was not successful, and functional lipoylation is not restored as the lipoyl conjugation machinery is not active in the immunoprecipitated samples. We also generated lipoyl-DLST peptides and determined if recombinant ABHD11 cleaved the lipoyl domain, as previously reported for SIRT4 (PMID:25525879). No cleavage was observed (see figure below). Commercial attempts to generate 4HNE-lipoyl-DLST were not successful, so this is technically not possible to pursue.

We have included a discussion regarding the overexpression of ABHD11, the technical limitations of the experiments, and acknowledgement that indirect regulation of DLST is possible in the revised text (page 16/17). However, the association of ABHD11 with the OGDHc complex is consistent with a direct enzymatic action, as is the focal defect in 2-OG metabolism.

**Figure R1**: HPLC-based assay of ABHD11 hydrolysis activity using ABHD11-FLAG against peptides derived from DLST. (**a**) Schematic of the experiment. Peptides were synthesised containing residues 105-114 of human DLST, with or without lipoylation of the lysine residue. Peptides were incubated at 37°C for 30 minutes with human ABHD11-FLAG (same reaction conditions as used in **Figure 3i**), the reaction was quenched with formic acid to a final concentration of 0.5%, and analysed with a Thermo Vanquish high performance liquid chromatography (HPLC) system with an Acclaim Pepmax RSLC column (10 minute gradient, solution A: water + 0.1% formic acid, solution B: acetonitrile + 0.1% formic acid; UV detector at 280 nm). (**b**-**e**) UV chromatograms of absorbance at 280 nm (AU) on y axis against time in minutes on x axis. (**b**) Enzyme and vehicle-only control. (**c**) DLST peptide (unlipoylated) positive control. (**d, e**) DLST-lipoyl peptide incubated with wildtype ABHD11 (**d**) or the S141 mutant (**e**), showing no formation of unlipoylated DLST.

2. Relatedly, as ROS can both inhibit OGDHc and stabilize HIF, an alternate explanation for the observed phenotypes—beyond reversal of oxidative damage of the lipoyl modification of DLST—is that ABDH11 regulates ROS/redox stress. Does ABDH11 affect ROS production and/or do antioxidants affect any of the consequences of ABDH11 loss independently of modulating DLST lipoylation?

We were keen to explore the involvement of ROS further, as suggested. We used MitoSOX Red as a marker for mitochondrial ROS and measured the effect of OGDH, LIAS or ABHD11 depletion on MitoSOX levels by flow cytometry. Low concentrations of Antimycin A were used as a control to block the electron transport chain and generate mitochondrial ROS (**New Figure S8c-e**). OGDH, LIAS or ABHD11 loss led to a small change in MitoSOX Red levels, but this was not sufficient to activate the HIF reporter. We also tested whether ABHD11 inhibition with ML226 altered ROS and no change in MitoSOX Red levels were observed (**New Figure S8e).** Therefore, alterations in mitochondrial ROS are unlikely to be responsible for our findings.

The involvement of cellular ROS in altering HIF-1α levels is controversial. ROS and ROS scavengers have been shown to alter HIF-1α levels, but we do not observe activation of our HIF reporter with mitochondrial ROS (**New Figure S8e)**. Our findings are consistent with 2-OG and the redox environment promoting L-2-HG formation leading to PHD2 inhibition, which is well supported in the literature by several groups (Oldham et al Cell Met 2015, Intlekofer et al Cell Met 2015, Burr et al Cell Met 2017 and Nadtochiy et al JBC 2016).

3. It’s not clear why the increase in 2OG following ABDH11 KO/OGDH inhibition causes an increase in L2HG. Does ABDH11 KO induce a change in cellular redox that supports reduction of 2OG?

We should have clarified this point. We and other groups have shown that disrupting OGDHc function, or promoting an acidic environment leads to spurious activity of LHDA, MDH1 and MDH2 in reducing 2-OG to L-2-HG (Oldham et al Cell Met 2015, Intlekofer et al Cell Met 2015, Burr et al Cell Met 2017 and Nadtochiy et al JBC 2016). The ability of LDHA inhibition to overcome this is entirely consistent with these previous published findings. We have made this clearer in the text (page 8).

Minor points

1. This is beyond the scope of the study current study, but do the authors have any insight into the potential enzymatic function of ABDH11?

We hypothesise that ABHD11 removes lipoyl conjugates that are formed through the reactive thiols, but how this enzymatically occurs is not yet clear and forms a major component of our ongoing studies. As mentioned, our attempts to study the enzymology in vitro with modified peptides has not been successful. We are pursuing alternative approaches, including lipidomics, chemically modified lipoyl probes and other lipoyl adducts.

2. Could the authors clarify in the methods whether the two biological replicates shown for each LC-MS experiment represent the same cell line analyzed on different days or two different cell lines? (Either is of course totally fine, it would just be helpful to have this point clarified.)

Thank you for raising this and we should have made this clearer. Two independent lentiviral transductions of the same cell line, done on different days. This has been clarified in the methods.

3. In Fig. S1g, the label lanes are shifted.

This has been corrected.

4. This is a minor quibble, but Fig. S1k nicely shows that ABHD11 does not affect levels of methylation on certain histone lysine residues. Because methylation is a reflection of methyltransferase activity, nucleosome turnover and demethylation, steady-state methylation levels don’t necessarily merely reflect KDM activity. The text on p. 6 should be modified to reflect the data, which show differing effects on marks controlled by 2-OG DD enzymes, but cannot per se claim that KDM activity is not affected.

Thank you for raising this important point. We have altered the text to reflect this.

Reviewer #2 (Remarks to the Author):

The manuscript by Bailey et al described a potential role of ABHD11 in regulating 2-oxoglutarate abundance by protecting mitochondrial lipoylated proteins from lipid peroxidation damage. ABDH11 was identified in a  CRISPR Cas9 genome-wide mutagenesis screen for genetic determinants of 2-OG levels. They provided convincing evidence that ABDH11 is tightly linked to mitochondrial 2-OG metabolism and 2-OG DD function. Furthermore, loss or inhibition of ABDH11 leads to a rapid increase in 2-OG levels by impairing lipoylation of the 2-OG dehydrogenase complex (OGDHc) – the rate limiting step for mitochondrial 2-OG metabolism.

We thank the reviewer for the appreciation that our studies provide convincing evidence that ABHD11 is tightly linked to mitochondrial 2-OG metabolism.

They later attempted to show that ABHD11 protects the catalytic lipoyl domain from lipid peroxidation products formed by oxidative damage. While the study appears interesting, the claim that lipid peroxidation products, such as 4-HNE, play a significantly role in this mechanism is rather unsubstantiated. The major concerns for this conclusions are at least three folds. First of all, the author used superhigh concentrations of 4-HNE in their studies, from 0.1 to 5 mM, which is biologically irrelevant. Previous studies showed that the endogenous concentration of these active lipid aldehydes are the range of nM to sub-microM (PMC5318235, PMC5058421, PMC4151707 PMID: 16300372). Incubation of cells with concentration of 4-HNE over 50 uM leads to apoptosis. For the cell lines used in this studies, the levels of 4-HNE and other lipid aldehdydes are even lower. Such a high concentration of 4-HNE will lead to the depletion of cellular GSH and massive alterations of redox environment of the cell, which may have nothing to do with the proposed mechanism in this studies.

A number of important points are raised that we were keen to address. The concentration of 4-HNE is higher than in vitro studies cited by this reviewer, but are in keeping with other published reports in cells (e.g. PMID: 27620528), where similar findings to ours were observed. In addition, our 4-HNE treatments were for 90 min, as opposed to 24 hr (PMID 16300372). Differences in the experimental design may also account the discrepancies in concentrations. Our studies on cell lysates and immunoprecipitated DLST differ from 4-HNE treatment of recombinant proteins, where higher concentrations of 4-HNE are likely to be required, as it will react with all cysteine residues. Our cell-based assays were conducted with Opti-MEM, which is based on MEM, and contains 0.1mM cysteine, which would react with 4-HNE.

To further explore the role of 4-HNE and the concerns raised over the concentrations used we have repeated the experiments using lower concentrations of 4-HNE in media without L-cysteine for 90 min. We now observe a loss of DLST lipoylation with 40 µM 4-HNE, and complete loss of both DLST and DLAT lipoylation with 200 µM (**New Figure 5f**). ABHD11 depletion or inhibition increases the sensitivity of cells to 4-HNE treatment, with no detectable DLST lipoylation at 20 µM 4-HNE, and DLAT lipoylation is impaired with 80 µM 4-HNE. Therefore, our findings are consistent with biologically relevant concentrations of 4-HNE.

We have included both the original experiments and the new L-cysteine deficient media experiments in the revised manuscript to illustrate the importance of the technical considerations of the assay. We hope that this will be both helpful and of interest to the scientific community.

Secondly, the authors never showed the evidence of 4-HNE adducts that they proposed in Figure 4, instead using some indirect evidence of lipoylation of DLST and DLAT.

We have used a number of techniques to directly measure 4-HNE lipoyl adducts and have not been able to detect them using extensive mass spectrometry studies (**Supplemental File 2**). We have also taken alternative approaches, including treating immunoprecipitated DLST with 4-HNE, but this again prevents detection of the protein peptides. This is likely due to a number of cysteines being modified and the complex nature of the immunoprecipitated DSLT sample preventing detection by mass spectrometry. These findings highlight the technical limitations of mass spectrometry for complex protein samples, as 4-HNE DLST treatment alone cannot be detected. This is in contrast to several of the studies referred to by this reviewer, where 4-HNE peroxidation products are measured on recombinant proteins, which is technically not possible in our system, as DLST is only lipoylated endogenously in the mitochondria of mammalian cells, and not in bacteria (see also comments to Reviewer 1). Despite these limitations, our data clearly show that the DLST lipoyl peptide is modified when ADBH11 is depleted (**Figure 5**), and this is a key finding.

An aspect that we should have made clearer is that other adducts aside from 4-HNE are possible, and we cannot exclude these in our studies. Our ongoing work is to establish an *in vitro* system that will allow us to determine the enzymatic activity of ABHD11 against lipoyl adducts, but this is beyond the scope of this current study.

In summary, we acknowledge that other possibilities aside from 4-HNE adducts may occur, and we have substantially modified the title, abstract, manuscript and concluding model to better reflect this. However, it is important to emphasise that our main findings relate to the requirement of ABHD11 for functional lipoylation of the OGDHc. In contrast to other mitochondrial enzymes linked to lipoylation, ABHD11 is not required for initial conjugation, but serves a previously unappreciated role in the maintenance of the lipoyl moiety on DLST. These findings are a significant advance in our understanding of lipoylation and ABHD11 as an orphan mitochondrial hydrolase.

Thirdly, lipid peroxidation of a slow process whose kinetics does not appear to match with the HIF response (PMID: 28456642; PMID: 25598486).

HIF-1α is stabilised following the accumulation of L-2-HG which is formed when OGDHc activity is impaired and is therefore not stabilised immediately, as observed with PHD inhibitors. The kinetics of lipid peroxidation are consistent with the stabilisation of HIF-1α observed in our studies.

Last but not least, the author are recommended to measure the redox alterations in cells, such as hydrogen peroxides, super oxides, GSH etc.

We now include data demonstrating that GSH is not altered following ABHD11 depletion, and does therefore not account for our observations. We have also measured changes in mitochondrial ROS following ABHD11, OGDH or LIAS depletion (see **New Figure S8** and also comments to Reviewer 1).

Minor points:

1. In the metabolic flux analysis (Figure 2b-f, Figure S3a-g), the ion intensities were used. It remains unclear how they accounted for the ionization efficiency (MS responses) for different metabolites. Did they take account the natural abundances?

All data accounted for natural abundances. Ionization efficiency is metabolite-dependent, and thus any peak area comparisons between different metabolite pools would be difficult without absolute quantification. However, we compare effects of ABHD11 KO to WT for each metabolite separately (e.g. 2-OG levels in ABHD11 KO compared to WT).

Reviewer #3 (Remarks to the Author):

Summary:

2-oxoglutarate (2-OG or a-ketoglutarate) relates mitochondrial metabolism other cell functions by modulating the activity of 2-OG dependent dioxygenases (2-OG DDs) involved in the response to hypoxia and DNA/histone modifications. To find genes involved in 2-OG metabolism, the authors built a fluorescent HIF reporter that encodes consensus HIF responsive elements in triplicate driving expression of GFP fused to the oxygen and 2-OG sensitive region of HIF1a. The reporter stability is dependent on 2-OG DD activity of the prolyl hydroxylases (PHDs or EGLNs), and is responsive to treatment of the PHD inhibitor dimethyloxalylglycine (DMOG), cell permeable 2-OG (dimethyl 2-OG, DM, 2-OG) or incubation in 1% oxygen. Two genome-wide CRISPR knockout screens were performed using different libraries to identify genes important for GFP fluorescence. Both screens identified genes involved in the canonical pathway for HIF stability (VHL, EGLN1, PHD2) and 2-OG metabolism (OGDHc components, lipoic acid synthesis pathway), as well as genes involved in the mTOR pathway, intracellular iron metabolism, and transcriptional regulation. In addition, the uncharacterized a/b hydrolase ABHD11 was strongly enriched.

The authors focus the rest of the manuscript on ABHD11 and show that it is a mitochondrial hydrolase that signals changes in mitochondrial 2-OG metabolism to 2-OG DD. ABHD11 loss or inhibition drives a rapid increase in 2-OG levels by impairing lipoylation of the 2-OG dehydrogenase complex (OGDHc), which is the rate limiting step for mitochondrial 2-OG metabolism. Rather than facilitating lipoate conjugation, ABHD11 protects the catalytic lipoyl domain from lipid peroxidation products formed by oxidative damage. These results demonstrate a requirement for a lipoyl repair pathway in human cells and highlights how redox sensitivity of lipoylation modulates 2-OG metabolism.

Thoughts:

The authors present a fascinating study on a new gene involved in 2-OG metabolism, an area of research that would benefit from some genetics and a better understanding of the role of 2-OG DDs in disease. The findings in the paper are presented clearly and in a logical fashion. Although the model presented may need to be further validated in additional models to be generalizable, the clear finding that ABHD11 is involved in regulation of OGDHc provides a deeper understanding of the role of lipoylation in metabolism.

We thank this reviewer for these supportive comments.

Specific comments:

1.	Fig 1b – several genes have the same -log10(p-value) using the TKO library including EGLN1, IREB2, TSC1, VHL, AP2M1, TSC2 and ZCCHC11. Please explain this result further.

Our screening results were analysed using the MAGeCK algorithm (PMID 25476604). For ease of reproducibility we used the default settings. The p values generated are calculated using permutation, and thus there is a minimum p value achievable for genes most strongly enriched by the screen. We have noticed this phenomenon with multiple other screens from our group and others (for example, PMID 28167502), and indeed it is visible in the example data used in the documentation of the MAGeCK algorithm:

<https://sourceforge.net/p/mageck/wiki/demo/#step-5-compare-samples-using-mageck-test-subcommand>

2.	Loss of ABHD11 leads to an increase in HIF1a in multiple cell lines (e.g. Hela, MCF7, HepG2). How generalizable are these findings to other outcomes including VEGF and CA9 induction?

In all cell lines where HIF-1α is increased, we have observed a similar increase in carbonic anhydrase 9 induction. This is dependent on the increase in 2-OG and that may still vary according to the cell line used. This is an area that we plan to explore further.

3.	There are >18 different ABHD genes encoded in the human genome, and many of these are expressed in commonly used cell lines. ABHD11 has an important paralog called EPHX3/ABHD9. What is special about ABHD11 compared with the other ABHD encoding genes? Have the authors aligned all of the different ABHD protein sequences to see how ABHD11 is unique or similar from other ABHD proteins. And does this provide insight into why ABHD11 is unique?

Thank you for this helpful suggestion. We have now included alignments of the different ABHDs that show that ABHD11 shares many similarities with other family members (**New Figure S2**). However, the alpha-beta hydrolase fold is common and present in many enzymes. ABHD9 is a paralogue of ABHD11 but does not localise to the mitochondria, and has an aspartic acid nucleophile instead of serine. Another interesting paralogue is ABHD10, the only other ABHD family member known to localise to mitochondria, which was recently identified as acyl thioesterase (PMID: 31740833). However, *in vitro* assays to develop ABHD inhibitors do show that there is high specificity within the group, and ABHD11 inhibition does not alter the activity of either ABHD9 or ABHD10 (PMID: 23658953).

4.	ABHD11 is also known as WBSCR21 and is associated with Williams Beuren Syndrome, a rare disease associated with developmental delays in cognition. Although it is outside the scope of this study to examine whether patient samples with Williams Syndrome exhibit the same molecular deficits in lipoylation as described in this study an ABHD11 perturbation, it would be interesting to have a few sentences in the discussion related to how the findings in the paper relate to this particular syndrome.

We have now included a discussion of ABHD11 in relation to Williams-Beuren Syndrome. This rare syndrome is characterised by a chromomosal deletion in 7q11.23. The main gene responsible for its soft tissue and metabolic phenotypes is thought to be ELN1, rather than ABHD11. Whether haploinsufficiency of ABHD11 causes disturbances in 2-oxoglutarate metabolism forms part of our ongoing studies in animal models.

Reviewer #4 (Remarks to the Author):

In this study, Bailey et al. screened for genes involved in 2-OG metabolism using a HIF reporter system which is activated by 2-OG – dependent dioxygenases. Among others, they identified the uncharacterized esterase ABHD11 as potential regulator of HIF-1a expression and 2-OG metabolism.

Further experiments revealed that ABHD11 loss was associated with HIF-1a accumulation and complementation of ABHD11 reduced HIF-1a levels. This was due to stabilization of HIF-1a in its non-hydroxylated form.

Metabolic labeling experiments revealed that loss of ABHD11 reduces OGDH activity and increases 2-OG levels, and impairs oxygen consumption. 2-OG accumulation was associated with L-2-HG accumulation, possibly responsible for the decreased 2-OG DD activity.

Loss of ABHD11 reduced lipoylation DLST subunit of the OGDHc implicating that ABHD11 plays a functional role in lipoylation. This is dependent on an active enzyme, since mutants lacking the active serine (S141A) or the acyltransferase motif (H296A) did not reconstitute lipoylation and a specific inhibitor mimicked the effect of ABHD11 deletion.

Finally, authors investigated whether the loss of DLST lipoylation in ABHD11 deficient cells may due to the formation of lipoyl adducts. Experiments indicate that ABHD11 prevents lipoyl adduct formation with lipid peroxidation products.

In general the paper is interesting and novel and links 2-OG metabolism with the orphan hydrolase ABHD11. However, some additional experiments would substantially improve the manuscript.

Major:

1. ABHD11 may directly or indirectly regulate OGDHc lipoylation. Microscopic studies clearly revealed that ABHD11-GFP localized to mitochondria. To support the hypothesis that ABHD11 directly influences OGDH lipoylation, it is essential to show that ABHD11 is a matrix protein. However, the protease protection assay shown is not convincing, since there is not dose-effect of proteinase K. Furthermore, the IMS marker cytochrome C was not degraded.

Since these data are essential for the conclusion that ABHD11 directly influences OGDHc lipoylation, these data should be transferred to the main manuscript and experiments should be repeated. The localization of ABHD11 should be confirmed using a second method (e.g. digitonin extraction, super resolution microscopy…). How were mitoblasts isolated?

We thank the reviewer for their helpful comments. We have included the mitochondrial localisation studies within the main manuscript (**New Figure 3**). Unfortunately, due to lockdown of the laboratory over the COVID-19 pandemic we have not be able to run the super resolution microscopy studies, and we agree that this may be of interest in future studies. However, prior mass spectrometry protein mapping (PMID 27499296) report ABHD11 as a matrix protein, which interacts with OGDH with high confidence. We also include our own unbiased mass spectrometry studies of ABHD11-HA, demonstrating that it interacts with OGDH and DLST (**New Table S1**). We have altered the text in the discussion to reflect these observations, which do provide independent verification that OGDH is a matrix protein.

We disagree regarding the proteinase K assay. Degradation of the soluble IMS protein cytochrome C would not be expected in intact mitochondria (**Figure 3d**). Cytochrome C was washed out on the perforation of the outer membrane involved in production of mitoplasts, which is to be expected. Importantly, ABHD11, like OGDH, remains in the mitoplast fraction even when treated with proteinase K.

2. Authors propose that ABHD11 prevents lipoyl adduct formation with lipid peroxidation products. However, this is just shown by reduced detection in immunoblots. Authors screened for several DLST intermediates which did not account for the peptide loss. Did they also screen for the 4-HNE adduct? Is this adduct detectable in ABHD11 knockout cells and wild-type with and without HNE treatment.

We have screened for 4-HNE adducts and have not been able to detect them by mass spectrometry (**Supplementary File 2**). We have also treated cells and extracts with 4-HNE and have not be able to detect adducts. The hydrophobic nature of the adducts is likely to prevent detection by mass spectrometry, and it is currently not technically possible to pursue this approach. Please also see comments to Reviewers 1 and 2.

It is important to clarify that 4HNE may not be the only lipoylated adduct formed, and we accept that other modifications may impair function DLST lipoylation and result in 2-OG accumulation. We have altered the manuscript to reflect this (see revised Discussion), and emphasise that while the requirement for ABHD11 for normal OGDHc function is clear, the mechanism of how it controls lipoylation is the subject of our ongoing studies.

3. ABHD11 cells seem to have a severe defect in mitochondrial function. Do these cells show normal growth. Is ABHD11-deficiency associated with increased lactate production?

ABHD11 loss does seem to have some toxicity to cells, as we can isolate mixed knockout populations by CRISPR, but isolation of clones was not possible. To further investigate the effect of ABHD11 on cell growth we have used ABHD11 inhibition. Treatment with ML226 does not alter cell growth over 4 days in HeLa cells cultured in DMEM (**New Figure S8a**).

We have also measured lactate production by mass spectrometry and ABHD11 loss does not result in increased lactate production, in contrast to impaired PDHc activity (**New Figure S6h**).

4. The discussion section is very short. I would be interesting to discuss why ABHD11 activity is required for this process.  Furthermore, ABHD11 is also among the genes deleted in Williams-Beuren Syndrome. Are data about mitochondrial function in this disease available?

We have now extended the discussion, including a discussion of why ABHD11 activity is required for 2-OG metabolism, and its potential role in Williams-Beuren syndrome (see also comments to Reviewer 3).

5. Statistical analysis is not shown for all experiments (e.g. Fig.2h)

Statistical analysis is now included and we apologise for these omissions.

Minor:

1. The text is confusing due to the high number of abbreviations. Try to reduce abbreviation in the text and provide full name for all abbreviations and an abbreviation list.

We have reduced the number of abbreviations, and we have included an abbreviations list.

2. labels of Fig.S1g are displaced

This has been corrected.

3. use same abbrevations in text and figures (e.g. figure 1i)

The abbreviations are now consistent between the text and figures.

**Reviewer comments, second version:**

Reviewer #1 (Remarks to the Author):

The authors have largely addressed my questions. This is a nice body of work that will be of interest to a wide audience. One point remains that requires additional discussion (but not additional experimentation). In my initial review, I asked why the increase in 2OG favored an increase in L2HG sufficient to overwhelm the effect of elevated 2OG on PHD enzyme activity and promote HIF stability. The authors added helpful discussion about how reductive and/or acidic environments promotes reduction of 2OG to L2HG. However, the question remains as to which of these stimuli apply in ABDH11 KO cells—are these cells experiencing reductive stress?

Absent some such stimulus altering the equilibrium between 2OG and L2HG (and favoring relative accumulation of L2HG over 2OG), it’s not clear to this reviewer why the net effect of increased 2OG driven by OGDH inhibition would be to block activity of 2OG-dependent dioxygenases. Certainly this remains an open question in many contexts—for example, while the addition of dm-2OG promoting HIF stability is well documented, many other situations favoring 2OG accumulation result in the more predictable effect of 2OG-dependent dioxygenase activation. Therefore I do not think the authors need to (or can) resolve this question in the context of ABDH11 KO cells, but it is important that they provide some context for readers to understand the issues surrounding the paradoxical inhibition of 2OG-dependent enzymes despite a significant increase in 2OG. Do the authors speculate that the conversion of 2OG to L2HG is favored in ABDH11 KO cells (because of reductive/acidic/other stress)? Or are there potential biochemical reasons relating to PHD Ki/Km explaining why an equimolar increase in both L2HG and 2OG would favor enzyme inhibition?


Reviewer #2 (Remarks to the Author):

The authors did a great job addressing my concerns.


Reviewer #4 (Remarks to the Author):

Authors considered reviewer concerns and provide new data supporting their hypothesis. I have no additional comments.

**Author rebuttal, second version:**

Reviewer #1 (Remarks to the Author):

The authors have largely addressed my questions. This is a nice body of work that will be of interest to a wide audience. One point remains that requires additional discussion (but not additional experimentation). In my initial review, I asked why the increase in 2OG favored an increase in L2HG sufficient to overwhelm the effect of elevated 2OG on PHD enzyme activity and promote HIF stability. The authors added helpful discussion about how reductive and/or acidic environments promotes reduction of 2OG to L2HG. However, the question remains as to which of these stimuli apply in ABDH11 KO cells—are these cells experiencing reductive stress?

Absent some such stimulus altering the equilibrium between 2OG and L2HG (and favoring relative accumulation of L2HG over 2OG), it’s not clear to this reviewer why the net effect of increased 2OG driven by OGDH inhibition would be to block activity of 2OG-dependent dioxygenases. Certainly this remains an open question in many contexts—for example, while the addition of dm-2OG promoting HIF stability is well documented, many other situations favoring 2OG accumulation result in the more predictable effect of 2OG-dependent dioxygenase activation. Therefore I do not think the authors need to (or can) resolve this question in the context of ABDH11 KO cells, but it is important that they provide some context for readers to understand the issues surrounding the paradoxical inhibition of 2OG-dependent enzymes despite a significant increase in 2OG. Do the authors speculate that the conversion of 2OG to L2HG is favored in ABDH11 KO cells (because of reductive/acidic/other stress)? Or are

there potential biochemical reasons relating to PHD Ki/Km explaining why an equimolar increase in both L2HG and 2OG would favor enzyme inhibition?

We are pleased that that our revisions addressed this reviewer’s questions, aside from an additional discussion point.

In the majority of cell lines that we have analysed, impaired OGDHc activity (through genetic disruption or germline mutations) leads to a reductive environment and L-2-HG accumulation. This is most likely to occur with ABHD11 loss as well. This reviewer rightly points out that the paradoxical inhibition of 2-OG dependent dioxygenases by L-2-HG is not fully understood. It is possible that L-2-HG may actually allosterically inhibit PHD enzymes, or that there is an additional signal (akin to succinate inhibition and ROS for HIF-1α) that is as yet unidentified. This is an area we are actively pursuing but, as the reviewer notes, is beyond the scope of this paper. We have included a further section within the discussion to reflect the open questions that remain surrounding L-2-HG inhibition, and why it may not be observed in other settings (e.g. stem cell pluripotency).

Reviewer #2 (Remarks to the Author):

The authors did a great job addressing my concerns.

Reviewer #4 (Remarks to the Author):

Authors considered reviewer concerns and provide new data supporting their hypothesis. I have no additional comments.

We thank Reviewer 2 and 4 for their appreciation of the revisions to the manuscript.

**Reviewer comments, third version:**

**Author rebuttal, third version:**
